# Unrestrictive identification of post-translational modifications in the urine proteome without enrichment

**DOI:** 10.1186/1477-5956-11-1

**Published:** 2013-01-14

**Authors:** Liu Liu, Xuejiao Liu, Wei Sun, Mingxi Li, Youhe Gao

**Affiliations:** 1 National Key Laboratory of Medical Molecular Biology, Department of Physiology and Pathophysiology, Institute of Basic Medical Sciences, Chinese Academy of Medical Sciences/School of Basic Medicine, 5 Dong Dan San Tiao, Beijing, China; 2 Department of Nephrology, Peking Union Medical College Hospital, 1 Dongcheng District Shuai Fu Yuan, Beijing, China; 3 Department of Core Instrument Facility, Institute of Basic Medical Sciences, Chinese Academy of Medical Sciences, Peking Union Medical College, Beijing, 100005, China

**Keywords:** Urine proteomics, MODa, PEAKS 6, PTMs without enrichment, *In vivo* PTMs

## Abstract

**Background:**

Research on the human urine proteome may lay the foundation for the discovery of relevant disease biomarkers. Post-translational modifications (PTMs) have important effects on the functions of protein biomarkers. Identifying PTMs without enrichment adds no extra steps to conventional identification procedures for urine proteomics. The only difference is that this method requires software that can conduct unrestrictive identifications of PTMs. In this study, routine urine proteomics techniques were used to identify urine proteins. Unspecified PTMs were searched by MODa and PEAKS 6 automated software, followed by a manual search to screen out *in vivo* PTMs by removing all *in vitro* PTMs and amino acid substitutions.

**Results:**

There were 75 peptides with 6 *in vivo* PTMs that were found by both MODa and PEAKS 6. Of these, 34 peptides in 18 proteins have novel *in vivo* PTMs compared with the annotation information of these proteins on the Universal Protein Resource website. These new *in vivo* PTMs had undergone methylation, dehydration, oxidation, hydroxylation, phosphorylation, or dihydroxylation.

**Conclusions:**

In this study, we identified PTMs of urine proteins without the need for enrichment. Our investigation may provide a useful reference for biomarker discovery in the future.

## Background

Research on urine proteomics is important for the discovery of disease biomarkers. Post-translational modifications (PTMs) of proteins regulate many physiological functions. For example, acetylation is an important PTM in metabolism regulation; phosphorylation is an important PTM in regulating enzyme activity in cellular signaling pathways; oxidation is an important marker of cellular aging; and methylation is an important PTM in the regulation of gene expression. PTMs of proteins are subject to change, and these proteins may be potential disease biomarkers. As reported previously, in patients with diabetes, there are many advanced glycation end-product peptides in urine [1,2]. The urine glycoproteomic makeup is altered in patients with chronic kidney diseases [3]. It has been shown that changes in osteopontin PTMs in urine are related to kidney stones and ovarian cancer [4,5]. Further, 2D-gels have demonstrated that there are different molecular masses of the same protein in the urine proteome [6]. Mass spectrometric immunoassays of urine protein phenotypes have also revealed a novel glycated end product of β-2-microglobulin [7].

Previous studies of urine protein PTMs have focused primarily on glycosylation, in which the proteins were first enriched via glycosylation and then identified as glycosylated proteins [8-10]. With enrichment, PTMs can be detected with high sensitivity. Research on other types of PTMs has been limited by the lack of enrichment methods [11] because each method can only identify one type of PTM. In the present study, instead of enriching for any specific PTMs, conventional urine proteomics techniques were used, and unspecified PTMs of urine proteins were identified with the MODa and PEAKS 6 software. Without enrichment, sensitivity to identify the PTMs is low. Thus far, only one previous study on urine proteomics reported the identification of phosphorylated proteins without enrichment [12].

In conjunction with recent developments in PTM research, dozens of expert algorithms have been created to perform unrestrictive searches of protein PTMs that can find almost all known PTMs and even novel PTMs. In this study, the PTM algorithms in the software packages MODa and PEAKS were used. MODa enables fast “multi-blind” unrestrictive PTM searches with a speed that is an order of magnitude faster than other existing approaches. It can also identify any number of modifications on a single peptide. In contrast to alternative approaches, MODa simultaneously uses multiple sequence tags from each MS/MS spectrum and a dynamic programming algorithm to identify modifications between sequence tags matched to a database peptide [13]. PEAKS PTM is an improved software tool for peptide identification with unspecified PTMs. The improvements in this software include a default setting whereby the software considers all PTMs included in the Universal Protein Resource (Unimod) database as variable PTMs. Moreover, several search strategies are employed to reduce the search time [14]. PEAKS PTM was included in the PEAKS 6 software, which is the only commercial software that can identify unspecified variable PTMs.

## Results

### PTMs identified by MODa and PEAKS 6

In this study, real *in vivo* PTMs were isolated from other PTMs including *in vitro* PTMs and amino acid substitutions by a manual search; the *in vitro* PTMs are mostly created during experimental processes. In all, 39,144 spectra with 6,194 unique peptides and 1,994 proteins were identified by MODa. Among these, 7,100 spectra with 1,602 unique peptides and 734 proteins contained PTMs with sizes accepted by the MODa search regardless of the modification classification in Unimod. Within these PTMs, 433 spectra with 169 unique peptides and 85 proteins had *in vivo* PTMs. Furthermore, 47,857 spectra with 9,878 unique peptides and 1,606 protein groups were identified by PEAKS 6. Among these, 20,329 spectra with 3,891 unique peptides and 1,578 proteins had PTMs with sizes accepted by the PEAKS 6 search regardless of the modification classification in Unimod. Within these PTMs, 880 spectra with 254 unique peptides and 182 protein groups had *in vivo* PTMs. These findings are summarized in Table 1.

**Table 1:** A summary of the spectra, unique peptides, and protein numbers

	**Software**	**#PSMs(Peptide Spectrum Match)**	**#Peptides**	**#Proteins**
**Whole urine**	MODa	39,144	6,194	1,994
	PEAKS 6	47,857	9,878	1,606^a^
**PTMs**	MODa	7,100	1,602	734
	PEAKS 6	20,329	3,891	1,578
***In vivo *PTMs**	MODa	433	169	85
	PEAKS 6	880	254	182
***Percentage of in vivo PTMs in whole urine*(%)**	MODa	1.106	2.728	4.263
	PEAKS 6	1.839	2.571	11.333

^a^In PEAKS 6, a protein represents a group of proteins sharing all identified peptides.

In this search, 15 types of *in vivo* PTMs were identified by MODa, and 10 types of *in vivo* PTMs were identified by PEAKS 6 (Table 2).

**Table 2:** **A summary of the names, modification sizes, and modification sites of all the *in vivo *PTMs, as well as the number of spectra, unique peptides, and proteins with *in vivo *PTMs**

***In vivo *PTMs**	**Software**	**△Mass**	**Position**	**#PSMs**	**#Peptides**	**#Proteins**
Oxidation or Hydroxylation	MODa	16	CDKNPRY	204	105	40
	PEAKS 6	15.99	DKNPRY	224	139	71
Methylation	MODa	14	CDEHKNSQRT	106	58	27
	PEAKS 6	14.02	DEILNT, C-term, N-term	157	160	99
Dehydration	MODa	−18	ST	26	19	14
	PEAKS 6	−18.01	STY	102	81	67
Dihydroxy	MODa	32	CLMPT	29	1	10
	PEAKS 6	31.99	KPRY	32	48	30
Phosphorylation	MODa	80	DS	20	8	2
	PEAKS 6	79.97	ST	192	154	57
Acetylation	MODa	42	STM(Protein N-term)	7	5	5
	PEAKS 6	42.01	CST, Protein N-term	39	41	46
Hydroxymethyl	MODa	30	N	3	1	1
Pyrophosphorylation		160	S	16	2	1
Lysine oxidation to aminoadipic semialdehyde		−1	K	12	6	6
Deamidation		1	R	7	4	4
Didehydro		−2	SY	6	1	1
HexNAc	PEAKS 6	203.08	NST	141	19	43
Carboxylation		43.99	E	10	6	7
Persulfide		31.97	D	7	6	8
Hexose		162.05	T, N-term	4	14	14

The peptides with *in vivo* PTMs as found by MODa and PEAKS 6 are presented in Additional file 1 and Additional file 2. The whole urine peptides identified by MODa and PEAKS 6 are presented in Additional file 3 and Additional file 4.

### PTMs identified by both MODa and PEAKS 6

The peptides with *in vivo* PTMs identified by both MODa and PEAKS 6 were screened out because the proteins identified as containing these peptides were somewhat different between the two software packages. Table 3 shows the peptides and corresponding proteins identified by both software packages. Table 4 shows the peptides identified by both software packages and the corresponding proteins identified by either of the two. The *in vivo* PTMs of the proteins identified by both software packages were compared with the PTM information in Uniprot, and some new PTMs were found.

**Table 3:** **The peptides with *in vivo *PTMs identified by both MODa and PEAKS 6 34 unique peptides of 18 proteins had new PTMs (labeled by underline) compared to the Uniprot annotation information**

**Peptide (identified by both software packages)**	**Protein^b^**	**Peptide Position**	**Description**
R.SYSCQVTHEGSTVEK[Methylation].T	sp|B9A064	192 ~ 206	Immunoglobulin lambda-like polypeptide 5 OS = Homo sapiens GN = IGLL5 PE = 2 SV = 2
M.T[Acetylation]DGDYDYLIK.L	sp|O00194	2 ~ 11	Ras-related protein Rab-27B OS = Homo sapiens GN = RAB27B PE = 1 SV = 4
K.GDAGPP[Hydroxylation]GPAGPAGPPGPI.G	sp|P02452	836 ~ 862	Collagen alpha-1(I) chain OS = Homo sapiens GN = COL1A1 PE = 1 SV = 5
K.GDAGPP[Hydroxylation]GPAGPAGPPGPIGNVGAPGAK.G			
R.EGAPGAEGSP[Hydroxylation]GR.D		1015 ~ 1026	
K.DGEAGAQGPP[Hydroxylation]GPAGPAGER.G		613 ~ 631	
R.DGNP[Hydroxylation]GSDGLPGR.D	sp|P02461	1013 ~ 1024	Collagen alpha-1(III) chain OS = Homo sapiens GN = COL3A1 PE = 1 SV = 4
R.DGNPGSDGLP[Hydroxylation]GR.D			
R.TVAACNLPIVR[Methylation].G	sp|P02760	283 ~ 293	Protein AMBP OS = Homo sapiens GN = AMBP PE = 1 SV = 1
K.N[Oxidation or Hydroxylation]WGLSVYADKPETTK.E	sp|P02763	139 ~ 153	Alpha-1-acid glycoprotein 1 OS = Homo sapiens GN = ORM1 PE = 1 SV = 1
K.AGVET[Dehydration]TTPSK.Q	sp|P0CG05	51 ~ 60	Ig lambda-2 chain C regions OS = Homo sapiens GN = IGLC2 PE = 1 SV = 1
N.AMQVINNYQR[Methylation].R	sp|P10153	53 ~ 62	Non-secretory ribonuclease OS = Homo sapiens GN = RNASE2 PE = 1 SV = 2
R.WGYSSTAITR[Methylation].Q	sp|P10253	376 ~ 385	Lysosomal alpha-glucosidase OS = Homo sapiens GN = GAA PE = 1 SV = 4
K.TGPIGPQGAP[Hydroxylation]GK.P	sp|P20908	1422 ~ 1433	Collagen alpha-1(V) chain OS = Homo sapiens GN = COL5A1 PE = 1 SV = 3
R.HS[Dehydration]PQEAPHVQYER.L	sp|P26992	25 ~ 37	Ciliary neurotrophic factor receptor subunit alpha OS = Homo sapiens GN = CNTFR PE = 1 SV = 2
R.LGPGMADICK[Methylation].N	tr|B1AVU8	233 ~ 242	Proactivator polypeptide OS = Homo sapiens GN = PSAP PE = 4 SV = 1
K.AIPVAQDLNAPSDWDS[Phosphorylation]R.G	tr|B2RDA1	190 ~ 206	**`Osteopontin OS = Homo sapiens GN = SPP1 PE = 2 SV = 1**
K.ANDES[Phosphorylation]NEHSDVIDSQELSK.V		236 ~ 254	
K.YPDAVATWLNPDPSQK[Methylation].Q		36 ~ 51	
R.GKDS[Phosphorylation]YETSQLDDQSAETHSHK.Q		207 ~ 227	
K.AAT[Dehydration]GECTATVGK.R	tr|B4DPP8	90 ~ 101	**Kininogen-1 OS = Homo sapiens GN = KNG1 PE = 2 SV = 1**
K.LGQSLDCN[Oxidation or Hydroxylation]AEVYVVPWEK.K		333 ~ 350	
K.YNSQNQSNNQFVLYR[Methylation].I		32 ~ 46	
R.GPWCY[Oxidation or Hydroxylation]VSGEAGVPEK.R	tr|B4DRR9	79 ~ 93	HGFL OS = Homo sapiens GN = PIK3IP1 PE = 2 SV = 1
R.GPWCYVSGEAGVPEK[Methylation].R			
K.CVN[Oxidation or Hydroxylation]HYGGYLCLPK.T	tr|B4DW75	3 ~ 15	EGF containing fibulin-like extracellular matrix protein 1 OS = Homo sapiens GN = EFEMP1 PE = 2 SV = 1
R.TSSYLCQYQCVN[Oxidation or Hydroxylation]EPGK.F		162 ~ 177	
K.QNLLAPQNAVS[Phosphorylation]SEETNDFKQETLPSK.S	tr|C9JXD2	52 ~ 77	Osteopontin OS = Homo sapiens GN = SPP1 PE = 4 SV = 1 Epidermal growth factor OS = Homo sapiens GN = EGF PE = 4 SV = 1
K.QNLLAPQNAVSS[Phosphorylation]EETNDFK.Q		52 ~ 70	
K.CIN[Oxidation or Hydroxylation]TEGGYVCR.C	tr|E7EVD2	888 ~ 898	
N.SSCVN[Oxidation or Hydroxylation]TPGSFSCVCPEGFR.L	tr|E9PEA4	114 ~ 132	Uromodulin_ secreted form OS = Homo sapiens GN = UMOD PE = 4 SV = 1
R.D[Oxidation or Hydroxylation]WVSVVTPAR.D		409 ~ 418	
R.DGPCGT[Dehydration]VLTR.N		419 ~ 428	
R.MAETCVPVLR[Methylation].C		246 ~ 255	
R.STEYGEGYACDT[Dehydration]DLR.G		219 ~ 233	
T.CVN[Oxidation or Hydroxylation]VVGSYLCVCPAGYR.G		159 ~ 175	
V.N[Oxidation or Hydroxylation]VVGSYLCVCPAGYR.G		161 ~ 175	
K.FEHCNFNDVTTR[Methylation].L	tr|E9PNW4	67 ~ 78	CD59 glycoprotein OS = Homo sapiens GN = CD59 PE = 4 SV = 1
R.LRENELT[Dehydration]YYCCK.K		79 ~ 90	
R.LRENELTYYCCK[Methylation].K			
R.YPNQVYYR[Methylation].P	tr|F5GY30	96 ~ 103	Major prion protein OS = Homo sapiens GN = PRNP PE = 3 SV = 1
K.EGNPGPLGPIGP[dihydroxy]PGVR.G	tr|H7C157	827 ~ 842	**Collagen alpha-2(V) chain OS = Homo sapiens GN = COL5A2 PE = 4 SV = 1**

^b^If one peptide with *in vivo* PTMs was identified by both software packages, and the corresponding protein in MODa belonged to the same protein group in PEAKS 6, then the protein in MODa belonged to a corresponding protein identified by both software packages.

**Table 4:** The peptides identified by both software packages, and the corresponding proteins identified by either of the two software packages

**Protein group (PEAKS PTM)cc**	**Peptide (Both)**	**Protein (MODa)c**
5	K.AAT[Dehydration]GECTATVGK.R	
8		
49	K.AGAAAGGP[Oxidation or Hydroxylation]GVSGVCVCK.S	tr|B4E1N2
75	K.AGVET[Dehydration]TTPSK.Q	
15	K.AIPVAQDLNAPSDWDS[Phosphorylation]R.	
22	G	
36		
15	K.ANDES[Phosphorylation]NEHSDVIDSQELS	
22	K.V	
3	K.CCAAADPHECYAK[Methylation].V	tr|A6NBZ8
2		
1		
500	K.CIN[Oxidation or Hydroxylation]HYGGYLCLPR.S	tr|E9PKA3
90	K.CVN[Oxidation or Hydroxylation]HYGGYLCLPK.T	
369		
249	K.DGETGAAGPP[Oxidation or Hydroxylation]GPAGPAGER.G	tr|G8JLI4
15		
22		
36		
21		
304	K.EGNPGPLGPIGP[dihydroxy]PGVR.G	
100	K.EGPVGLP[Oxidation or Hydroxylation]GIDGR.P	tr|F5H299
285	K.FELTGIPPAPR[Methylation].G	tr|A8K7Q2
1	K.FQNALLVR[Methylation].Y	tr|A6NBZ8
3		
2		
21		
15		
22		
36		
100	K.EGPVGLP[Oxidation or Hydroxylation]GIDGR.P	tr|F5H299
249	K.GEVGPP[Oxidation or Hydroxylation]GPAGSAGAR.G	tr|G8JLI4
	K.GPP[Oxidation or Hydroxylation]GPQGPAGEQGPR.G	
5	K.LGQSLDCN[Oxidation or Hydroxylation]AEVYVVPWEK.K	
8		
28	K.LHNLNSN[Oxidation or Hydroxylation]WFPAGSK.P	tr|B3KTI1
37		
31		
103	K.NGETGPQGPP[Oxidation or Hydroxylation]GPTGPGGDK.G	tr|E7ENY8
8	K.QNLLAPQNAVSS[Phosphorylation]EETNDFK.Q	tr|A6NBZ8
3	K.VHTECCHGDLLECADD[Methylation]R.A	
2		
1		
3	K.VHTECCHGDLLECADDR[Methylation].A	
2		
1		
5	K.YNSQNQSNNQFVLYR[Methylation].I	
8		
15	K.YPDAVATWLNPDPSQK[Methylation].Q	
22		
957	M.S[Acetylation]SSGTPDLPVLLTDLK.I	tr|E7ER57
100	P.GIAGHHGDQGAP[Oxidation or Hydroxylation]GSVGPAGPR.G	tr|F5H299
34	R.ALVFVDNHDNQR[Methylation].G	tr|B3KTI1
28		
37		
31		
13	R.AVLPQEEEGS[Dehydration]GGGQLVTEVTK.K	tr|B7Z8R6
10	R.CKPVNTFVHEPLVDVQNVCFQE[Methylation]K.V	tr|G3V357
48	R.CVN[Oxidation or Hydroxylation]TYGSYECK.C	tr|F5H2N7
1	R.ETYGEMADCCAK[Methylation].Q	tr|A6NBZ8
15	R.GKDS[Phosphorylation]YETSQLDDQSAETHSHK.Q	
36		
100	R.GLHGEFGLP[Oxidation or Hydroxylation]GPAGPR.G	
	R.GPP[Oxidation or Hydroxylation]GESGAAGPTGPIGSR.G	
	R.GPSGPP[Oxidation or Hydroxylation]GPDGNK.G	
	
103	R.GPTGPIGPP[Oxidation or Hydroxylation]GPAGQPGDK.G	tr|E7ENY8
13	R.HHGPT[Dehydration]ITAK.L	tr|B7Z8R6
10	R.HIIVACEGS[Dehydration]PYVPVHF.D	tr|G3V357
8	R.HS[Dehydration]PQEAPHVQYER.L	
11	R.LGPGMADICK[Methylation].N	
19	R.NPDSSTTGP[dihydroxy]WCYTTDPTVR.R	tr|C9JQ37
20		
19	R.SGIECQLWR[Methylation].S	
20		
84	R.SYSCQVTHEGSTVEK[Methylation].T	
73		
100	R.TGEVGAVGP[Oxidation or Hydroxylation]PGFAGEK.G	tr|F5H299
	R.TGEVGAVGPP[Oxidation or Hydroxylation]GFAGEK.G	
90	R.TSSYLCQYQCVN[Oxidation or Hydroxylation]EPGK.F	
13	R.VVAQGVGIPEDSIFT[Dehydration]MADR.G	tr|B7Z8R6
16	S.LQCYNCPNPTADCK[Methylation].T	tr|E9PI80

cIn PEAKS 6, one peptide can belong to several protein groups. In contrast, in MODa, one peptide can only belong to one protein.

The peptides identified by both software packages had 6 types of *in vivo* PTMs, which are shown in Table 5. In PEAKS 6, one peptide can belong to several protein groups. In contrast, in MODa, one peptide can only belong to one protein.

**Table 5:** **The *in vivo *PTMs identified by both software packages and the number of peptides and proteins**

***In vivo *PTMs**	**#Peptides**	**#Proteins**
Oxidation or Hydroxylation	34	10
Methylation	22	11
Dehydration	10	5
Dihydroxy	2	1
Phosphorylation	5	2
Acetylation	2	1
All	75	25

The spectra of the peptides with *in vivo* PTMs that were identified most reliably by both software packages are listed in Additional file 5, and only one spectrum per peptide is listed.

## Discussions

This is the first study of its kind to identify post-translational modifications in the urine proteome without preferential enrichment, using a mixture of 12 human urine samples (6 males and 6 females). The pooled sample was used to identify as many PTMs as possible in a single experiment. Because the original donors that provided the urine samples may differ in gender, age and other medical conditions, the PTMs in the urine proteomes are also likely to be different among the individuals. The PTMs in individual urine samples will be studied in the future. Moreover, the reagents from the experimental procedures including protein digestion may introduce many artifact PTMs that are not endogenous to the samples. For example, urea can cause the non-enzymatic modification of carbamylation to certain proteins. The two software packages identified both artifact PTMs and *in vivo* PTMs. We manually excluded all possible artifact PTMs and reported only the unequivocal *in vivo* PTMs.

## Conclusions

In this study, we were able to identify all urine protein PTMs without enrichment. Our investigation may provide a useful reference for biomarker discovery in the future. As the technology and algorithms for conducting proteomic screens improve, more PTMs from the urine proteome will likely be identified.

## Materials and methods

### Urine collection and preparation

Pooled urine was collected from 12 healthy donors (6 males and 6 females). The donors (without medical condition and eating behavior information) were between 20–40 years old. The midstream of the urine was collected, and the samples were stored at 4°C immediately. On the same day, the urine was centrifuged at 3,000 × g for 10 min at 4°C. After removing the precipitates, the supernatant was added to three volumes of cold acetone. It was then incubated at 4°C for 2 h, followed by centrifugation at 12,000 × g for 30 min. The precipitates were collected and air-dried at room temperature. Afterwards, lysis buffer (7 M urea, 2 M thiourea, 120 mM dithiothreitol, and 40 mM Tris) was added to resuspend the pellets, which were then quantified by the Bradford method.

### Protein digestion and peptide preparation

The urinary proteins were digested with trypsin (Trypsin Gold, Mass Spec Grade, Promega, Fitchburg, Wisconsin) by filter-aided sample preparation[15] using 10 kD Pall filtration devices (Pall Corporation, Port Washington, New York). Briefly, after urine samples were loaded into the filtration unit (200 μg per unit), 200 μL of UA buffer (8 M urea in 0.1 M Tris–HCl, pH 8.5) was added to the unit. After centrifuging the proteins at 13,000 × g for 20 min, repeat the UA wash. 200 μL of 50 mM NH_4_HCO_3_ was added, and the samples were centrifuged at 13,000 × g for 20 min, repeat the NH_4_HCO_3_ wash. Afterwards, 100 μL of 20 mM dithiothreitol in 50 mM NH_4_HCO_3_ was added to reduce the samples at 50°C for 1 h. Five microliters of 1 M iodoacetamide was added to alkylate the samples in the dark at room temperature for 30 min. After washing the filter twice with 50 mM NH_4_HCO_3_ at 13,000 × g for 20 min, trypsin (enzyme: protein ratio of 1:50) was added to digest the samples at 37°C overnight. The filtration unit was centrifuged for 20 min to collect the peptides, which were then desalted using a 1 mL OASIS HLB cartridge (Waters, Milford, MA) according to the manufacturer’s instructions. The elution was dried in a SpeedVac system (Thermo Fischer Scientific) and stored at −80°C until LC/MS/MS analysis.

### LC/MS/MS methods

The lyophilized peptides were dissolved in 0.1% formic acid and then separated by 2D LC/MS/MS using a strong cation exchange column (150 mm × 320 mm inner diameter, strong cation exchange resins from PolyLC Inc., Columbia, USA) and a reverse phase (RP) column (150 mm × 100 mm id, Michrom Bioresources, Auburn, California). One SCX elution method was used in which the ammonia acetate pH gradients during the separation and elution steps were 2.5, 3, 3.5, 4, 4.5, 5, 5.5, 6, 7, 8, and 10. For RP separation, the eluted peptides were loaded onto the column with buffer A (0.1% formic acid), and the elution gradient was 5-30% buffer B (0.1% formic acid + 99.9% ACN, flow rate: 0.5 μL/min). An LTQ-Orbitrap Velos was operated in the data-dependent acquisition mode with the XCalibur software. MS survey scan data were acquired with the Orbitrap in the 300–2,000 m/z range with the resolution set to a value of 60,000. The 20 most intense ions per survey scan were selected for CID fragmentation, and the resulting fragments were analyzed with the linear trap (LTQ). Dynamic exclusion was employed within 60 s to prevent repetitive selection of the same peptide.

### Data processing

#### Software and operating environment

MODa was obtained from the Division of Computer Science and Engineering of Hanyang University in Korea by email eunokpaek@hanyang.ac.kr. A trial version of PEAKS 6 was downloaded from the Bioinformatics Solutions website. The operating environment for MODa was a computer with 2 G RAM and an Intel® Core™2 Duo CPU E6750 @2.66 GHz 2.00 GHz. PEAKS 6 was operated on a computer with 16 G RAM and an Intel® Xeon® CPU X5650 @2.67 GHz 2.66 GHz (2 processors).

#### File conversion

The RAW files were converted to MGF files by the MM File Conversion software.

#### Database

The Uniprot human proteomics database released on 3/21/2012.

#### Parameters for the MODa search

According to the README instruction in the software folder, the parameters were set as follows:

PeptTolerance = 2.5: This parameter indicates the parent mass tolerance in Daltons.

AutoPMCorrection = [0|1]: The default parameter value is “0”, whereas “1” means that the program will automatically find the optimal parent mass for the input spectrum, regardless of the specified PeptTolerance.

FragTolerance = 0.5: This parameter indicates the fragment ion mass tolerance in Daltons.

BlindMode = 2: This parameter indicates the number of modifications per peptide, and '2' allows an arbitrary number of modifications per peptide.

MinModSize = [−200], maxModSize = [+200]: This parameter indicates the minimum and maximum modification size in Daltons (Da).

Enzyme = Trypsin, KR/C: This parameter indicates the reagent used for protein digestion as well as the cleavage sites and amino acid terminus.

MissedCleavage = [2]: This parameter indicates the number of allowed missed cleavage sites.

CysteineBlocking = Carbamidomethyl, 57: This parameter indicates the chemical derived from a free cysteine by the alkylation process and the mass of the chemical derivative.

False discovery rate (FDR) ≤ 1%: This parameter indicates the FDR of the Peptide-Spectrum Matches (PSMs).

#### Parameters for the PEAKS 6 search

The search parameters were set as follows:

Parent Mass Error Tolerance: 10.0 ppm

Fragment Mass Error Tolerance: 0.1 Da

Precursor Mass Search Type: Monoisotopic

Max Missed Cleavages: 2

Non-specific Cleavage: 1

Fixed Modifications: Carbamidomethylation: 57.02

Variable Modifications:

Deamidation (NQ): 0.98; Oxidation (M): 15.99; Pyro-glu from Q: -17.03; 4-hydroxynonenal (HNE): 156.12; Acetylation(K): 42.01; Acetylation(N-term): 42.01; Acetylation(ProteinN-term): 42.01; Amidation: -0.98; and 669 more built-in modifications in PEAKS 6

Max variable PTM per peptide: 3.

Result filtration parameters: De novo score (ALC%) threshold: 30; Peptide −10 lgP ≥ 17.5; Protein −10 lgP ≥ 20; FDR (Peptide-Spectrum Matches): 1.00%.

#### Manual search

For MODa, the observed modification size was matched with the modification name and classification on the Unimod website (http://www.unimod.org/modifications_list.php). The modification size was set as the average mass. The modification size tolerance was set as 0.05 Daltons. For PEAKS 6, the observed modification name was matched with the modification classification on the Unimod website. Some of the PTM classifications in Unimod are Artefact, Post-translational, Chemical derivative, AAsubstitution, Pre-translational, and Multiple. The PTMs that are classified as ‘Post-translational’ represent *in vivo* PTMs.

## Competing interests

There are no competing interests in this study.

## Authors' contributions

Xuejiao Liu performed the experiments described under Urine Collection and Preparation, Protein Digestion and Peptide Preparation, and LC/MS/MS Methods. Liu Liu processed the data and drafted the manuscript. Both authors read and approved the final manuscript. Youhe Gao proposed the project.

## Supplementary Material

Additional file 1 The peptides with in vivo PTMs as found by MODa. Click here for file

Additional file 2 The peptides with in vivo PTMs as found by PEAKS 6. Click here for file

Additional file 3 The whole urine peptides identified by MODa. Click here for file

Additional file 4 The whole urine peptides identified by PEAKS 6. Click here for file

Additional file 5 The spectra of the peptides with in vivo PTMs identified by both software packages. Click here for file
