# Supplementary material for: Unrestrictive identification of post-translational modifications in the urine proteome without enrichment
Source: Proteome Sci. 2013 Jan 14;11:1. doi: 10.1186/1477-5956-11-1 (PMC3585864; doi:10.1186/1477-5956-11-1)
Supplement: Additional file 5 — The spectra of the peptides with in vivo PTMs identified by both software packages. [file 1477-5956-11-1-S5.pdf]

Additional file 5

peptides sequences with in vivo PTMs	spectra number
R.GPWCYVSGEAGVPEK[Methylation].R	1
R.TVAACNLPIVR[Methylation].G	2
S.LQCYNCPNPTADCK[Methylation].T	3
R.GKDS[Phosphorylation]YETSQLDDQSAETHSHK.Q	4
R.VVAQGVGIPEDSIFT[Dehydration]MADR.G	5
R.AVLPQEEEGS[Dehydration]GGGQLVTEVTK.K	6
K.GDAGPP[Hydroxylation]GPAGPAGPPGPIGNVGAPGAK.G	7
K.EGNPGPLGPIGP[dihydroxy]PGVR.G	8
R.TGEVGAVGPP[Oxidation or Hydroxylation]GFAGEK.G	9
R.GPTGPIGPP[Oxidation or Hydroxylation]GPAGQPGDK.G	10
K.AGAAAGGP[Oxidation or Hydroxylation]GVSGVCVCK.S	11
R.DGNP[Hydroxylation]GSDGLPGR.D	12
K.GEPGSAGPQGPP[Oxidation or Hydroxylation]GPSGEEGK.R	13
R.DGNPGSDGLP[Hydroxylation]GR.D	14
K.AAT[Dehydration]GECTATVGK.R	15
K.AGVET[Dehydration]TTPSK.Q	16
R.GPSGPP[Oxidation or Hydroxylation]GPDGNK.G	17
M.S[Acetylation]SSGTPDLPVLLTDLK.I	18
K.QNLLAPQNAVSS[Phosphorylation]EETNDFK.Q	19
M.T[Acetylation]DGDYDYLIK.L	20
K.AIPVAQDLNAPSDWDS[Phosphorylation]R.G	21
K.GDAGPP[Hydroxylation]GPAGPAGPPGPI.G	22
R.CKPVNTFVHEPLVDVQNVCFQE[Methylation]K.V	23
K.YNSQNQSNNQFVLYR[Methylation].I	24
K.CIN[Oxidation or Hydroxylation]HYGGYLCLPR.S	25
K.CVN[Oxidation or Hydroxylation]HYGGYLCLPK.T	26
K.LHNLNSN[Oxidation or Hydroxylation]WFPAGSK.P	27
P.GIAGHHGDQGAP[Oxidation or Hydroxylation]GSVGPAGPR.G	28
K.FQNALLVR[Methylation].Y	29
R.LRENELTYYCCK[Methylation].K	30
K.CCAAADPHECYAK[Methylation].V	31
R.SGIECQLWR[Methylation].S	32
R.GLHGEFGLP[Oxidation or Hydroxylation]GPAGPR.G	33
R.ALVFVDNHDNQR[Methylation].G	34
R.LRENELT[Dehydration]YYCCK.K	35
K.FEHCNFNDVTTR[Methylation].L	36
R.SYSCQVTHEGSTVEK[Methylation].T	37
R.HS[Dehydration]PQEAPHVQYER.L	38
T.CVN[Oxidation or Hydroxylation]VVGSYLCVCPAGYR.G	39
K.N[Oxidation or Hydroxylation]WGLSVYADKPETTK.E	40

K.VHTECCHGDLLECADDR[Methylation].A	41
K.VHTECCHGDLLECADD[Methylation]R.A	42
V.N[Oxidation or Hydroxylation]VVGSYLCVCPAGYR.G	43
R.MAETCVPVLR[Methylation].C	44
R.LGPGMADICK[Methylation].N	45
K.CIN[Oxidation or Hydroxylation]TEGGYVCR.C	46
R.CVN[Oxidation or Hydroxylation]TYGSYECK.C	47
R.TGEVGAVGP[Oxidation or Hydroxylation]PGFAGEK.G	48
K.GPP[Oxidation or Hydroxylation]GPQGPAGEQGPR.G	49
K.TGPIGPQGAP[Hydroxylation]GK.P	50
K.GEVGPP[Oxidation or Hydroxylation]GPAGSAGAR.G	51
K.NGETGPQGPP[Oxidation or Hydroxylation]GPTGPGGDK.G	52
R.GPP[Oxidation or Hydroxylation]GESGAAGPTGPIGSR.G	53
R.DGPCGT[Dehydration]VLTR.N	54
K.ANDES[Phosphorylation]NEHSDVIDSQELSK.V	55
R.NPDSSTTGP[dihydroxy]WCYTTDPTVR.R	56
R.TSSYLCQYQCVN[Oxidation or Hydroxylation]EPGK.F	57
K.QNLLAPQNAVS[Phosphorylation]SEETNDFKQETLPSK.S	58
R.D[Oxidation or Hydroxylation]WVSVVTPAR.D	59
K.FELTGIPPAPR[Methylation].G	60
R.ETYGEMADCCAK[Methylation].Q	61
R.GPWCY[Oxidation or Hydroxylation]VSGEAGVPEK.R	62
N.SSCVN[Oxidation or Hydroxylation]TPGSFSCVCPEGFR.L	63
K.YPDAVATWLNPDPSQK[Methylation].Q	64
K.LGQSLDCN[Oxidation or Hydroxylation]AEVYVVPWEK.K	65
K.DGEAGAQGPP[Hydroxylation]GPAGPAGER.G	66
K.DGETGAAGPP[Oxidation or Hydroxylation]GPAGPAGER.G	67
R.WGYSSTAITR[Methylation].Q	68
R.EGAPGAEGSP[Hydroxylation]GR.D	69
K.EGPVGLP[Oxidation or Hydroxylation]GIDGR.P	70
N.AMQVINNYQR[Methylation].R	71
R.HHGPT[Dehydration]ITAK.L	72
R.HIIVACEGS[Dehydration]PYVPVHF.D	73
R.STEYGEGYACDT[Dehydration]DLR.G	74
R.YPNQVYYR[Methylation].P	75

1

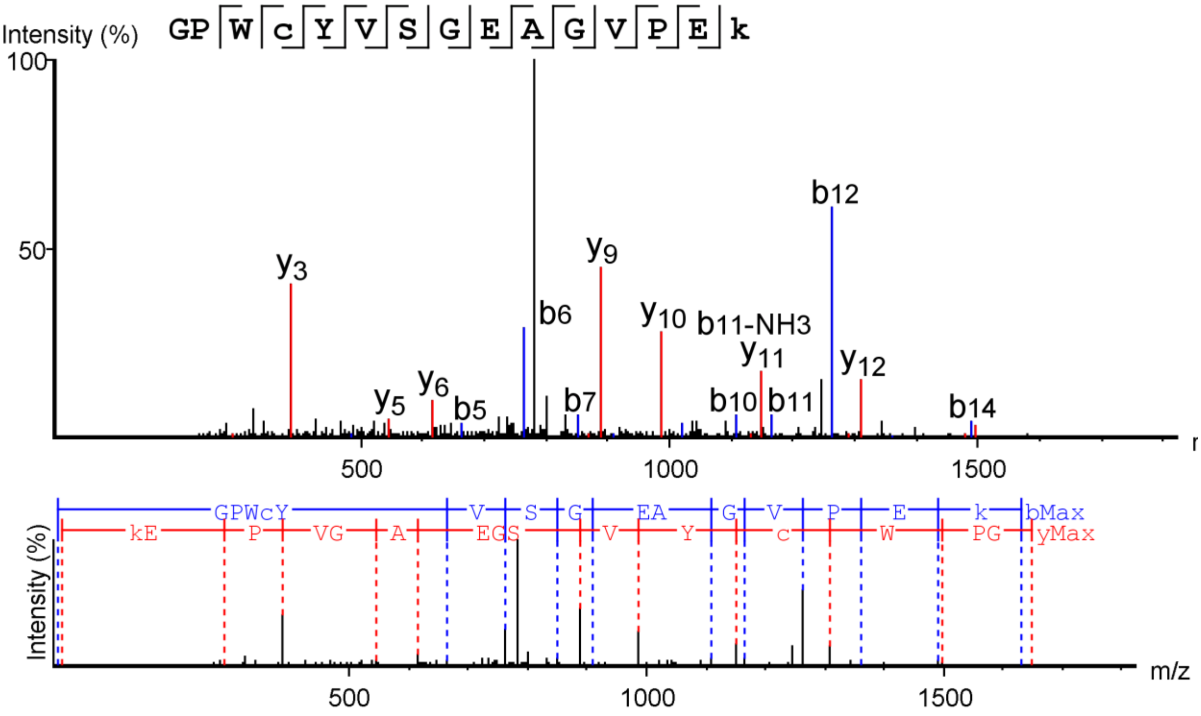

Intensity (%)
GP W c Y V S G E A G V P E k
100
50
y3
y5
y6
b5
b6
b7
y9
y10
b11-NH3
y11
b10
b11
b12
y12
b14
500
1000
1500
GPWcY V S G EA G V P E k bMax
kE P VG A EGS V Y c W PG yMax
Intensity (%)
m/z

2

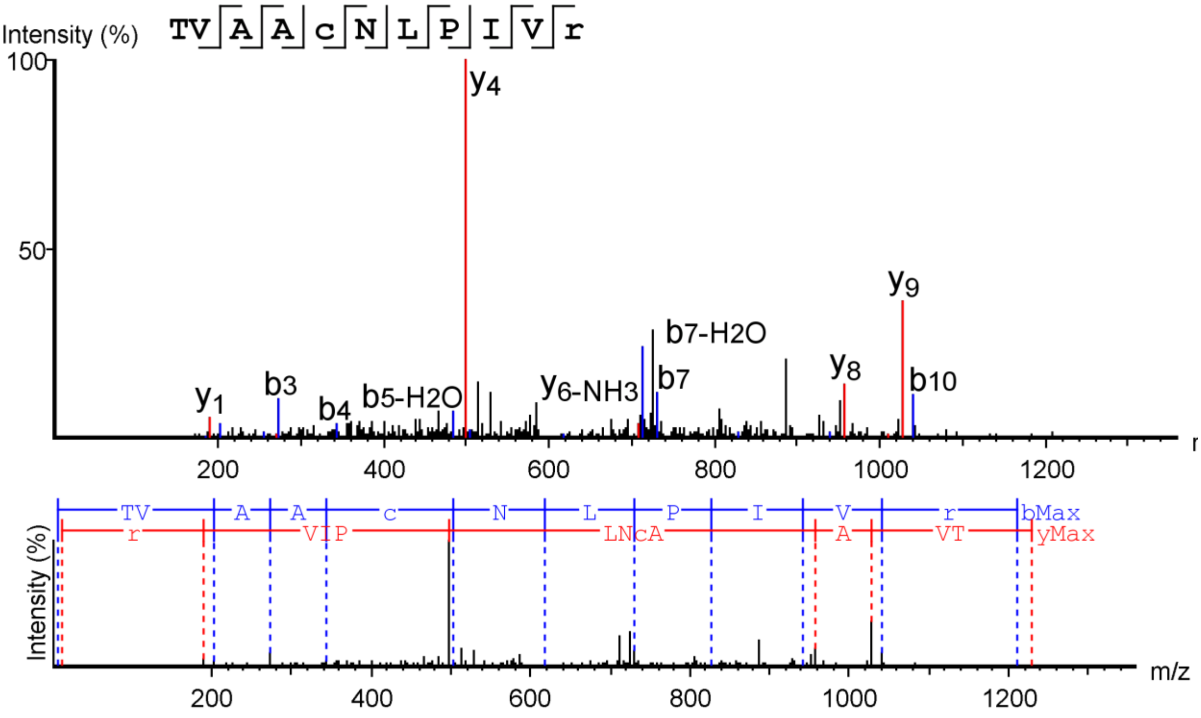

Intensity (%)
TV A A c N L P I V r
100
50
y4
y1
b3
b4
b5-H2O
y6-NH3
b7-H2O
b7
y8
y9
b10
200
400
600
800
1000
1200
TV A A c N L P I V r bMax
r VIP LNcA A VT yMax
Intensity (%)
m/z

3

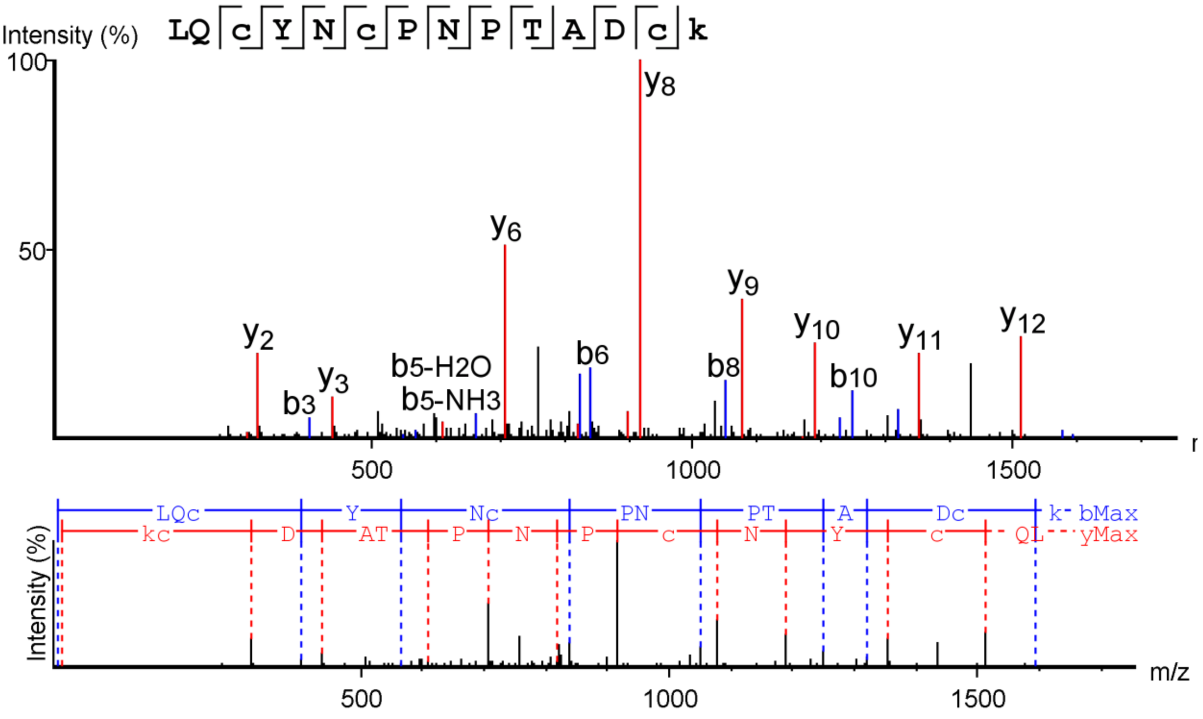
Intensity (%)
LQcYNcPNPTADck
100
50
y8
y6
y9
y10
y11
y12
y2
b3
y3
b5-H2O
b5-NH3
b6
b8
b10
500
1000
1500
LQc Y Nc PN PT A Dc k bMax
kc D AT P N P c N Y c QL yMax
Intensity (%)
m/z
500
1000
1500

4

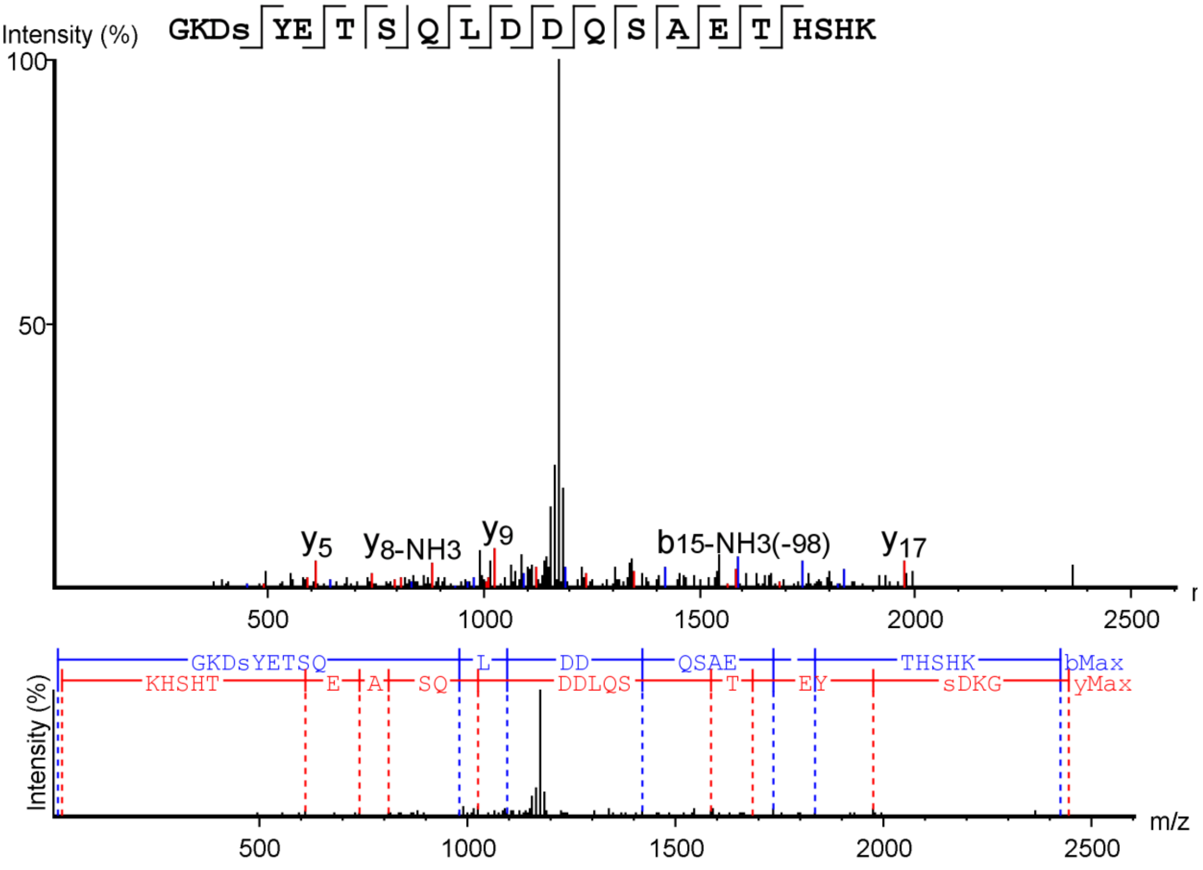
Intensity (%)
GKDsYETSQLDDQSAETHSHK
100
50
y5
y8-NH3
y9
b15-NH3(-98)
y17
500
1000
1500
2000
2500
GKDsYETSQ L DD QSAE THSHK bMax
KHSHT E A SQ DDLQS T EY sDKG yMax
Intensity (%)
m/z
500
1000
1500
2000
2500

5

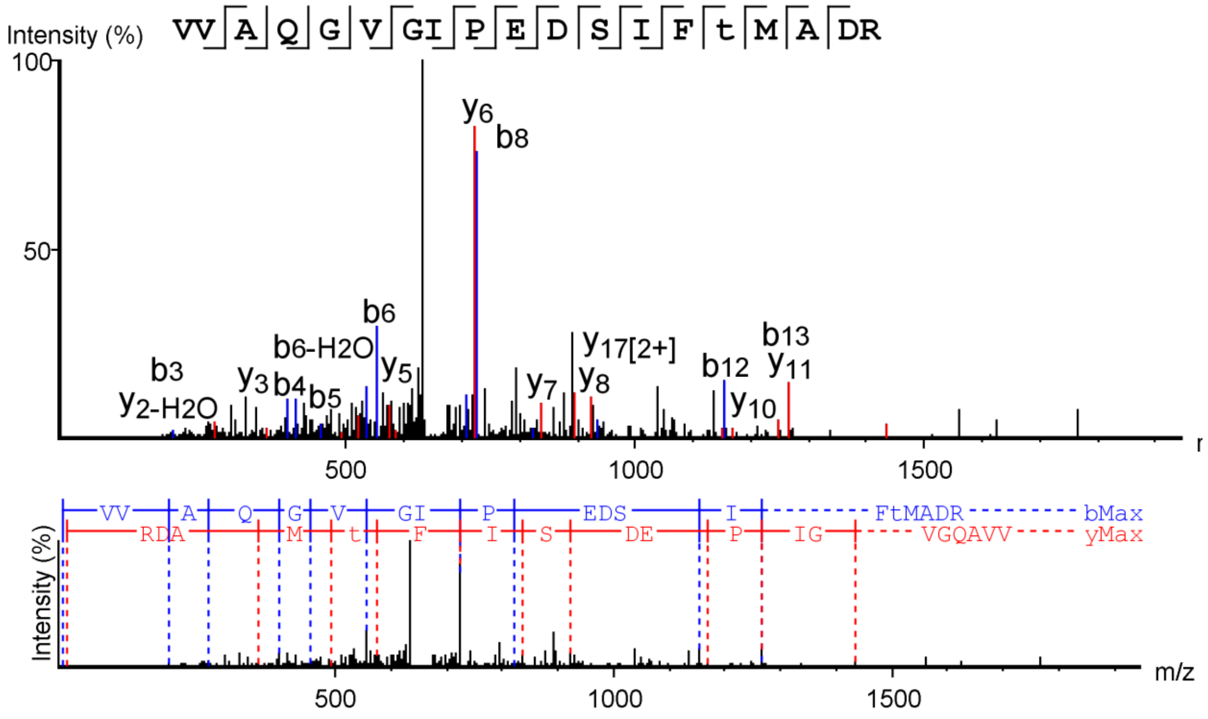
Intensity (%)
VV A Q G V GI P E D S I F t M A DR
100
50
y6
b8
b6
b6-H2O
y5
b3
y3
b4
b5
y2-H2O
y7
y17[2+]
y8
b12
b13
y11
y10
500
1000
1500
VV A Q G V GI P EDS I FtMADR bMax
RDA M t F I S DE P IG VGQAVV yMax
Intensity (%)
500
1000
1500
m/z

6

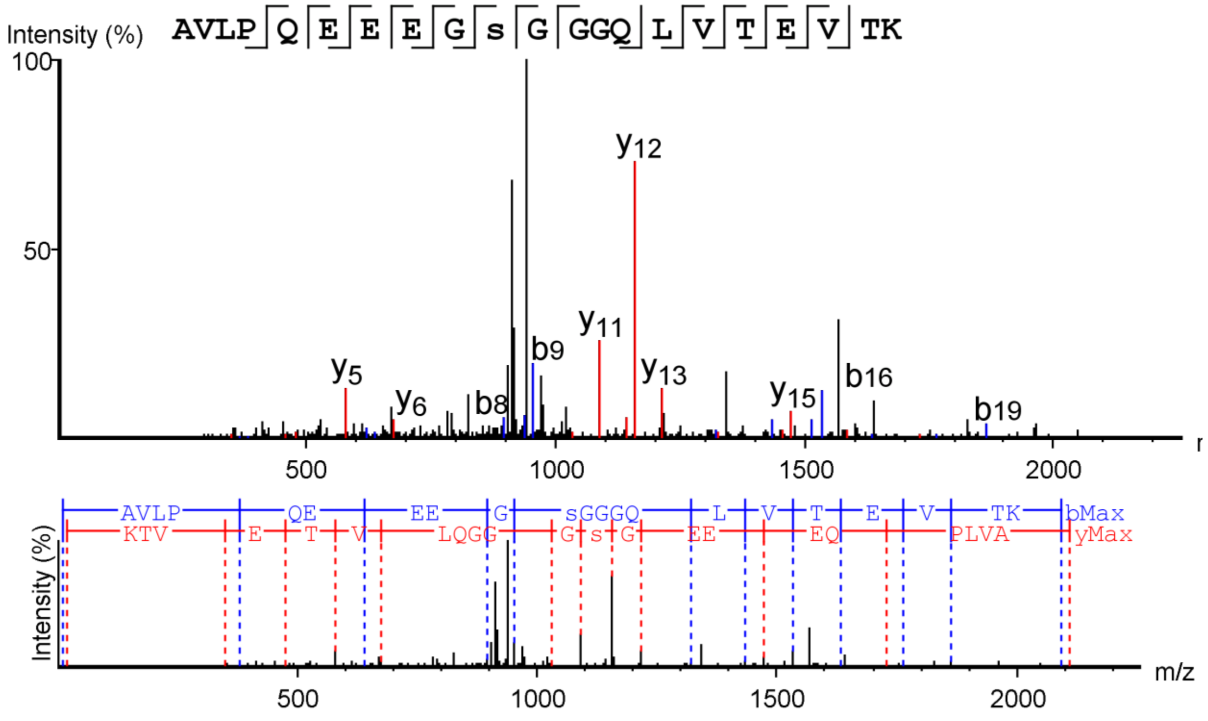
Intensity (%)
AVLP Q E E E G s G GGQ L V T E V TK
100
50
y12
y11
b9
y5
y6
b8
y13
y15
b16
b19
500
1000
1500
2000
AVLP QE EE G sGGGQ L V T E V TK bMax
KTV E T V LQGG G s G EE EQ PLVA yMax
Intensity (%)
500
1000
1500
2000
m/z

7

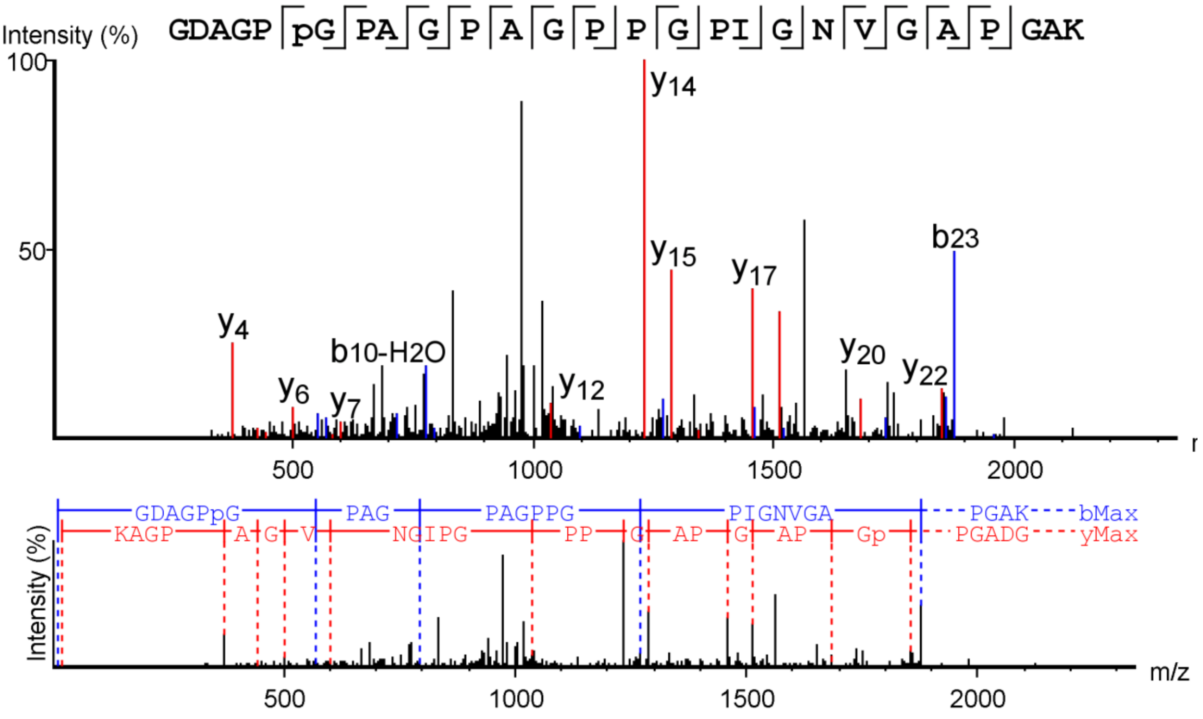
Intensity (%)
GDAGP pG PA G P A G P P G PI G N V G A P GAK
100
50
y4
y6
y7
b10-H2O
y12
y14
y15
y17
y20
y22
b23
500
1000
1500
2000
GDAGPpG PAG PAGPPG PIGNVGA PGAK bMax
KAGP A G V NGIPG PP G AP G AP Gp PGADG yMax
Intensity (%)
m/z
500
1000
1500
2000

8

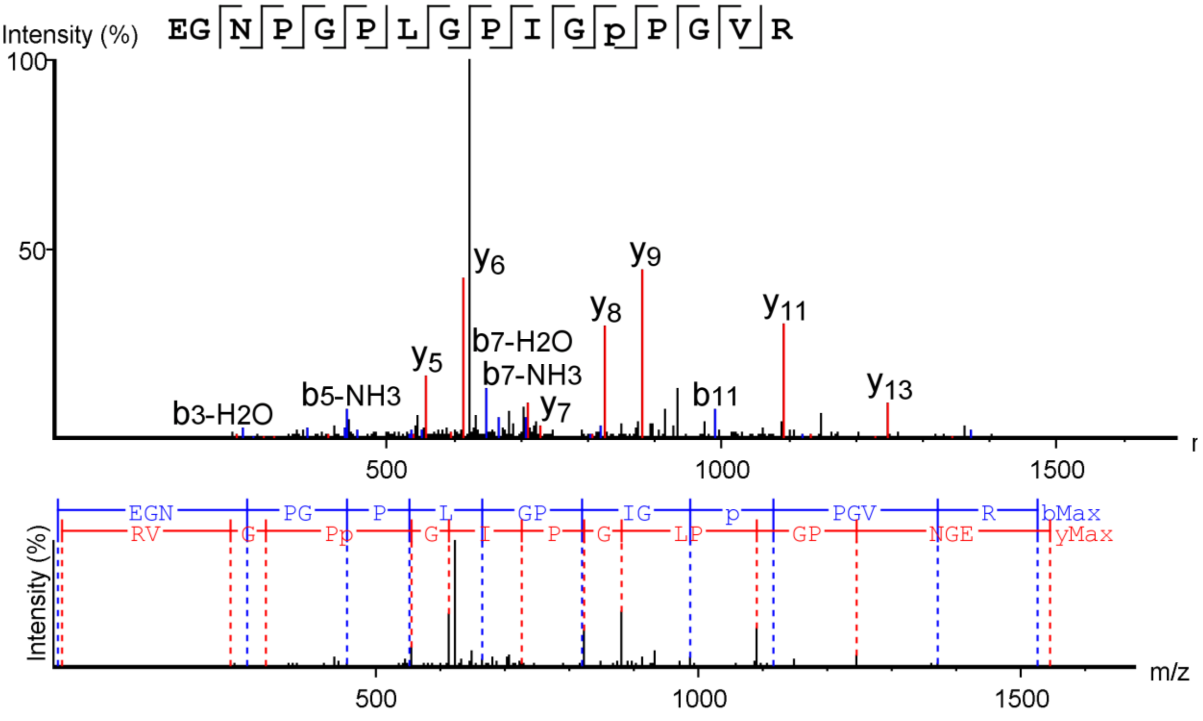
Intensity (%)
EG N P G P L G P I G p P G V R
100
50
b3-H2O
b5-NH3
y5
y6
b7-H2O
b7-NH3
y7
y8
y9
b11
y11
y13
500
1000
1500
EGN PG P L GP IG p PGV R bMax
RV G Pp G I P G LP GP NGE yMax
Intensity (%)
m/z
500
1000
1500

9

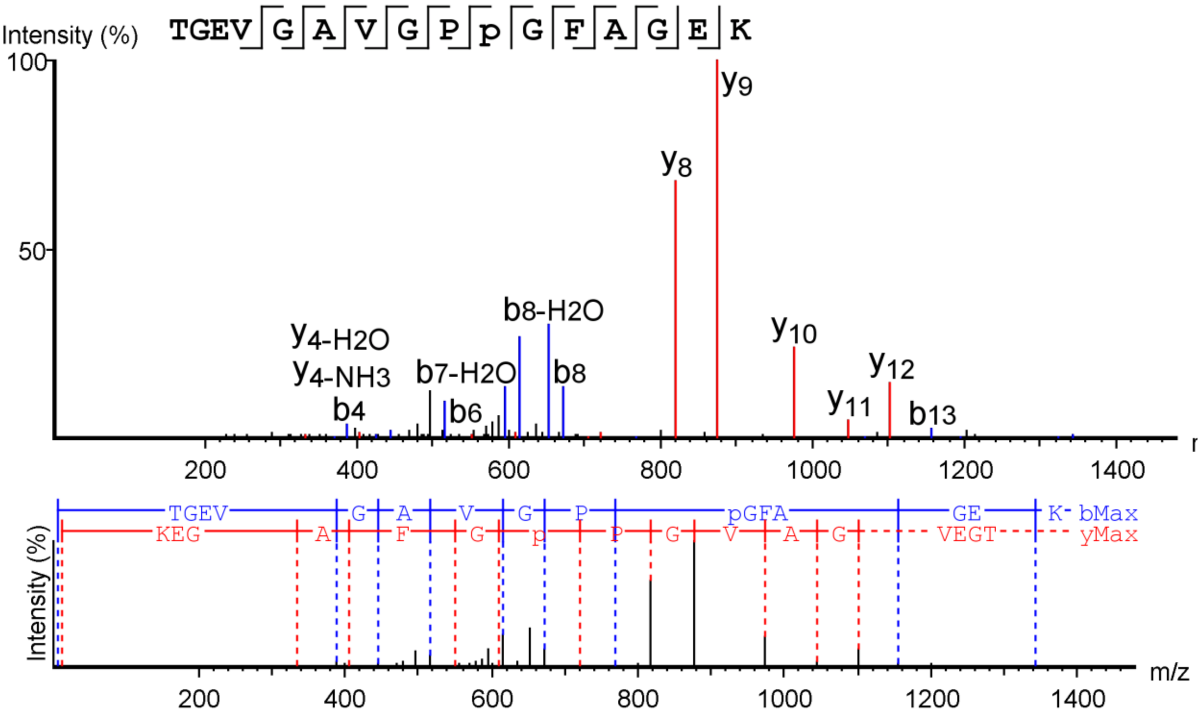
Intensity (%)
TGEV G A V G P p G F A G E K
100
50
y9
y8
b8-H2O
y10
y4-H2O
y4-NH3
b7-H2O
b8
y12
b4
b6
y11
b13
200
400
600
800
1000
1200
1400
TGEV G A V G P pGFA GE K bMax
KEG A F G p P G V A G VEGT yMax
Intensity (%)
m/z

10

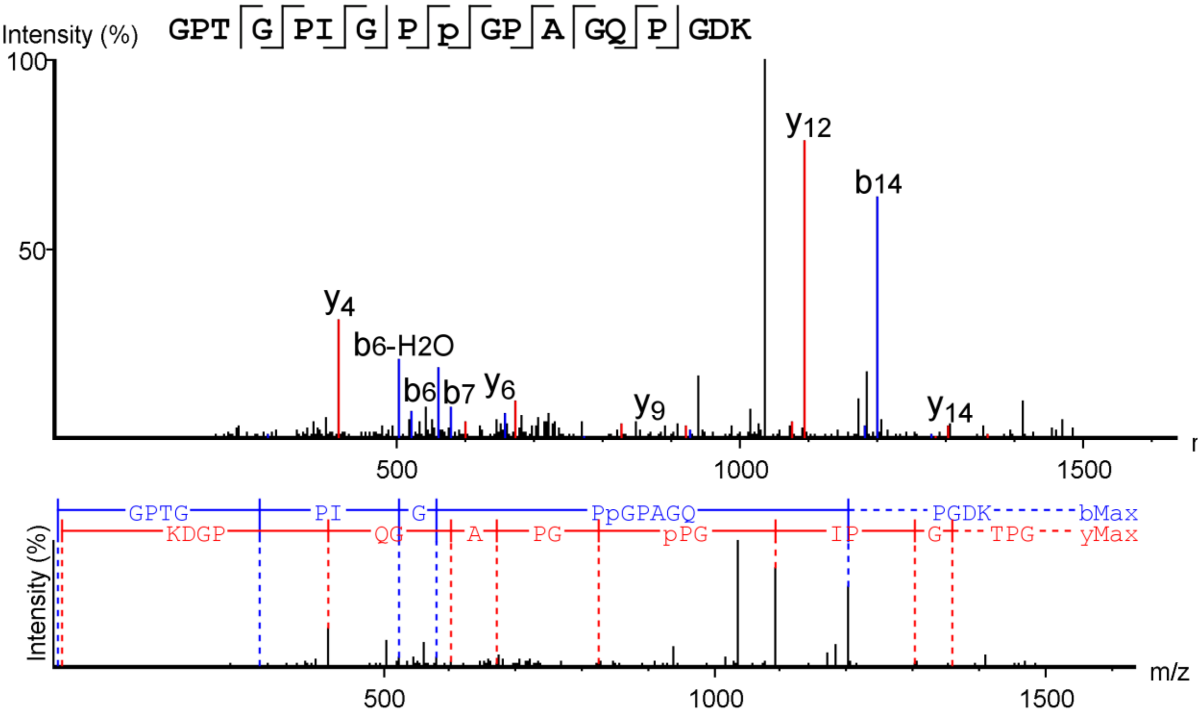
Intensity (%)
GPT G PI G P p GP A GQ P GDK
100
50
y12
b14
y4
b6-H2O
b6
b7
y6
y9
y14
500
1000
1500
GPTG PI G PpGPAGQ PGDK bMax
KDGP QG A PG pPG IP G TPG yMax
Intensity (%)
m/z

11

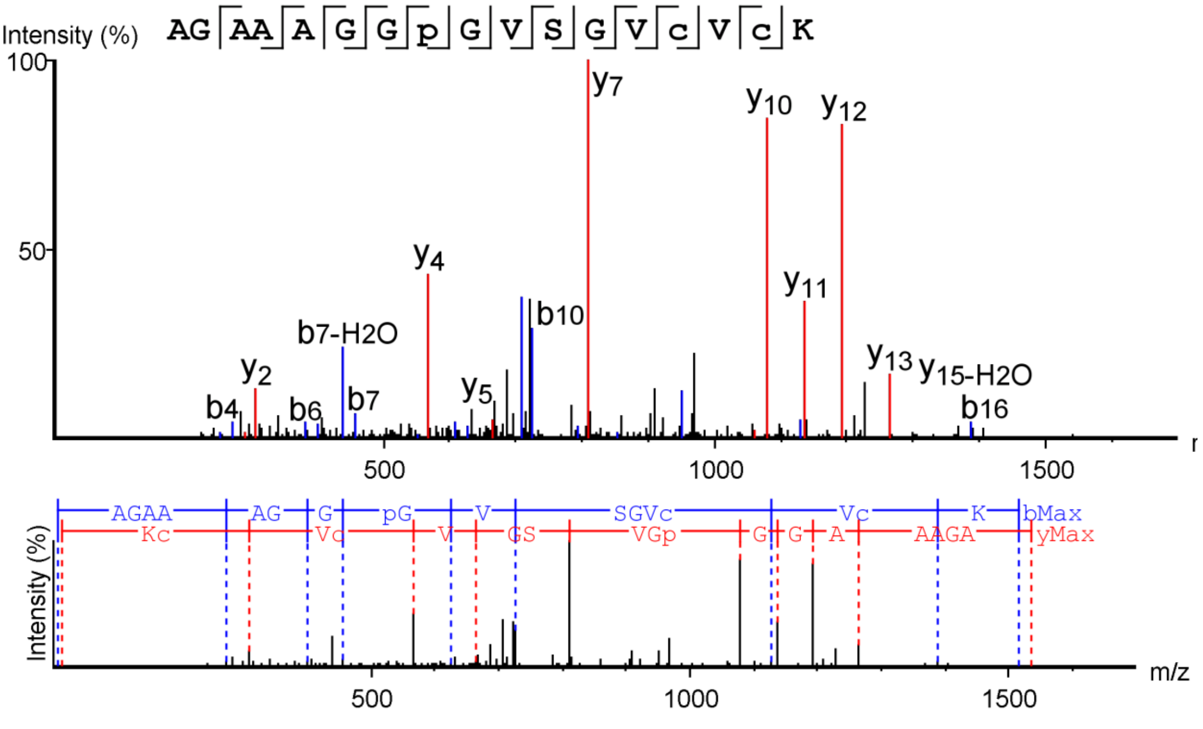
Intensity (%)
AG AA A G G p G V S G V c V c K
y7
y10
y12
y4
y11
b10
b7-H2O
y2
y13
y15-H2O
b4
b6
b7
y5
b16
100
50
500
1000
1500
AGAA AG G pG V SGVc Vc K bMax
Kc Vc V GS VGp G G A AAGA yMax
Intensity (%)
m/z

12

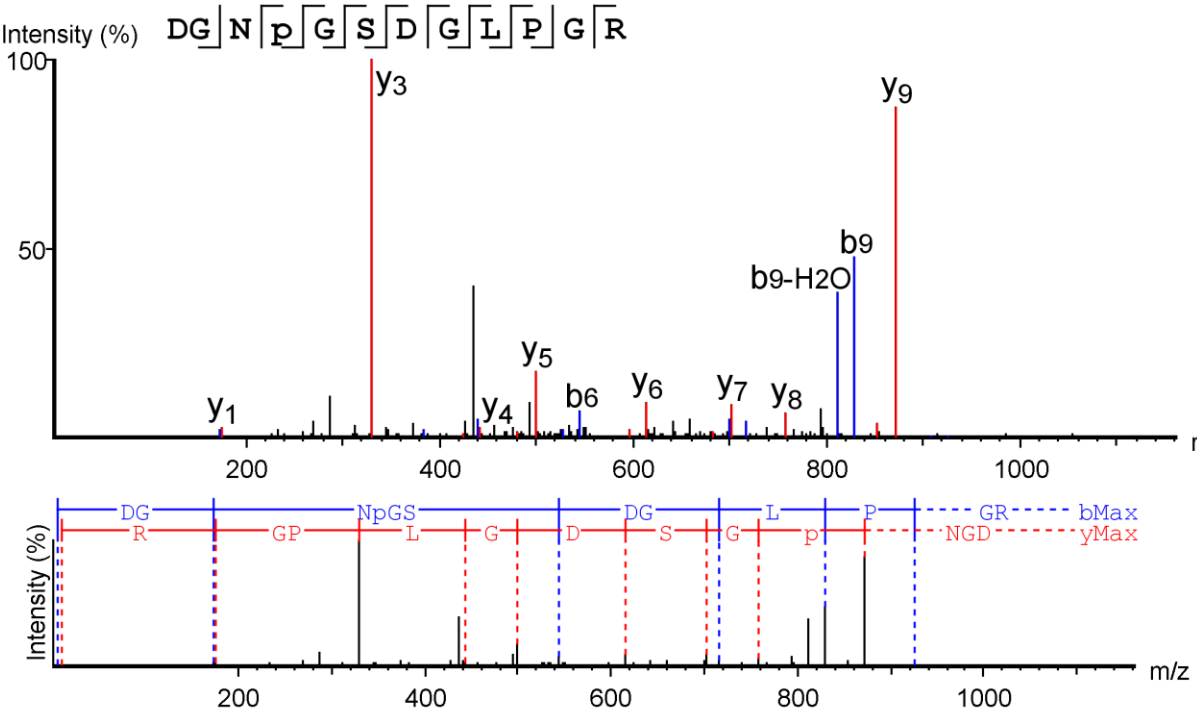
Intensity (%)
DG N p G S D G L P G R
y3
y9
b9
b9-H2O
y5
y1
y4
b6
y6
y7
y8
100
50
200
400
600
800
1000
DG NpGS DG L P GR bMax
R GP L G D S G p NGD yMax
Intensity (%)
m/z

13

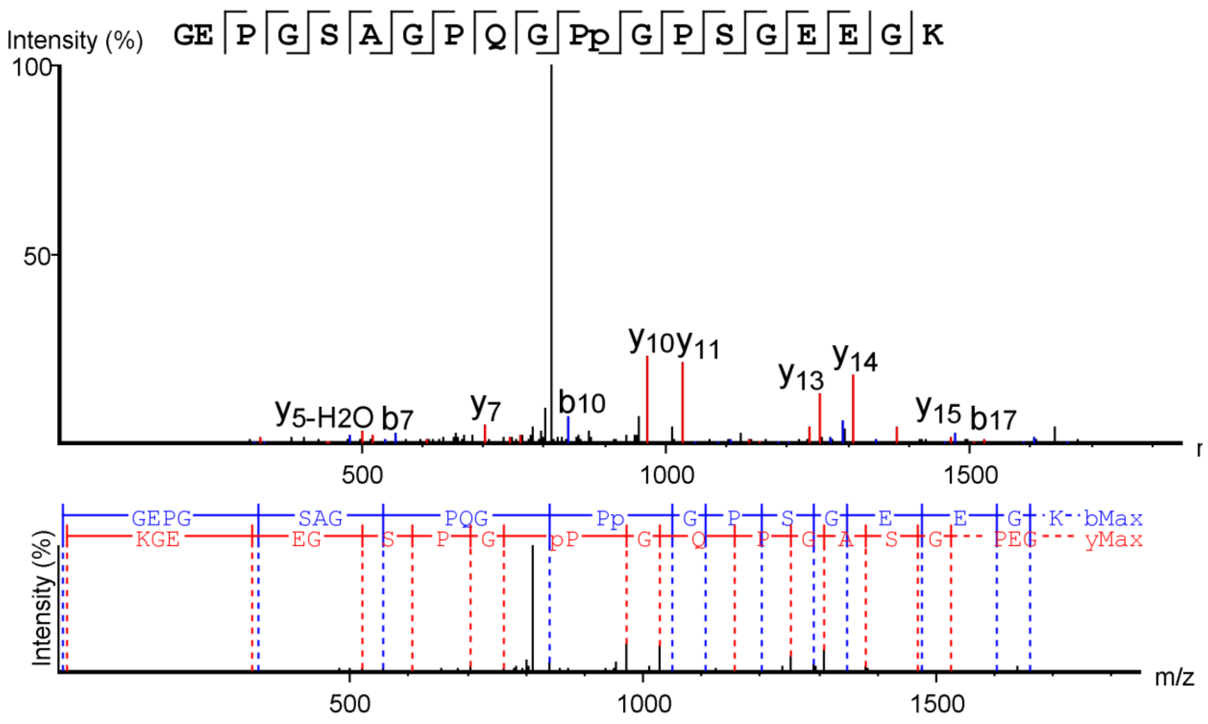
Intensity (%)
GE P G S A G P Q G Pp G P S G E E G K
y10 y11
y13 y14
y5-H2O b7
y7
b10
y15 b17
GEPG SAG PQG Pp G P S G E E G K bMax
KGE EG S P G pP G Q P G A S G PEG yMax
m/z

14

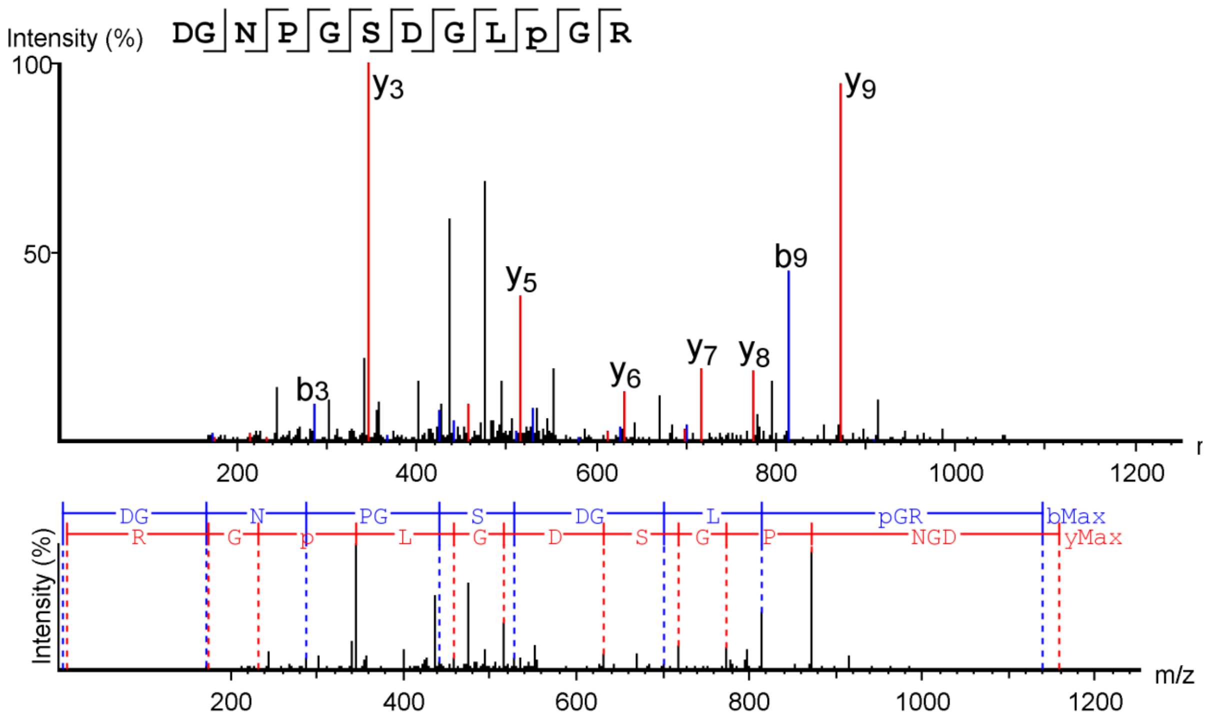
Intensity (%)
DG N P G S D G L p G R
y3
y9
y5
b9
b3
y6
y7
y8
DG N PG S DG L pGR bMax
R G p L G D S G P NGD yMax
m/z

15

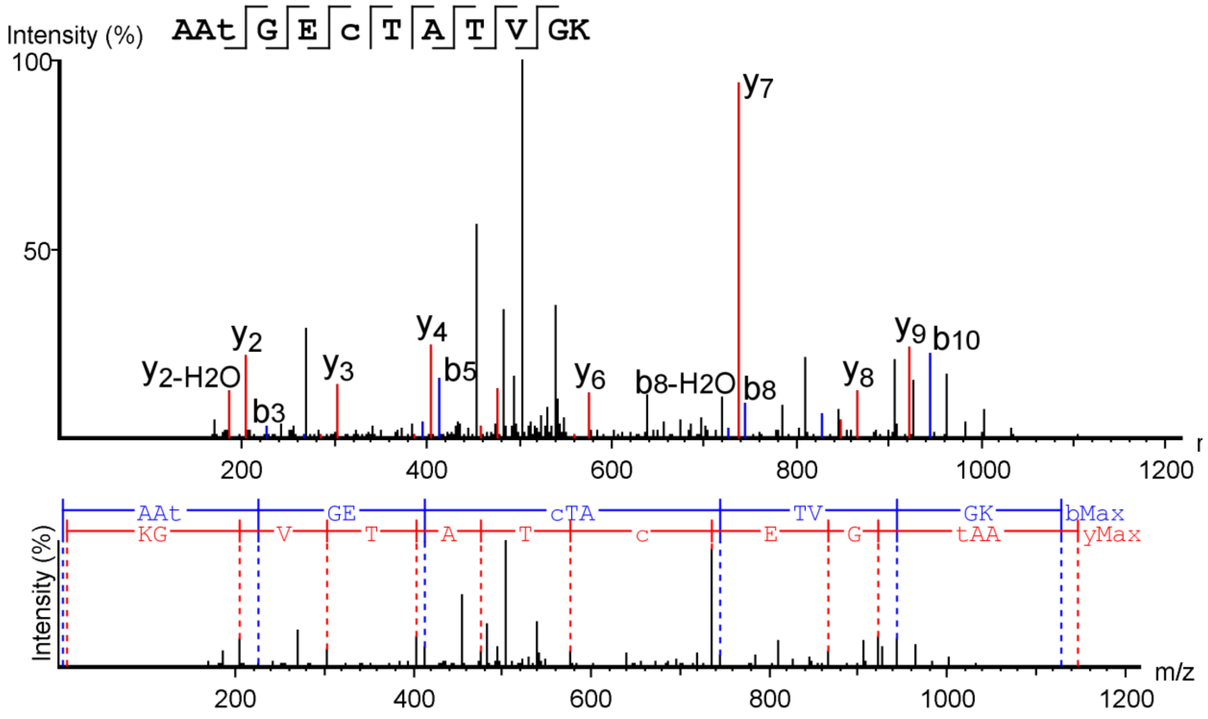
Intensity (%)
AAt G E c T A T V GK
100
50
y7
y2
y2-H2O
b3
y3
y4
b5
y6
b8-H2O
b8
y8
y9
b10
200
400
600
800
1000
1200
AAt GE cTA TV GK bMax
KG V T A T c E G tAA yMax
Intensity (%)
m/z

16

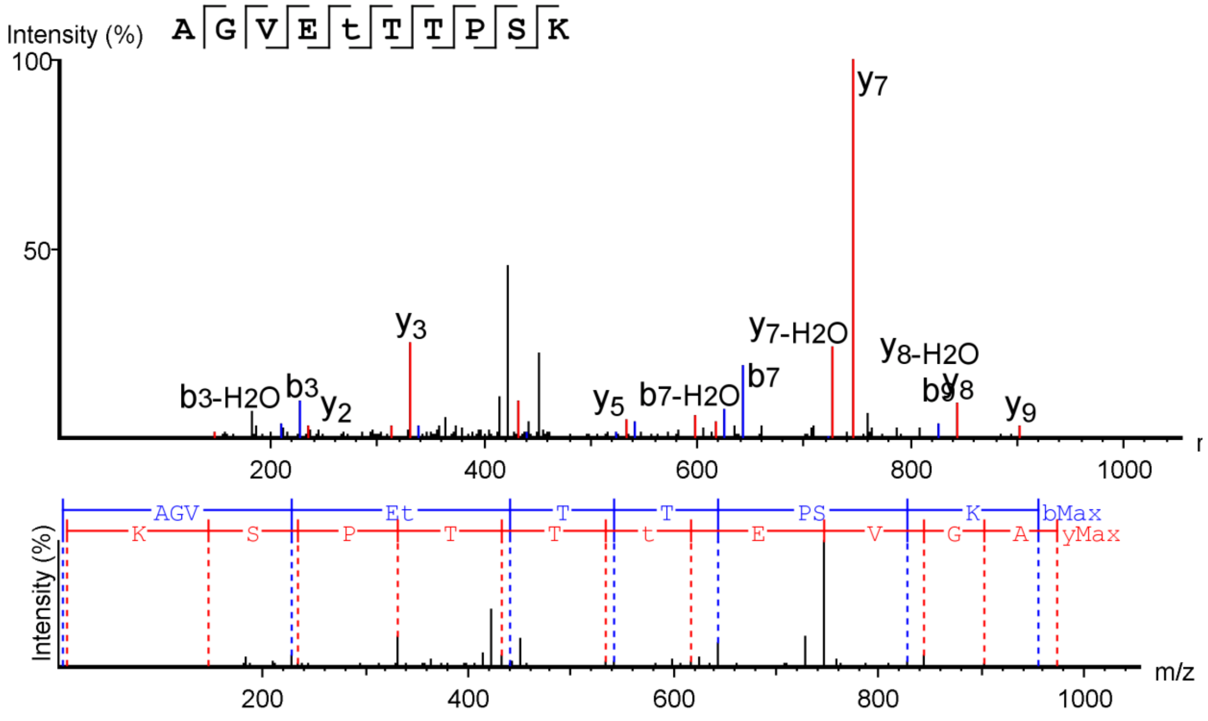
Intensity (%)
A G V E t T T P S K
100
50
y7
y3
b3-H2O
b3
y2
y5
b7-H2O
b7
y7-H2O
y8-H2O
b9
y8
y9
200
400
600
800
1000
AGV Et T T PS K bMax
K S P T T t E V G A yMax
Intensity (%)
m/z

17

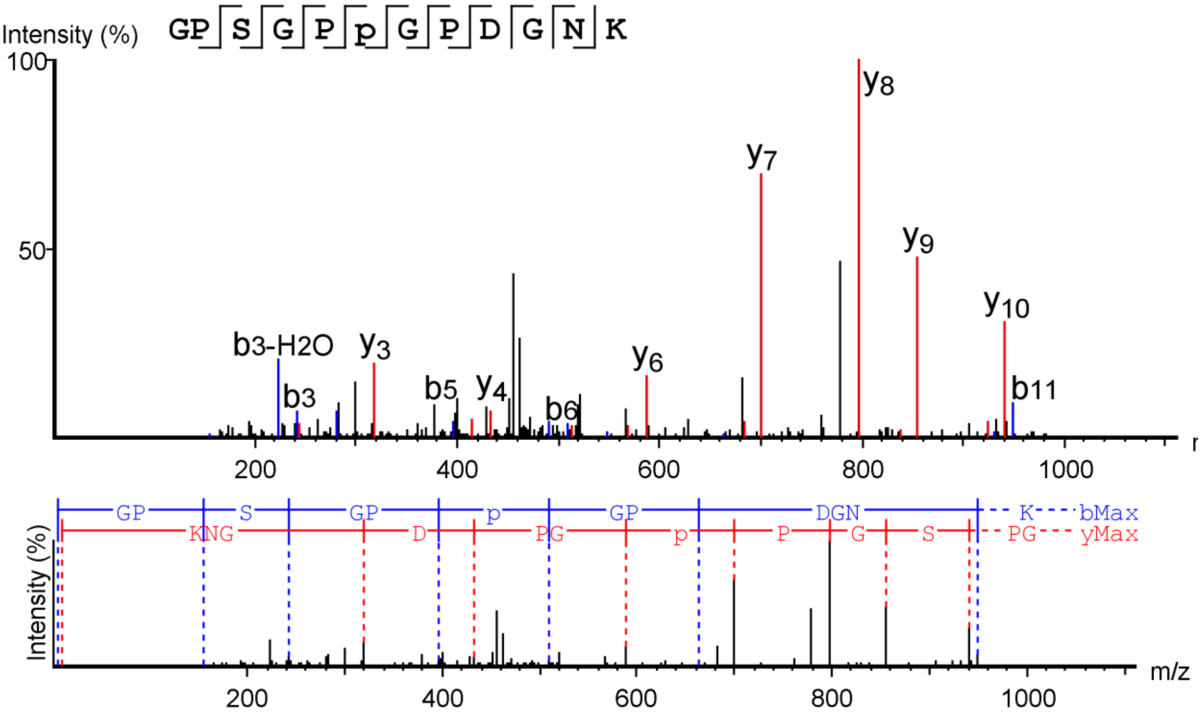
Intensity (%)
GP S G P p G P D G N K
100
50
b3-H2O
b3
y3
b5
y4
b6
y6
y7
y8
y9
y10
b11
200
400
600
800
1000
GP S GP p GP DGN K bMax
KNG D PG p P G S PG yMax
Intensity (%)
m/z

18

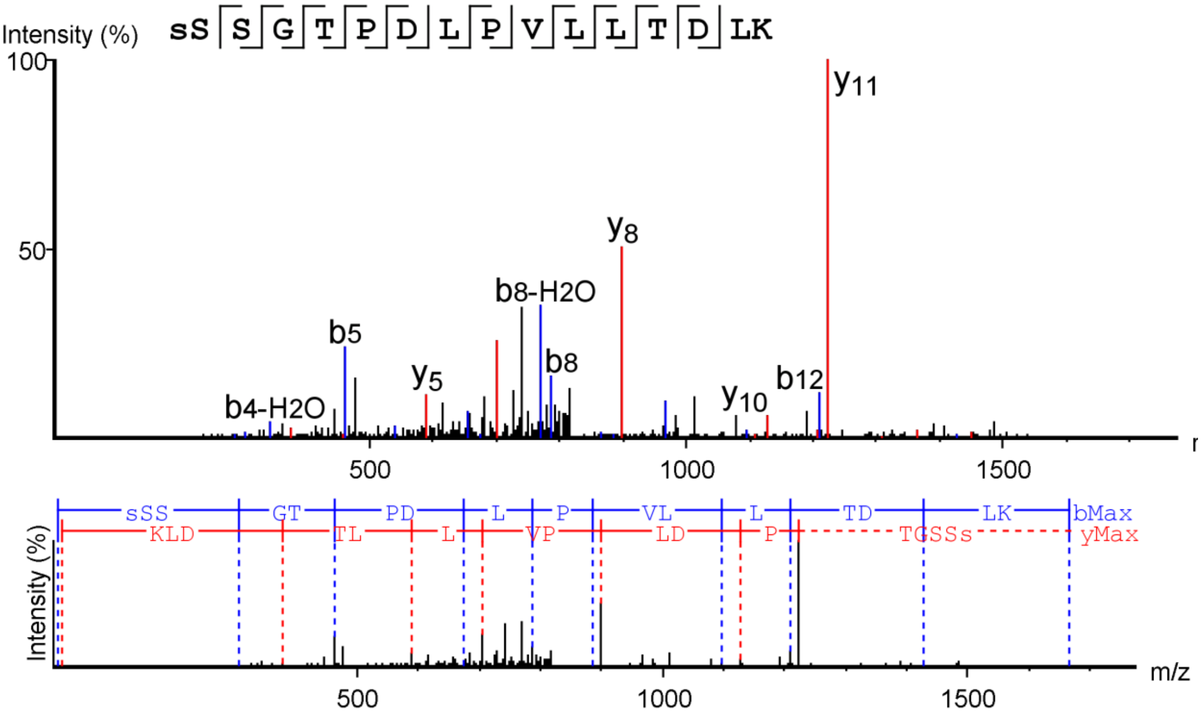
Intensity (%)
sS S G T P D L P V L L T D LK
100
50
b4-H2O
b5
y5
b8-H2O
b8
y8
y10
b12
y11
500
1000
1500
sSS GT PD L P VL L TD LK bMax
KLD TL L VP LD P TGSSs yMax
Intensity (%)
m/z

19

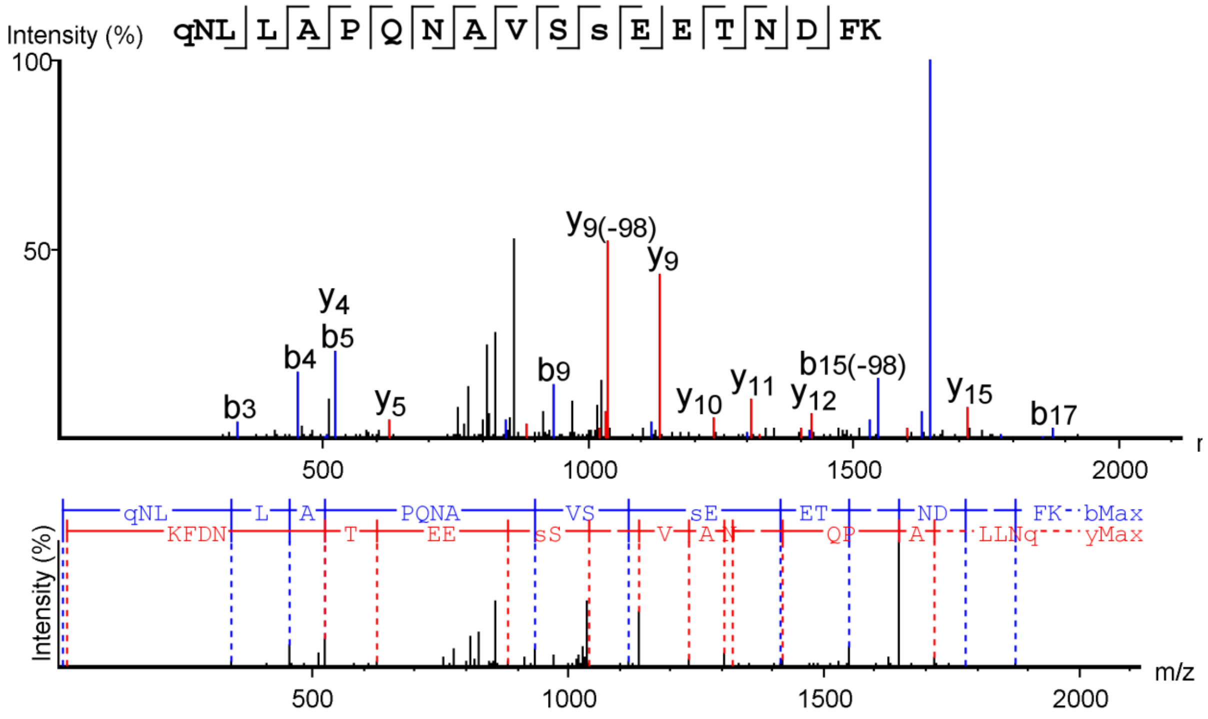

Intensity (%)
qNL L A P Q N A V S s E E T N D FK
y9(-98)
y9
y4
b5
b4
b3
y5
b9
y10
y11
y12
b15(-98)
y15
b17
m/z

20

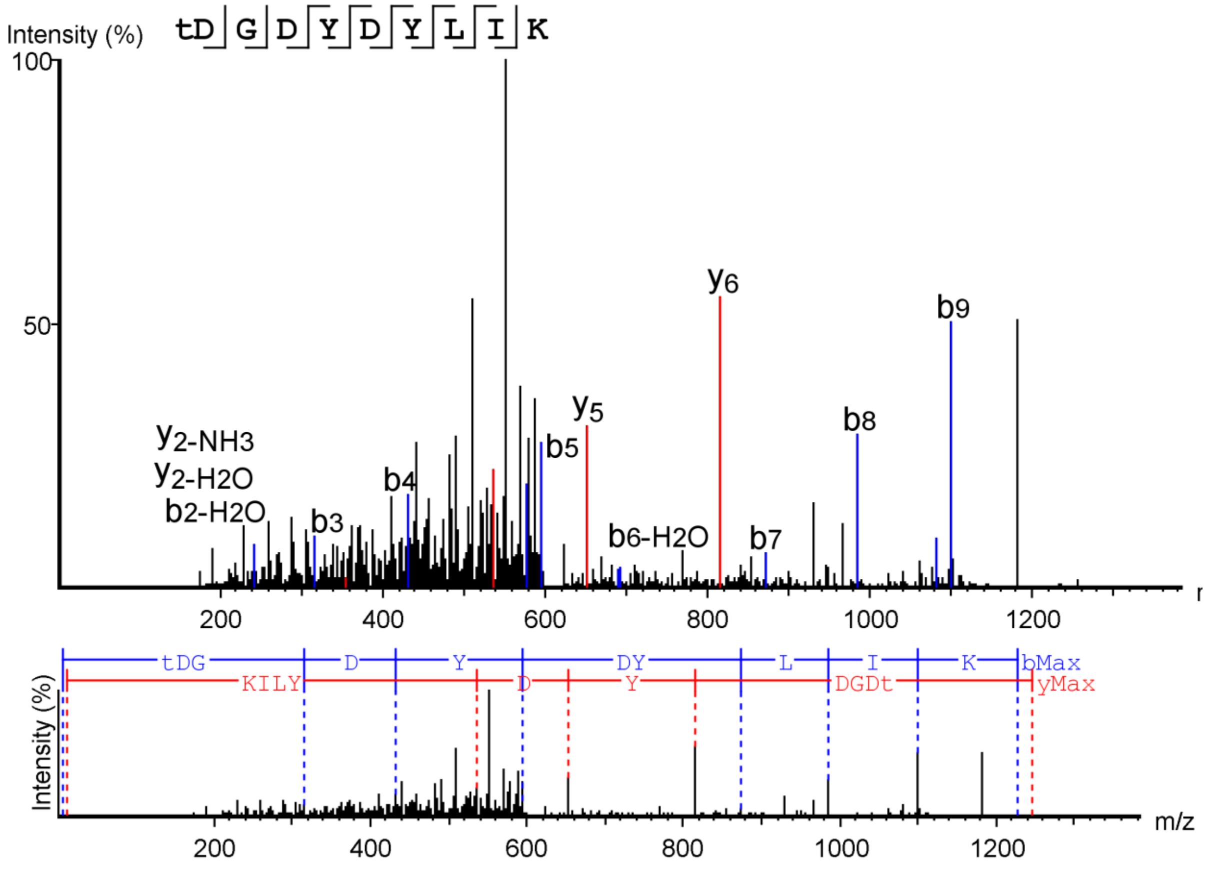

Intensity (%)
tD G D Y D Y L I K
y6
b9
y5
b5
b8
y2-NH3
y2-H2O
b2-H2O
b4
b3
b6-H2O
b7
m/z

21

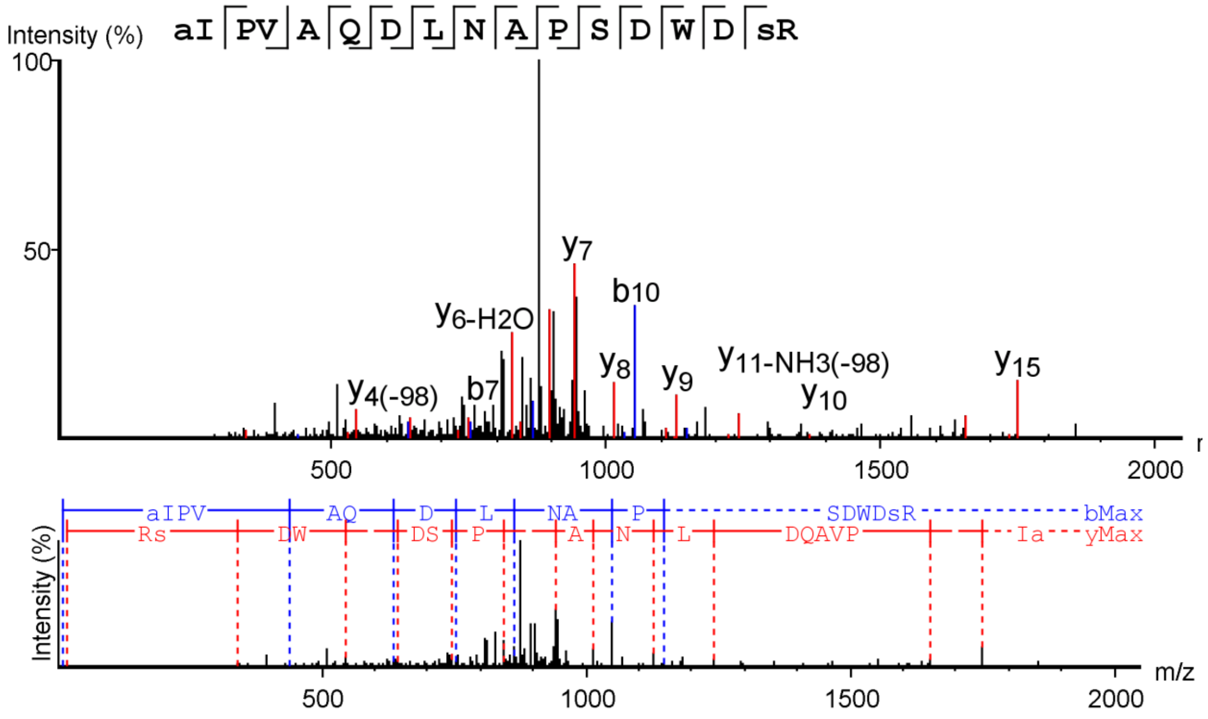

Intensity (%)
aI PV A Q D L N A P S D W D sR
100
50
y7
b10
y6-H2O
y8
y9
y11-NH3(-98)
y15
y4(-98)
b7
y10
500
1000
1500
2000
aIPV
AQ
D
L
NA
P
SDWDsR
bMax
Rs
DW
DS
P
A
N
L
DQAVP
Ia
yMax
Intensity (%)
m/z

22

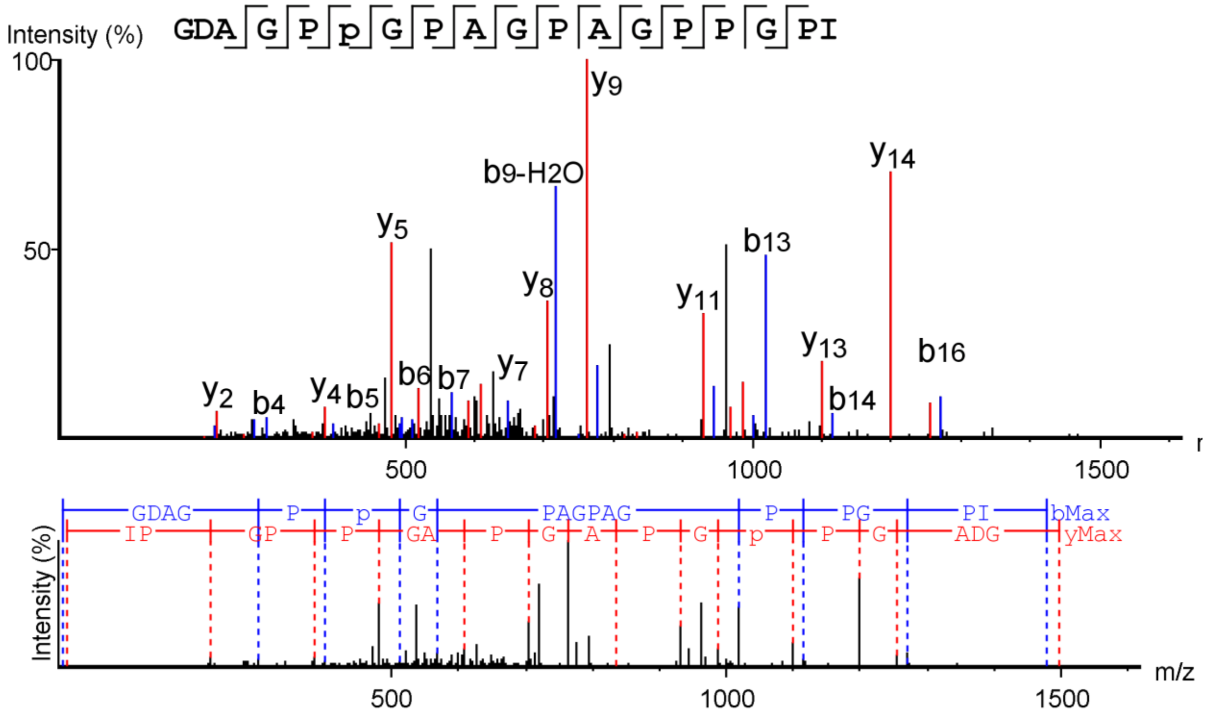

Intensity (%)
GDA G P p G P A G P A G P P G PI
100
50
y9
b9-H2O
y14
y5
b13
y8
y11
y13
b16
y2
b4
y4
b5
b6
b7
y7
b14
500
1000
1500
GDAG
P
p
G
PAGPAG
P
PG
PI
bMax
IP
GP
P
GA
P
G
A
P
G
p
P
G
ADG
yMax
Intensity (%)
m/z

23

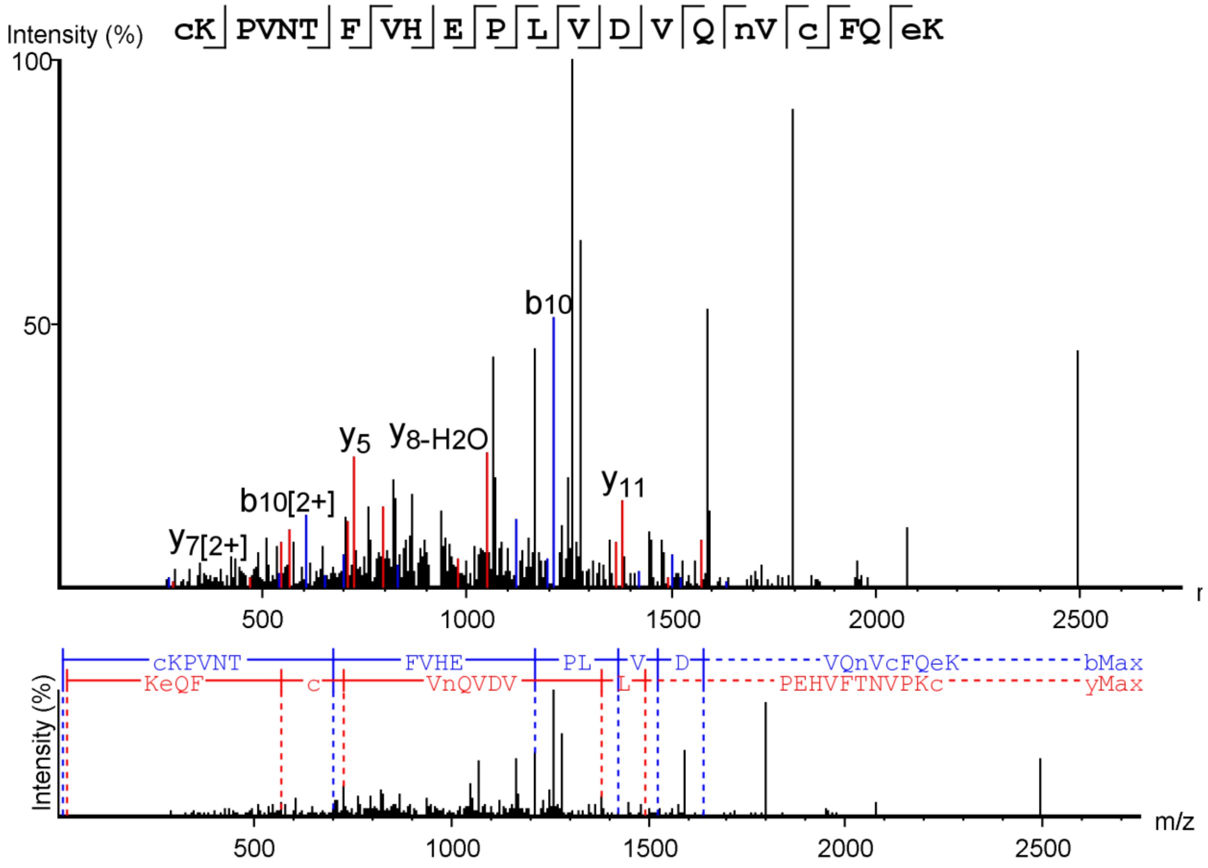

Intensity (%)
cK PVNT F VH E P L V D V Q nV c FQ eK
b10
y5
y8-H2O
y11
b10[2+]
y7[2+]
cKPVNT
FVHE
PL
V
D
VQnVcFQeK
bMax
KeQF
c
VnQVDV
L
PEHVFTNVPKc
yMax
m/z

24

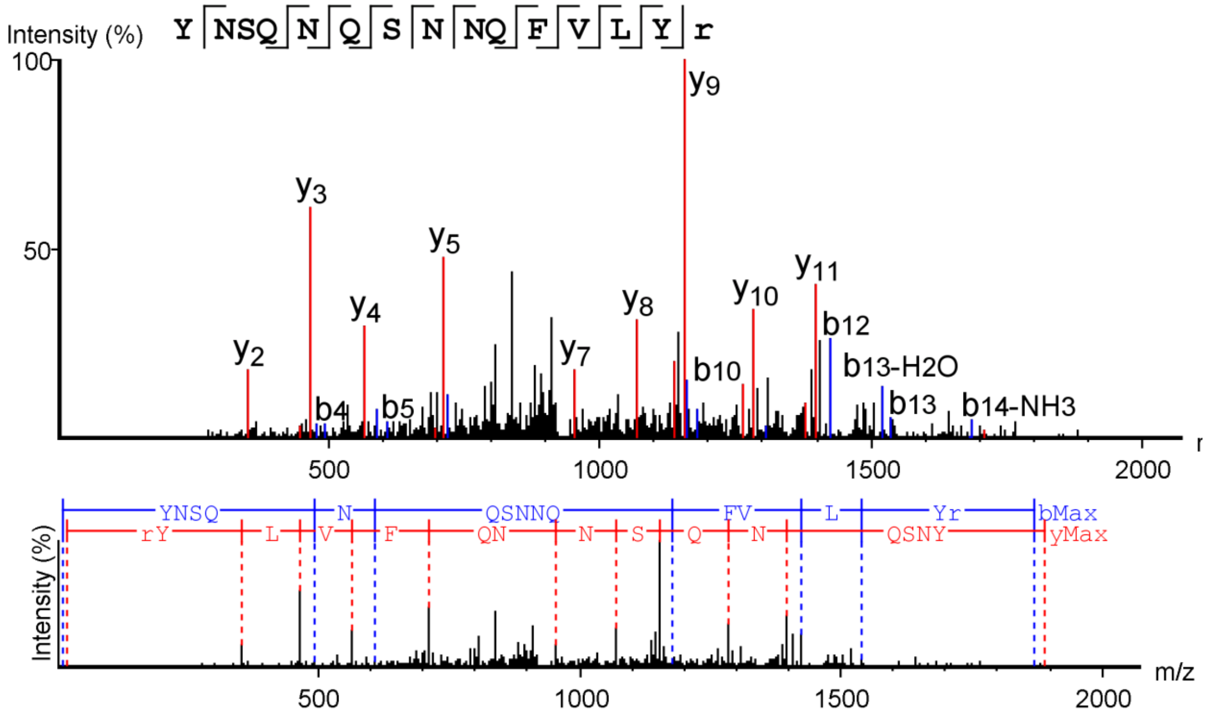

Intensity (%)
Y NSQ N Q S N NQ F V L Y r
y9
y3
y5
y4
y8
y10
y11
b12
y2
y7
b10
b13-H2O
b4
b5
b13
b14-NH3
YNSQ
N
QSNNQ
FV
L
Yr
bMax
rY
L
V
F
QN
N
S
Q
N
QSNY
yMax
m/z

25

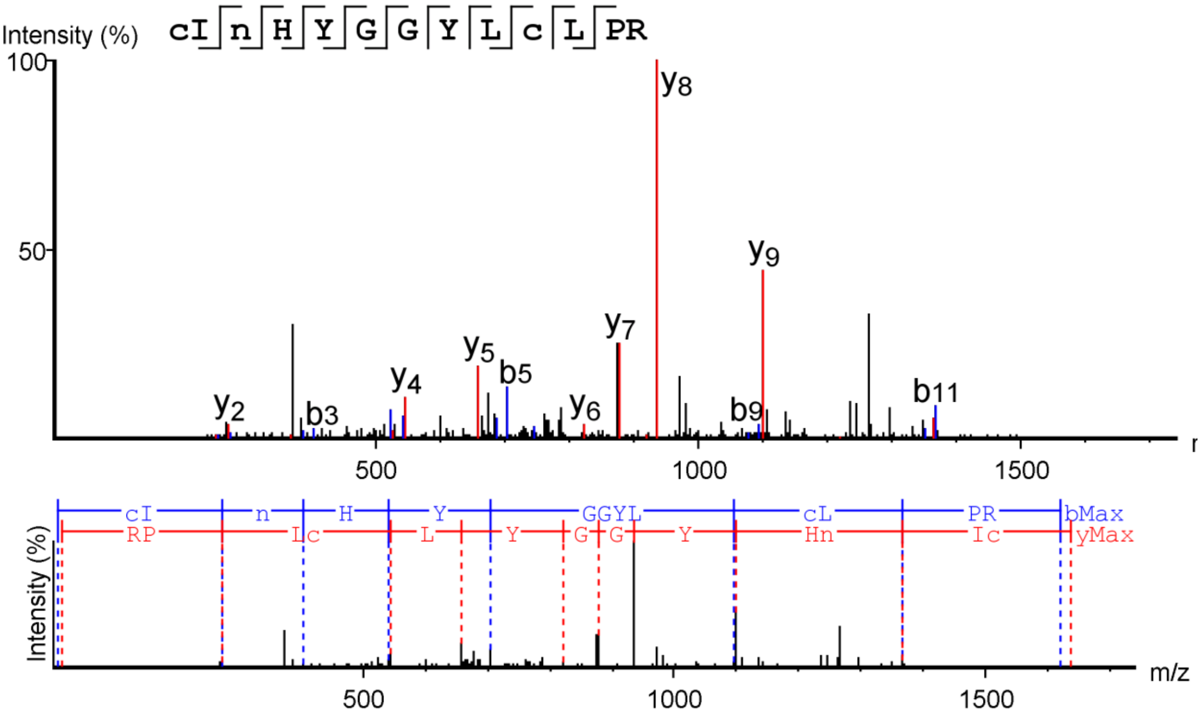
Intensity (%)
cI n H Y G G Y L c L PR
100
50
y8
y9
y7
y5
b5
y4
y2
b3
y6
b9
b11
500
1000
1500
cI n H Y GGYL cL PR bMax
RP Ic L Y G G Y Hn Ic yMax
Intensity (%)
500
1000
1500
m/z

26

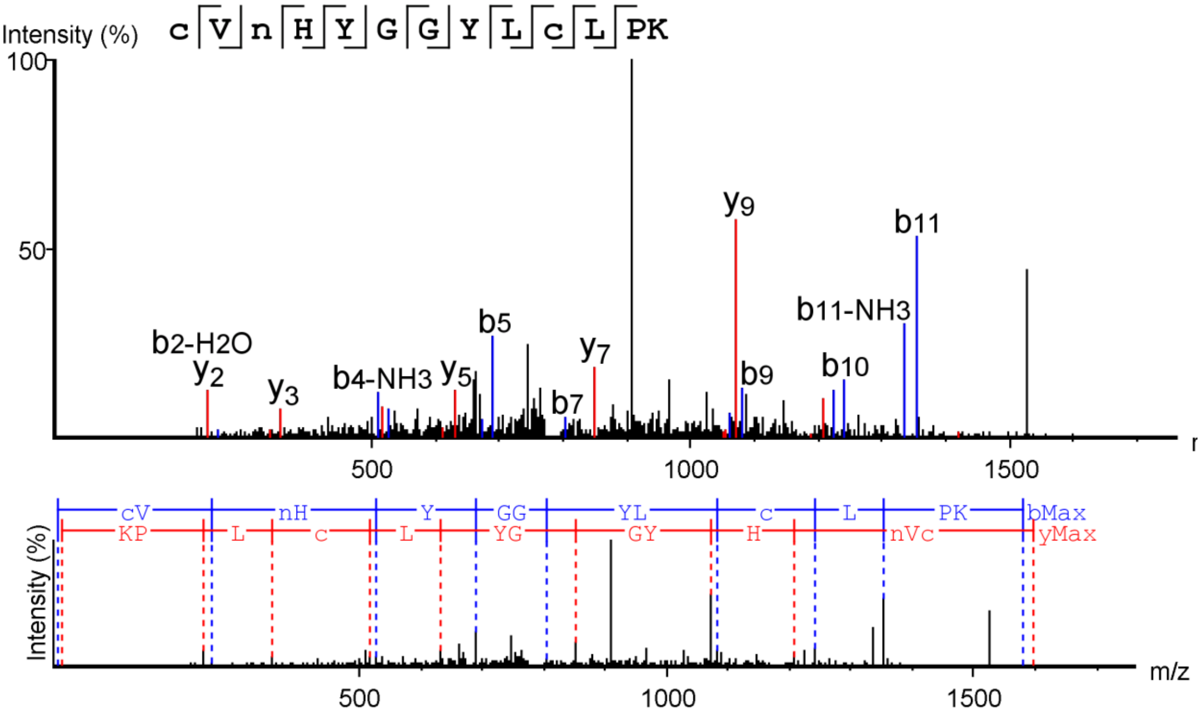
Intensity (%)
c V n H Y G G Y L c L PK
100
50
y9
b11
b11-NH3
b2-H2O
y2
y3
b4-NH3
y5
b5
y7
b7
b9
b10
500
1000
1500
cV nH Y GG YL c L PK bMax
KP L c L YG GY H nVc yMax
Intensity (%)
500
1000
1500
m/z

27

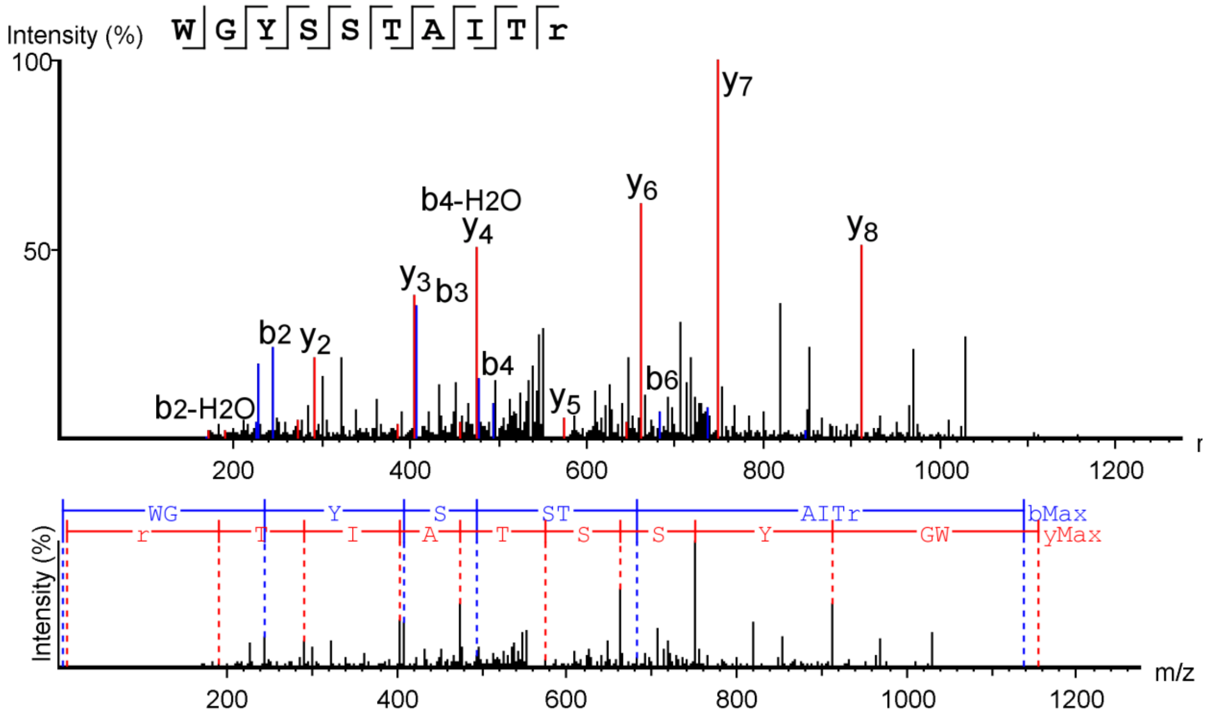
Intensity (%)
W G Y S S T A I T r
y7
y6
b4-H2O
y4
y8
y3
b3
b2
y2
b4
b6
y5
b2-H2O
WG Y S ST AITr bMax
r T I A T S S Y GW yMax
Intensity (%)
m/z

28

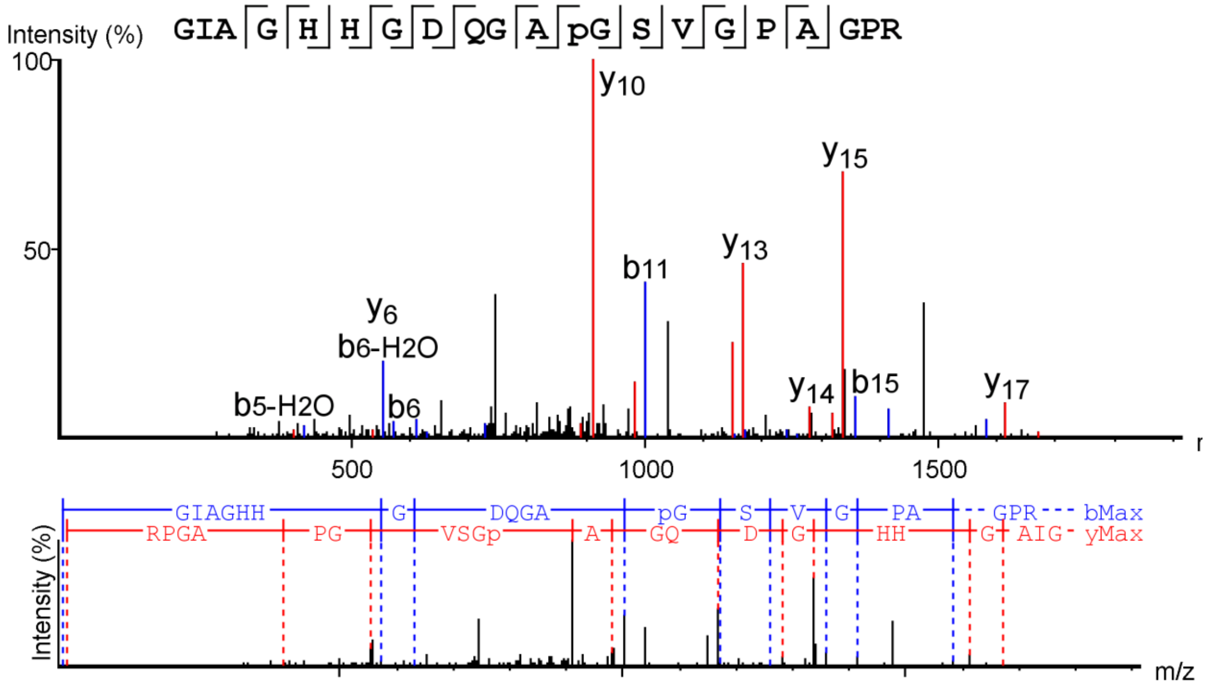
Intensity (%)
GIA G H H G D QG A pG S V G P A GPR
y10
y15
b11
y13
y6
b6-H2O
b5-H2O
b6
y14
b15
y17
GIAGHH G DQGA pG S V G PA GPR bMax
RPGA PG VSGp A GQ D G HH G AIG yMax
Intensity (%)
m/z

29

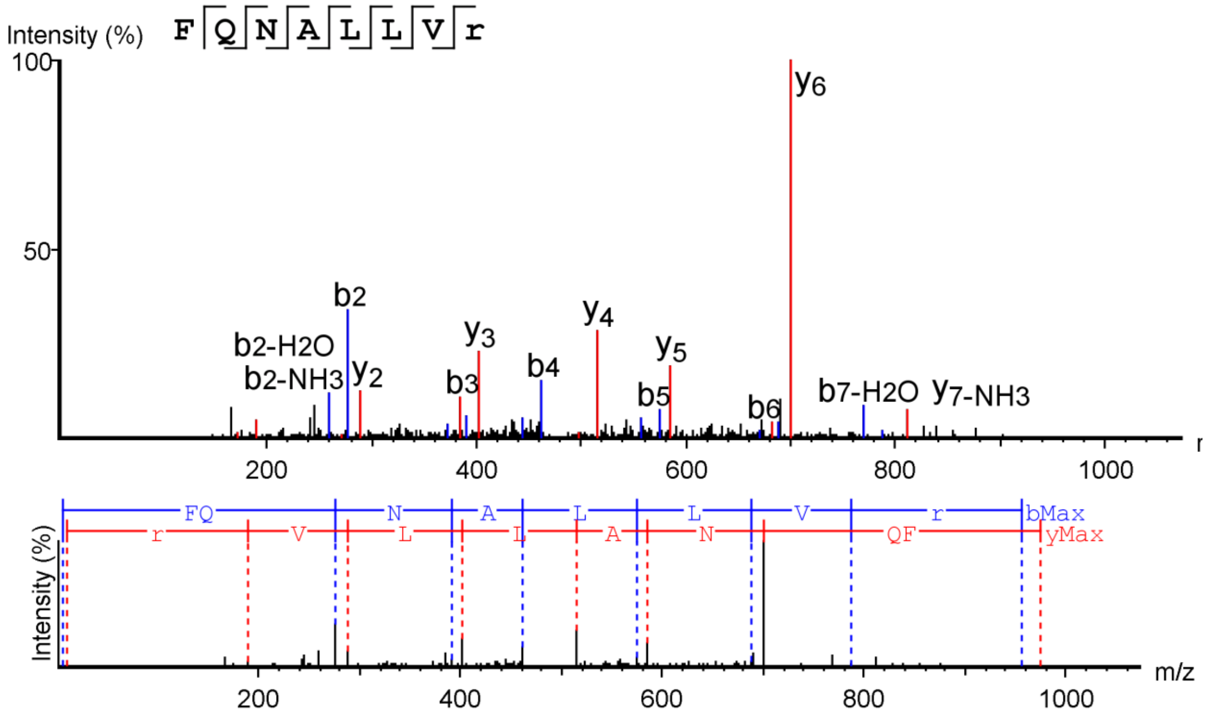
Intensity (%)
F Q N A L L V r
y6
b2
b2-H2O
b2-NH3
y2
y3
b3
y4
b4
y5
b5
b6
b7-H2O
y7-NH3
m/z
FQ N A L L V r bMax
r V L L A N QF yMax
Intensity (%)
m/z

30

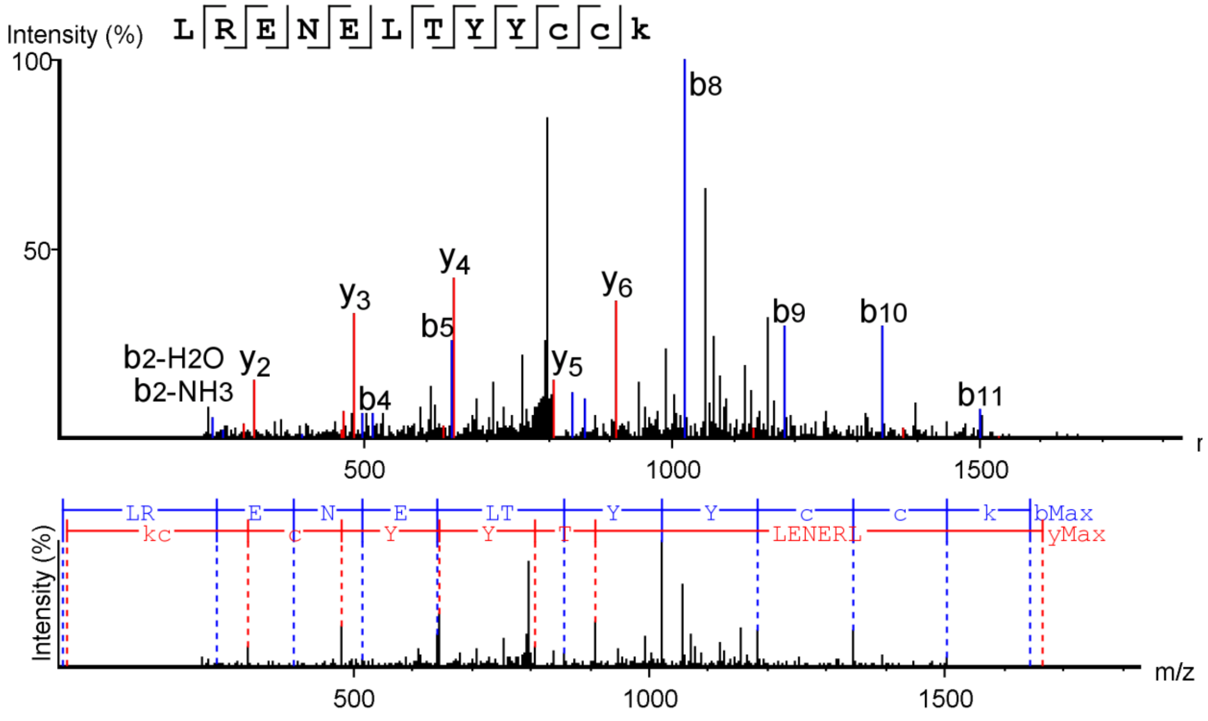
Intensity (%)
L R E N E L T Y Y c c k
b8
y4
y3
y6
b5
b9
b10
y5
b2-H2O
b2-NH3
y2
b4
b11
m/z
LR E N E LT Y Y c c k bMax
kc c Y Y T LENERL yMax
Intensity (%)
m/z

31

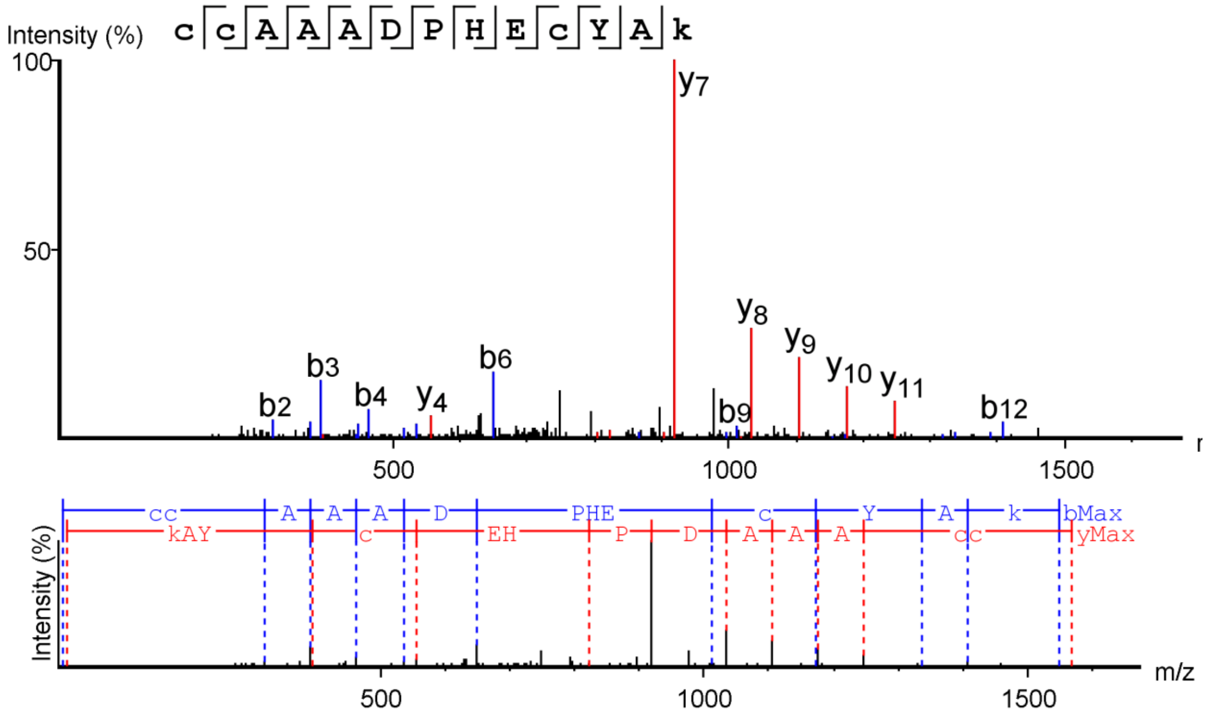
Intensity (%)
c c A A A D P H E c Y A k
y7
y8
y9
y10
y11
b2
b3
b4
y4
b6
b9
b12
cc A A A D PHE c Y A k bMax
kAY c EH P D A A A cc yMax
m/z

32

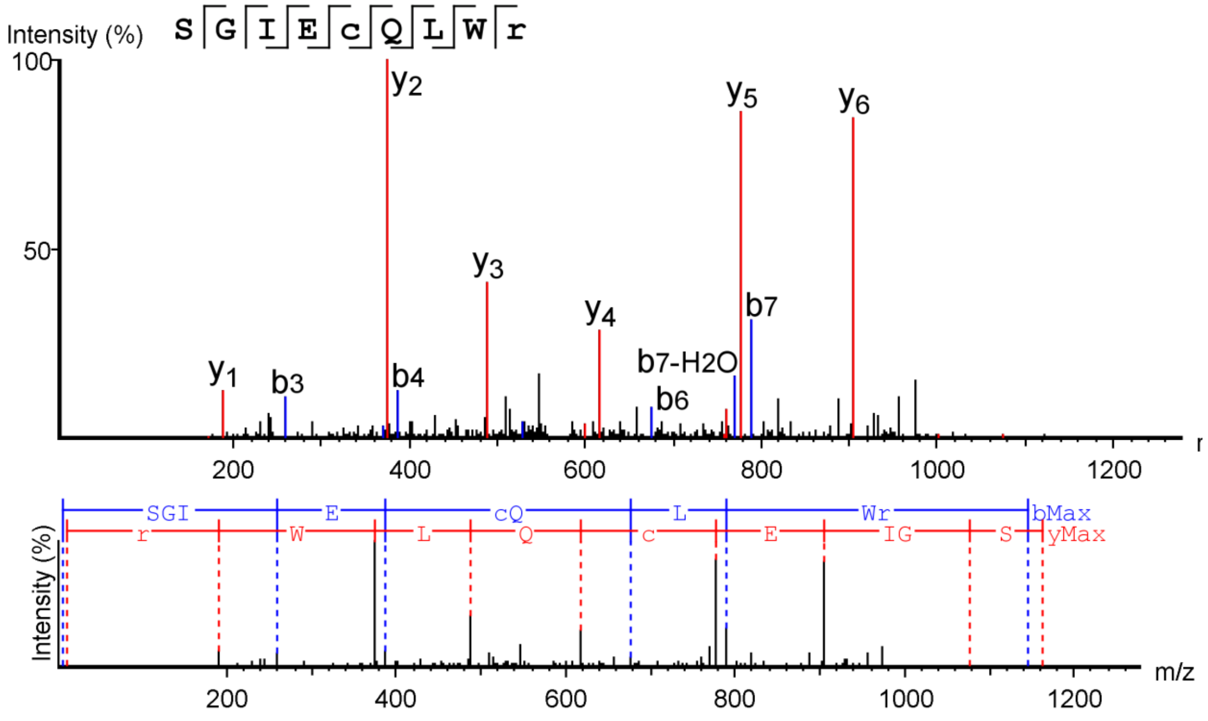
Intensity (%)
S G I E c Q L W r
y1
b3
y2
b4
y3
y4
b7-H2O
b6
y5
b7
y6
SGI E cQ L Wr bMax
r W L Q c E IG S yMax
m/z

33

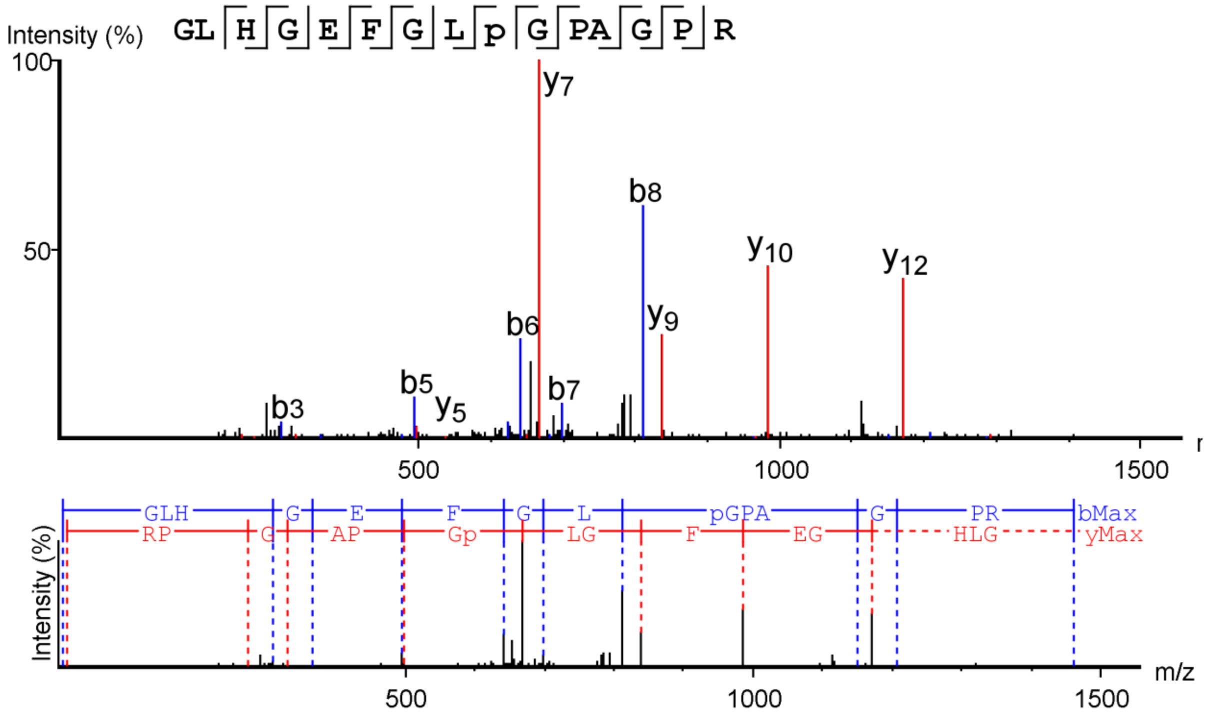
Intensity (%)
GL H G E F G L p G PA G P R
100
50
y7
b8
y10
y12
y9
b6
b5
y5
b7
b3
500
1000
1500
GLH G E F G L pGPA G PR bMax
RP G AP Gp LG F EG HLG yMax
Intensity (%)
m/z
500
1000
1500

34

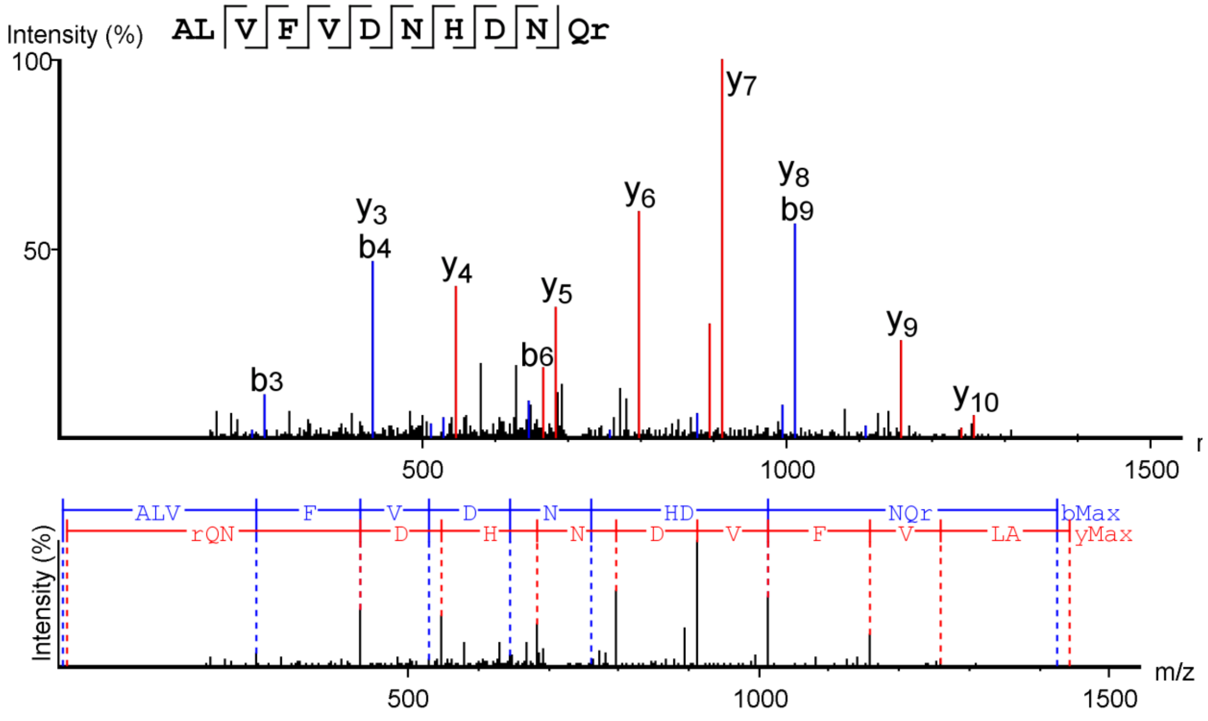
Intensity (%)
AL V F V D N H D N Qr
100
50
y7
y8
b9
y6
y3
b4
y4
y5
b6
b3
y9
y10
500
1000
1500
ALV F V D N HD NQr bMax
rQN D H N D V F V LA yMax
Intensity (%)
m/z
500
1000
1500

35

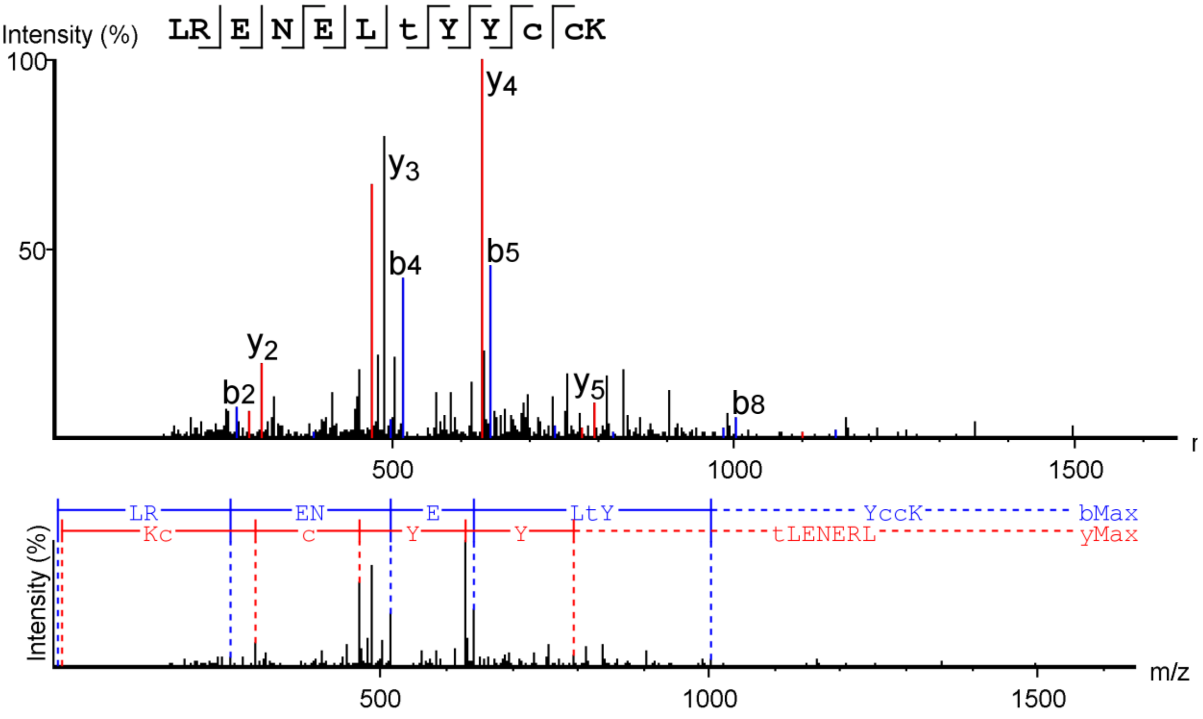

Intensity (%)
LR E N E L t Y Y c cK
100
50
y4
y3
b4
b5
y2
b2
y5
b8
500
1000
1500
LR EN E LtY YccK bMax
Kc c Y Y tLENERL yMax
Intensity (%)
m/z
500
1000
1500

36

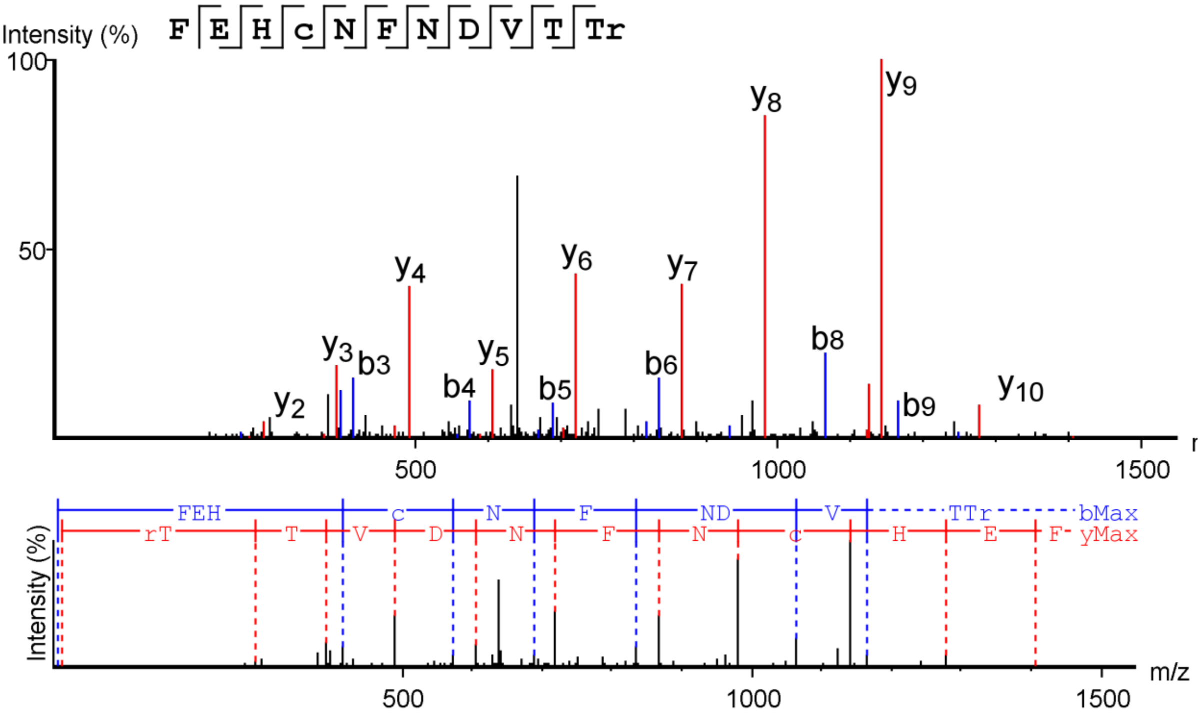

Intensity (%)
F E H c N F N D V T Tr
100
50
y8
y9
y4
y6
y7
y3
b3
y5
b8
b6
b4
b5
y2
b9
y10
500
1000
1500
FEH c N F ND V TTr bMax
rT T V D N F N c H E F yMax
Intensity (%)
m/z
500
1000
1500

37

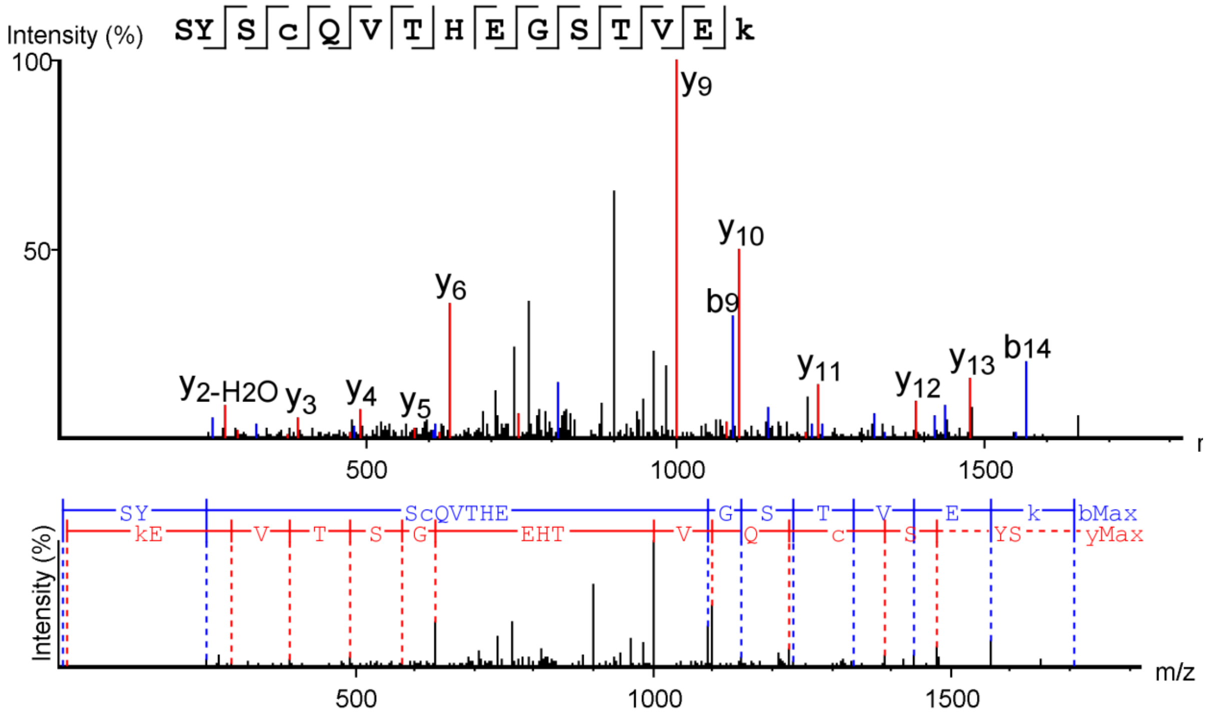
Intensity (%)
SY S c Q V T H E G S T V E k
100
50
y9
y10
b9
y6
y11
y12
y13
b14
y2-H2O
y3
y4
y5
500
1000
1500
SY
ScQVTHE
G
S
T
V
E
k
bMax
kE
V
T
S
G
EHT
V
Q
c
S
YS
yMax
Intensity (%)
m/z
500
1000
1500

38

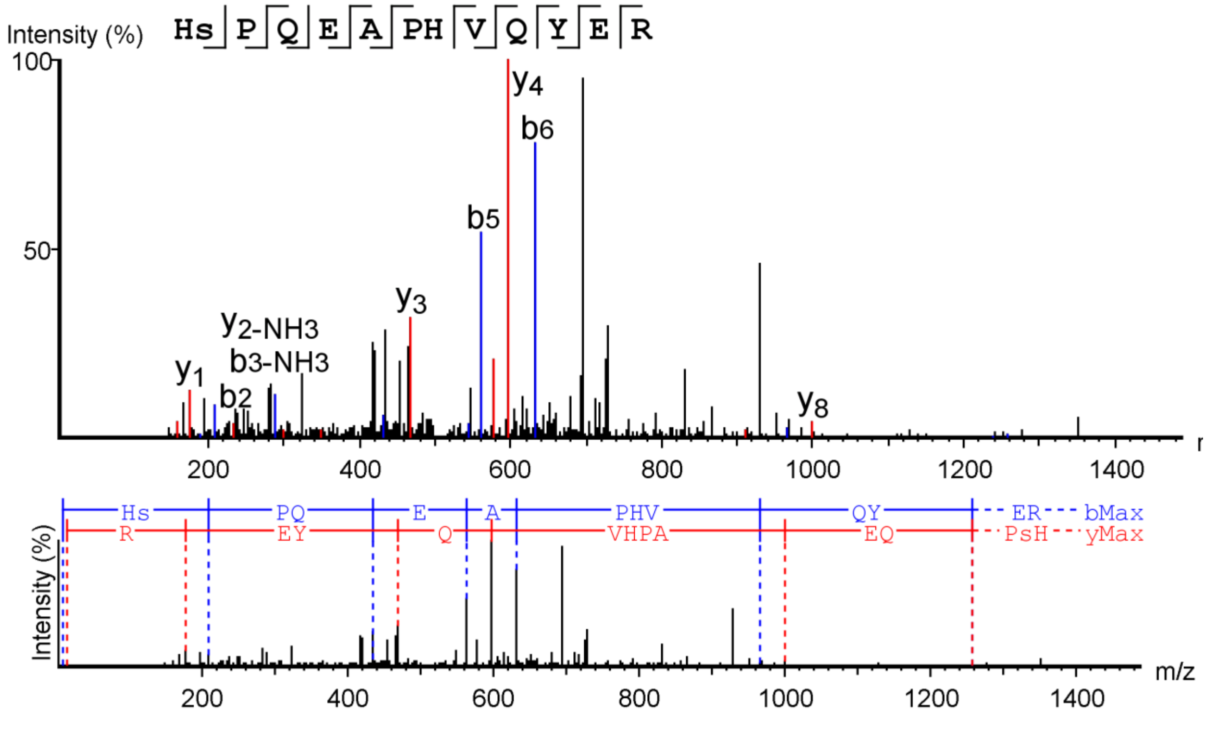
Intensity (%)
Hs P Q E A PH V Q Y E R
100
50
y4
b6
b5
y3
y2-NH3
b3-NH3
y1
b2
y8
200
400
600
800
1000
1200
1400
Hs
PQ
E
A
PHV
QY
ER
bMax
R
EY
Q
VHPA
EQ
PsH
yMax
Intensity (%)
m/z
200
400
600
800
1000
1200
1400

39

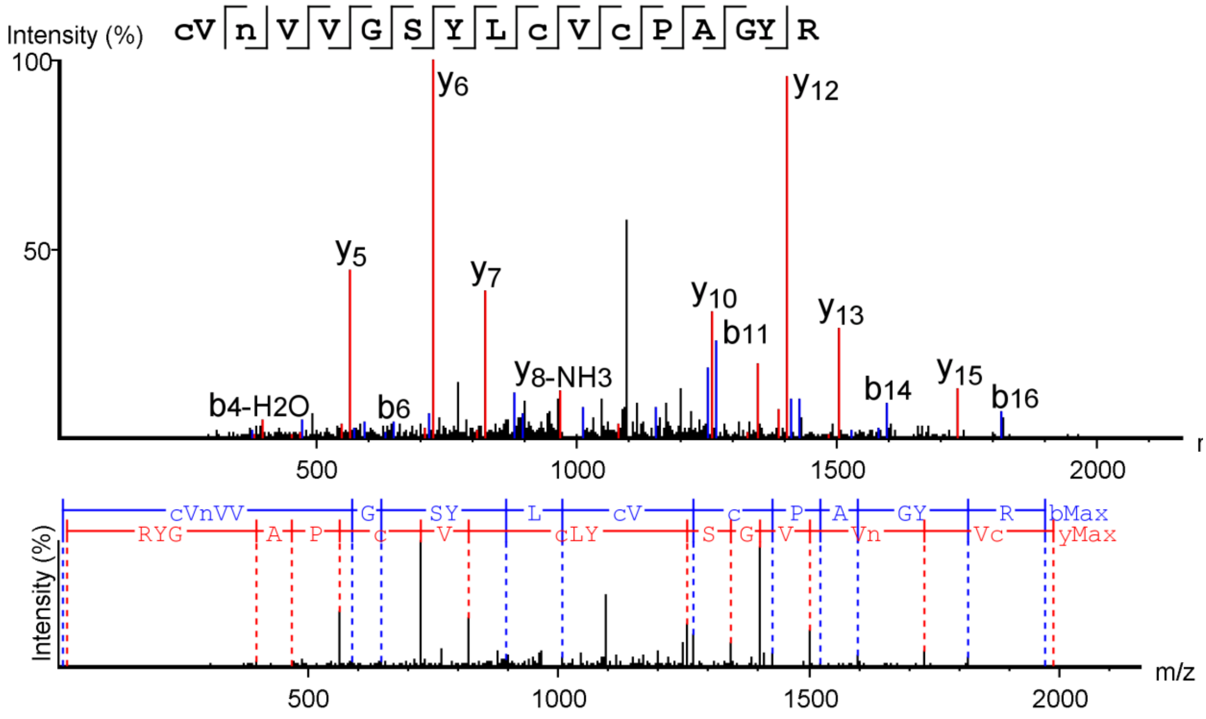
Intensity (%)
cV n V V G S Y L c V c P A GY R
y6
y12
y5
y7
y10
b11
y13
y8-NH3
b4-H2O
b6
b14
y15
b16
cVnVV G SY L cV c P A GY R bMax
RYG A P c V cLY S G V Vn Vc yMax
Intensity (%)
m/z

40

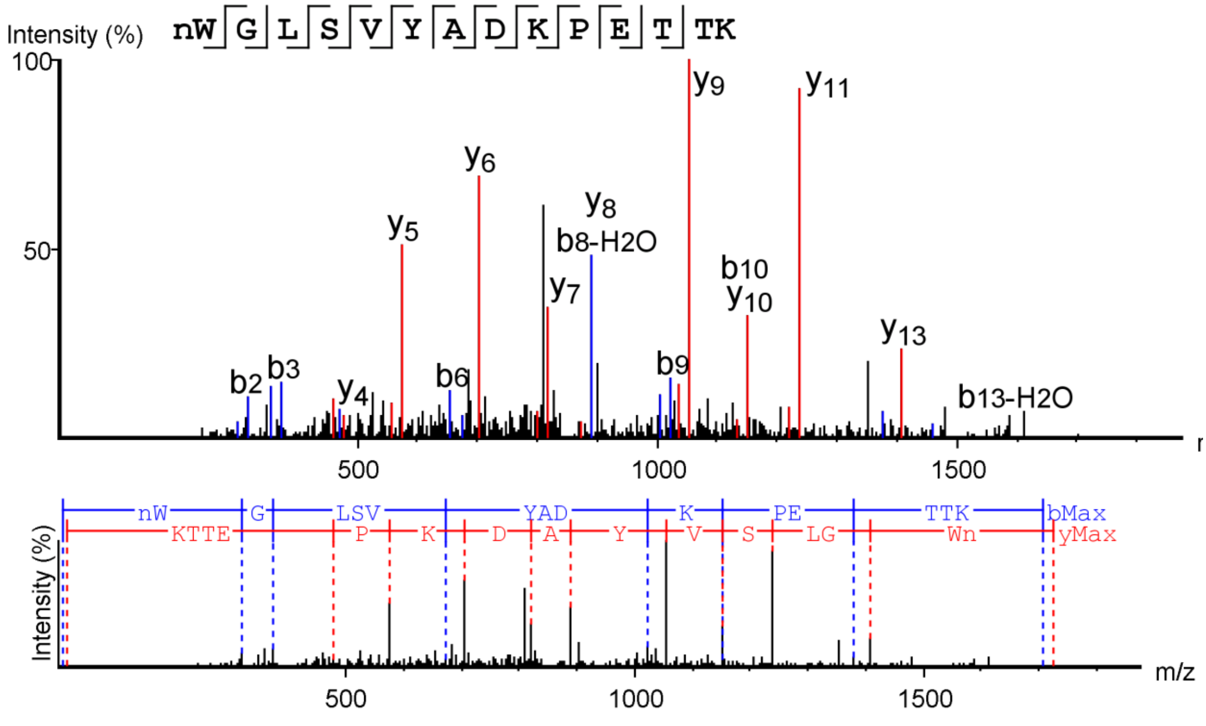
Intensity (%)
nW G L S V Y A D K P E T TK
y9
y11
y6
y8
b8-H2O
y5
y7
b10
y10
y13
b2
b3
y4
b6
b9
b13-H2O
nW G LSV YAD K PE TTK bMax
KTTE P K D A Y V S LG Wn yMax
Intensity (%)
m/z

41

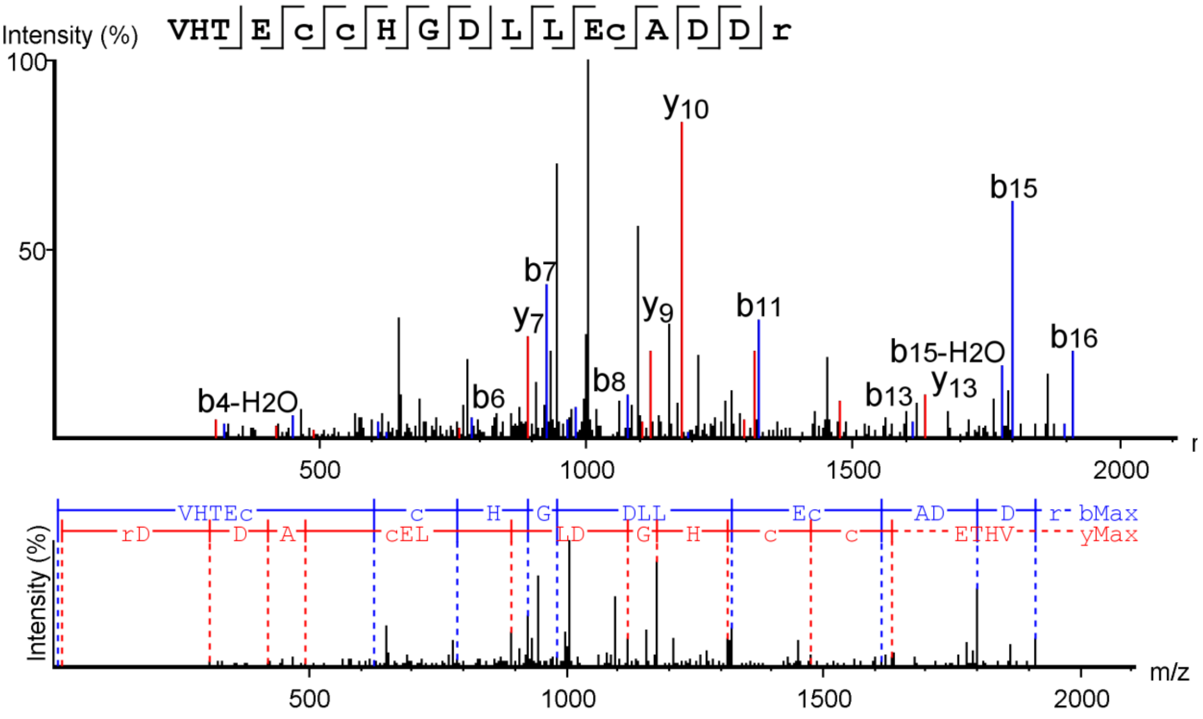
Intensity (%)
VHT E c c H G D L L Ec A D D r
100
50
y10
b15
b7
y7
y9
b11
b16
b15-H2O
y13
b13
b8
b6
b4-H2O
500
1000
1500
2000
VHTEc c H G DLL Ec AD D r bMax
rD D A cEL LD G H c c ETHV yMax
Intensity (%)
m/z

42

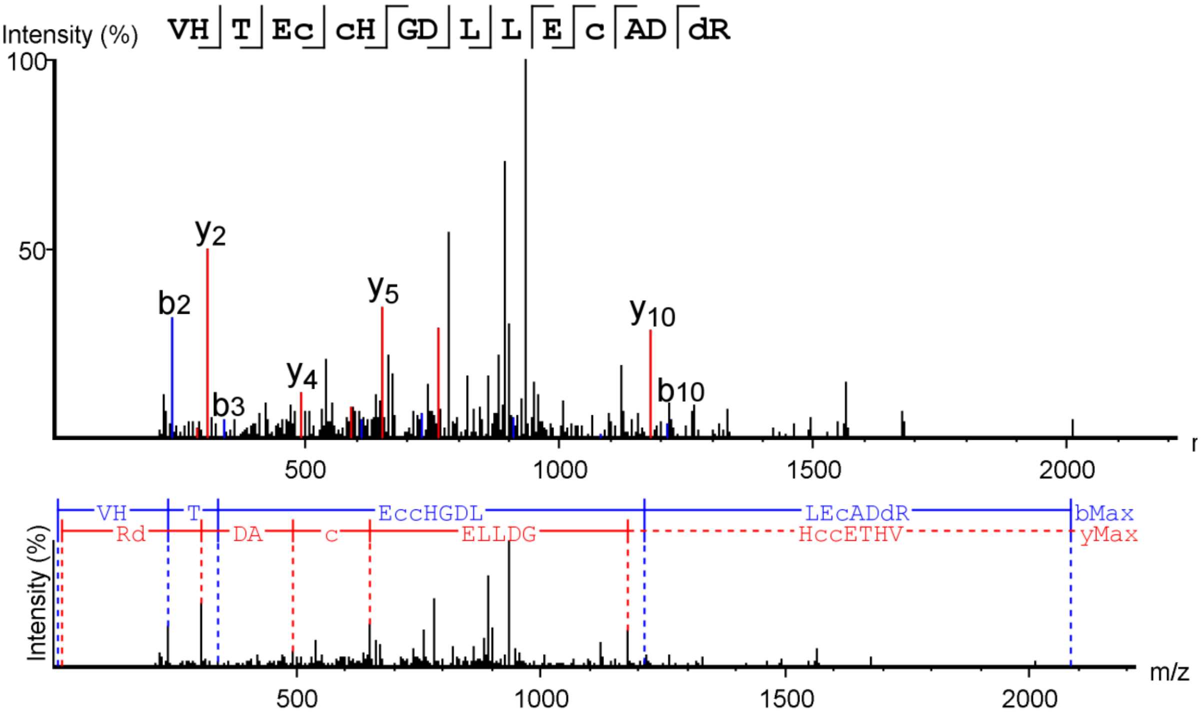
Intensity (%)
VH T Ec cH GD L L E c AD dR
100
50
y2
b2
y5
y10
y4
b3
b10
500
1000
1500
2000
VH T EccHGDL LEcADdR bMax
Rd DA c ELLDG HccETHV yMax
Intensity (%)
m/z

43

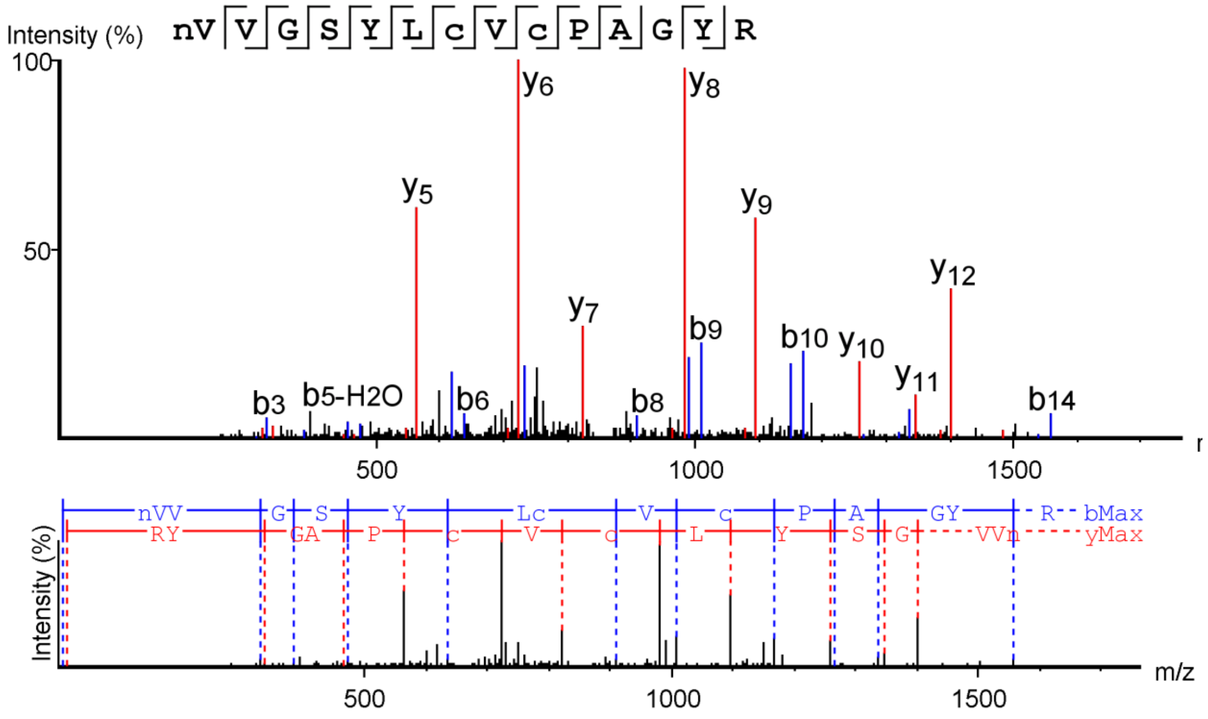
Intensity (%)
nV V G S Y L c V c P A G Y R
y6
y8
y5
y9
y12
y7
b9
b10
y10
y11
b3
b5-H2O
b6
b8
b14
nVV G S Y Lc V c P A GY R bMax
RY GA P c V c L Y S G VVn yMax
Intensity (%)
m/z

44

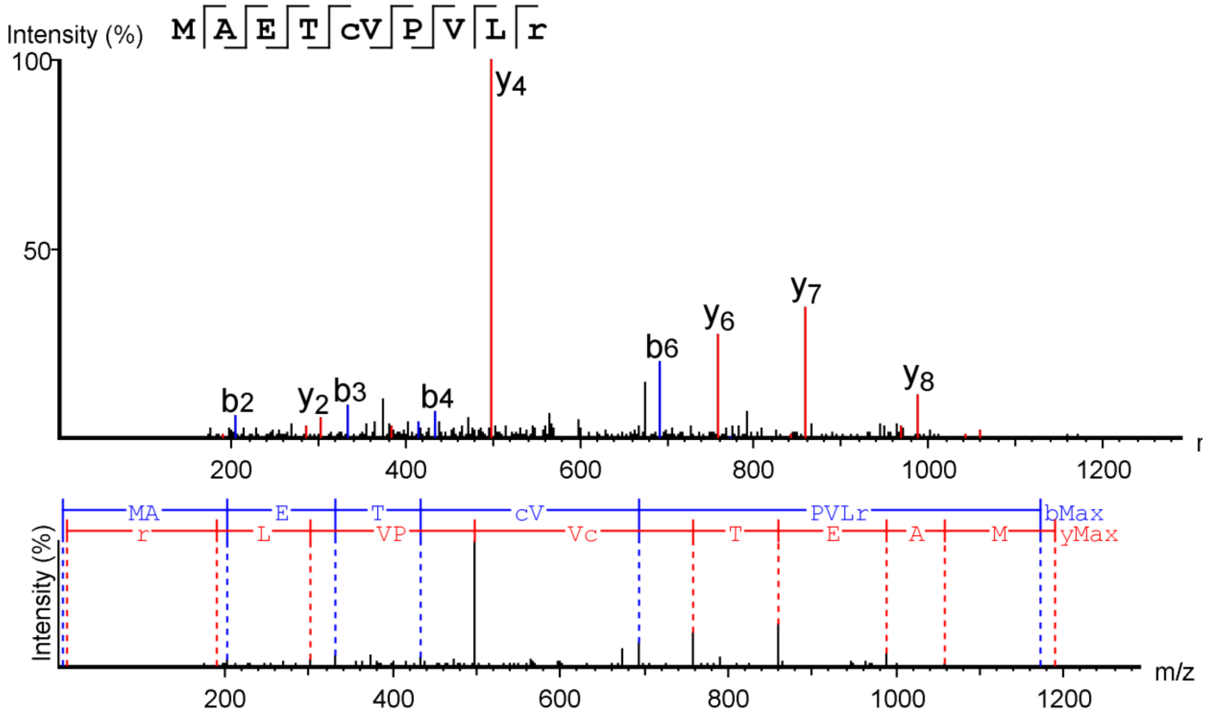
Intensity (%)
M A E T cV P V L r
y4
y7
y6
b6
y8
b2
y2
b3
b4
MA E T cV PVLr bMax
r L VP Vc T E A M yMax
Intensity (%)
m/z

45

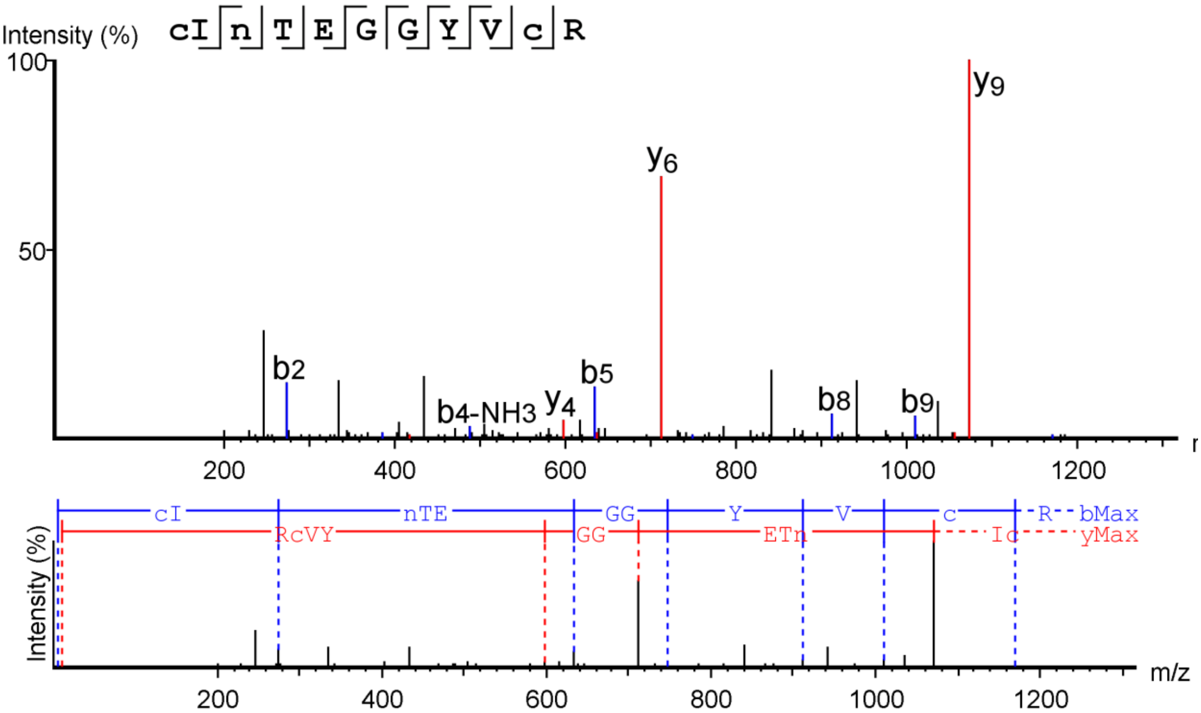
Intensity (%)
cI n T E G G Y V c R
100
50
y9
y6
b2
b5
b4-NH3
y4
b8
b9
200
400
600
800
1000
1200
cI nTE GG Y V c R bMax
RcVY GG ETn Ic yMax
Intensity (%)
m/z

46

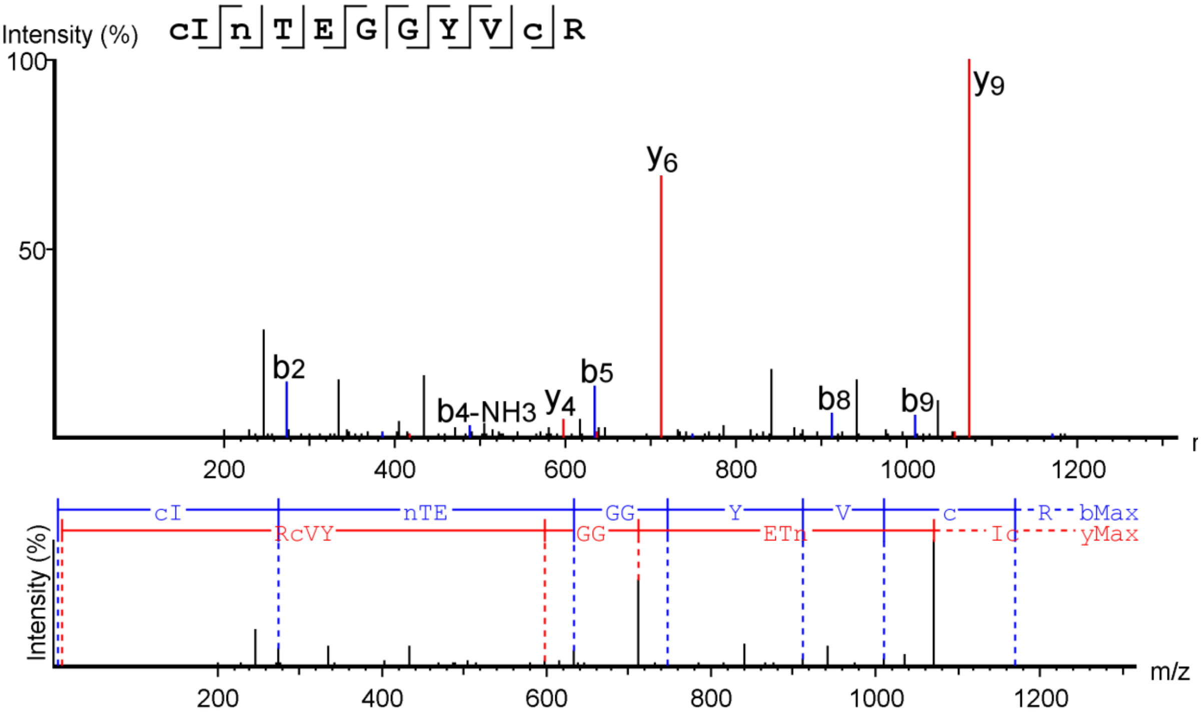
Intensity (%)
cI n T E G G Y V c R
100
50
y9
y6
b2
b5
b4-NH3
y4
b8
b9
200
400
600
800
1000
1200
cI nTE GG Y V c R bMax
RcVY GG ETn Ic yMax
Intensity (%)
m/z

47

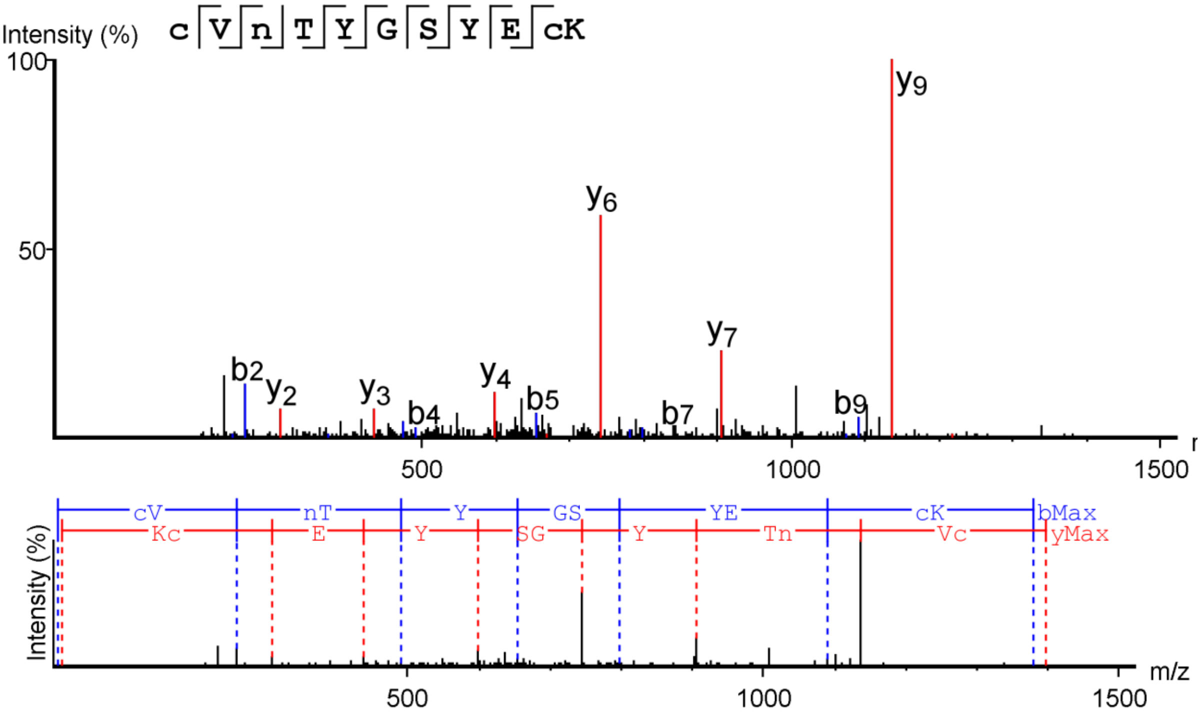
Intensity (%)
cVnTYGSYEcK
100
50
y9
y6
y7
b2
y2
y3
b4
y4
b5
b7
b9
500
1000
1500
cV
nT
Y
GS
YE
cK
bMax
Kc
E
Y
SG
Y
Tn
Vc
yMax
Intensity (%)
m/z
500
1000
1500

48

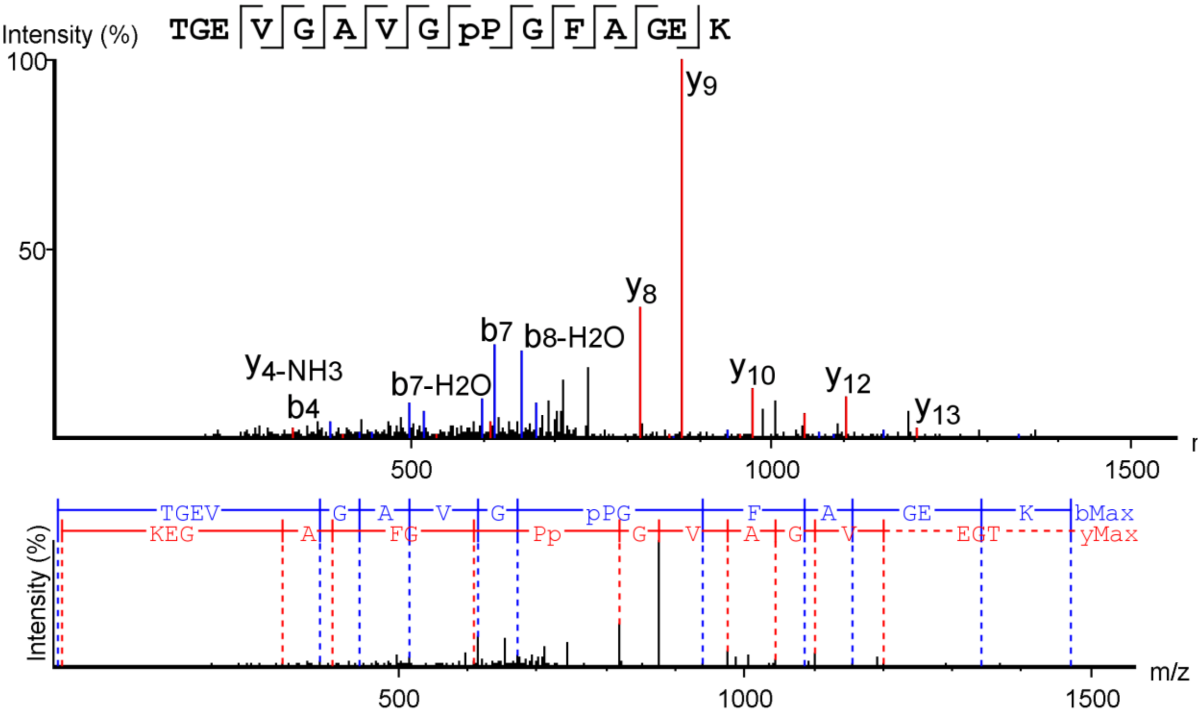
Intensity (%)
TGEVGAVGpPGFAGEK
100
50
y9
y8
b7
b8-H2O
y4-NH3
b4
b7-H2O
y10
y12
y13
500
1000
1500
TGEV
G
A
V
G
pPG
F
A
GE
K
bMax
KEG
A
FG
Pp
G
V
A
G
V
EGT
yMax
Intensity (%)
m/z
500
1000
1500

49

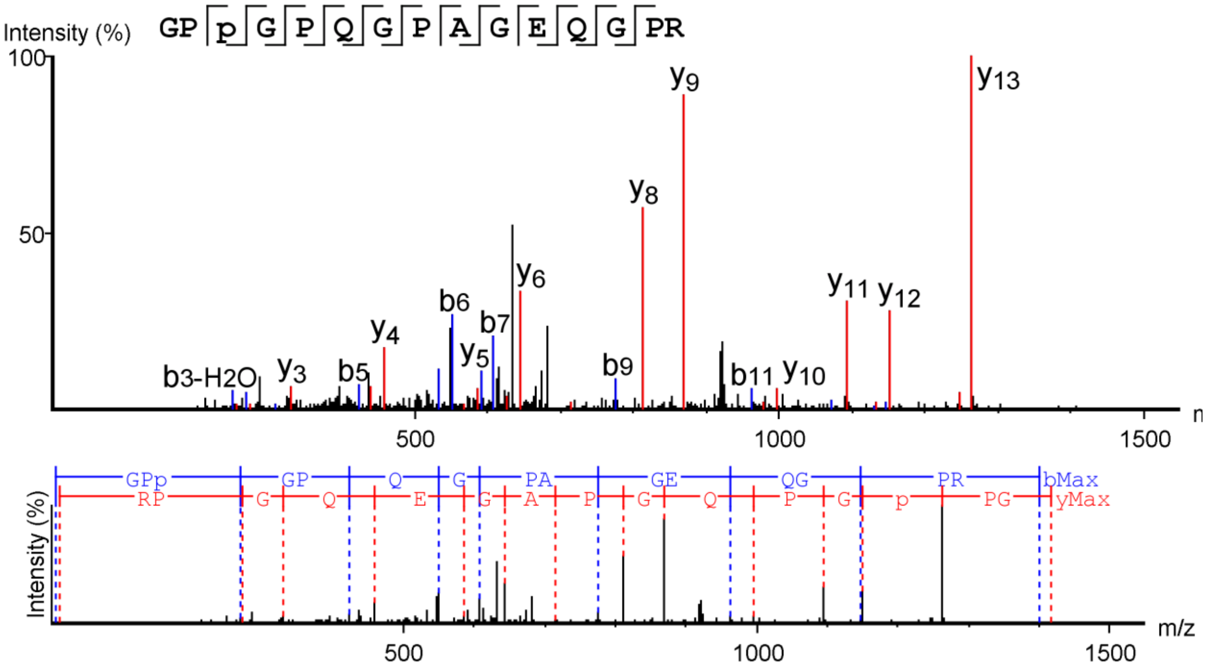
Intensity (%)
GP p G P Q G P A G E Q G PR
100
50
y9
y13
y8
y6
y11 y12
b6
b7
y4
y5
b3-H2O
y3
b5
b9
b11 y10
500
1000
1500
GPp GP Q G PA GE QG PR bMax
RP G Q E G A P G Q P G p PG yMax
Intensity (%)
m/z

50

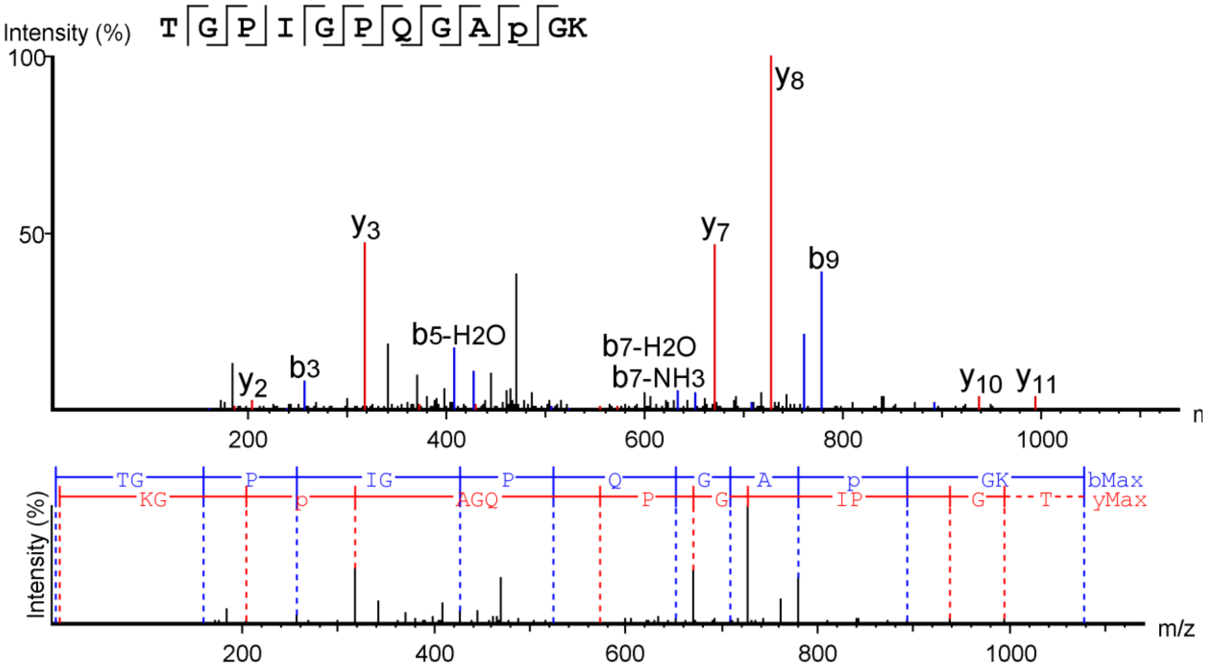
Intensity (%)
T G P I G P Q G A p GK
100
50
y8
y3
y7
b9
b5-H2O
b7-H2O
b7-NH3
b3
y2
y10 y11
200
400
600
800
1000
TG P IG P Q G A p GK bMax
KG p AGQ P G IP G T yMax
Intensity (%)
m/z

51

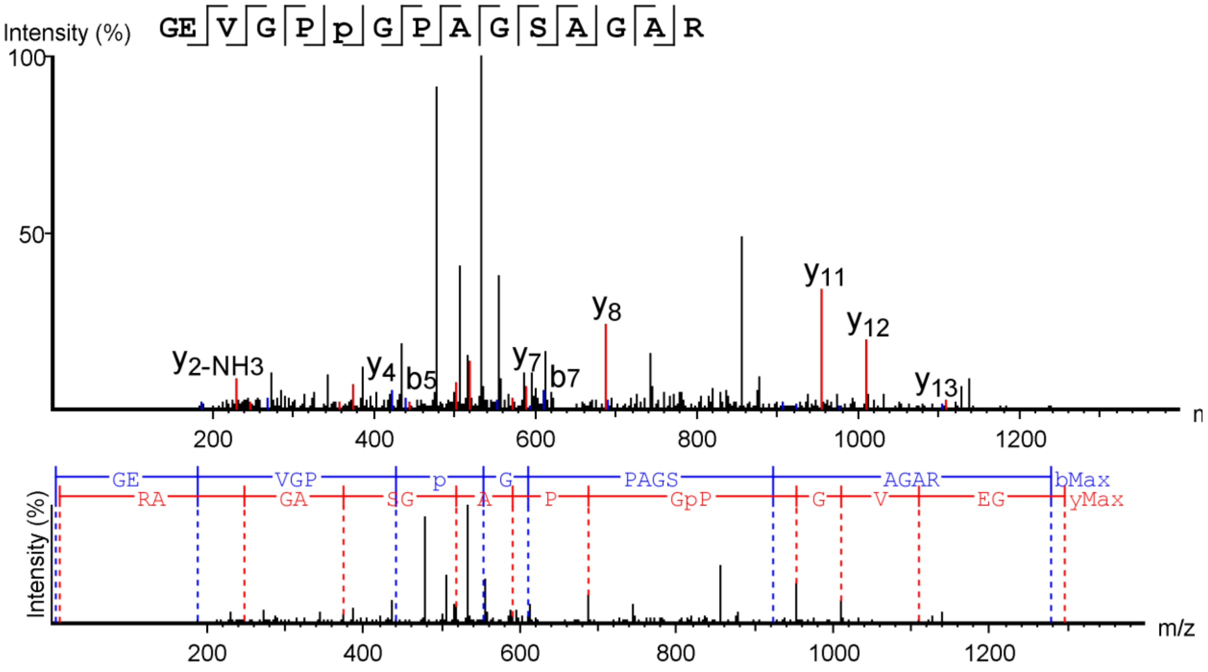
Intensity (%)
GE V G P p G P A G S A G A R
100
50
y2-NH3
y4
b5
y7
b7
y8
y11
y12
y13
200
400
600
800
1000
1200
GE
VGP
p
G
PAGS
AGAR
bMax
RA
GA
SG
A
P
GpP
G
V
EG
yMax
Intensity (%)
m/z

52

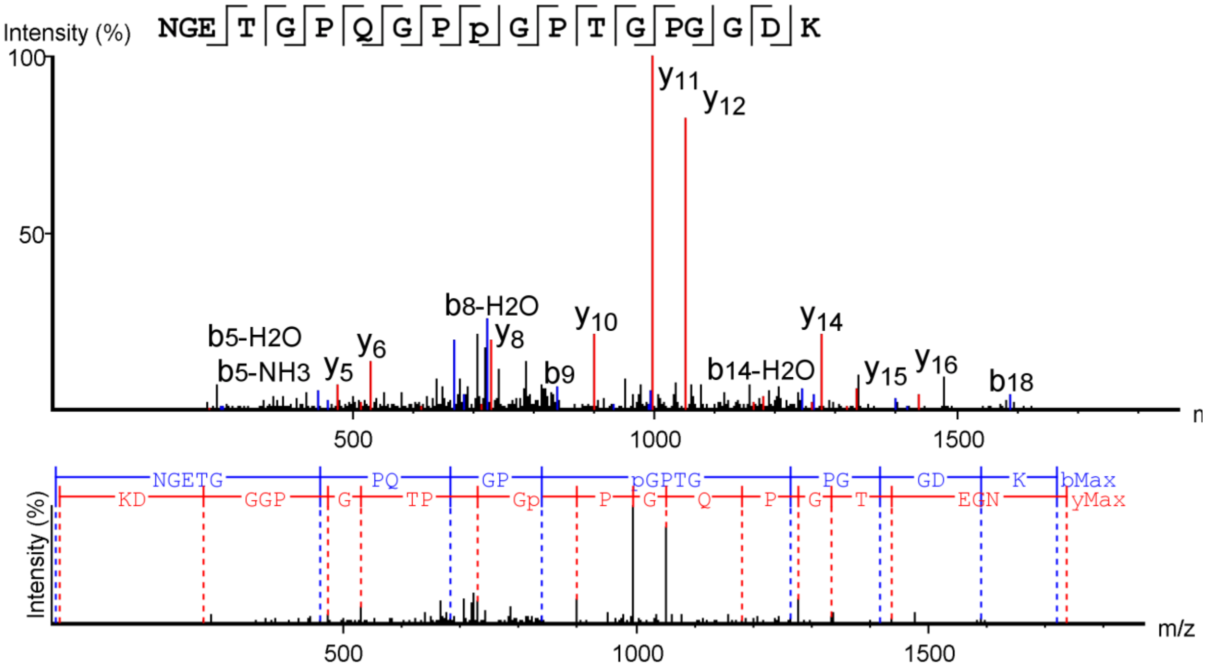
Intensity (%)
NGE T G P Q G P p G P T G PG G D K
100
50
b5-H2O
b5-NH3
y5
y6
b8-H2O
y8
b9
y10
y11
y12
b14-H2O
y14
y15
y16
b18
500
1000
1500
NGETG
PQ
GP
pGPTG
PG
GD
K
bMax
KD
GGP
G
TP
Gp
P
G
Q
P
G
T
EGN
yMax
Intensity (%)
m/z

53

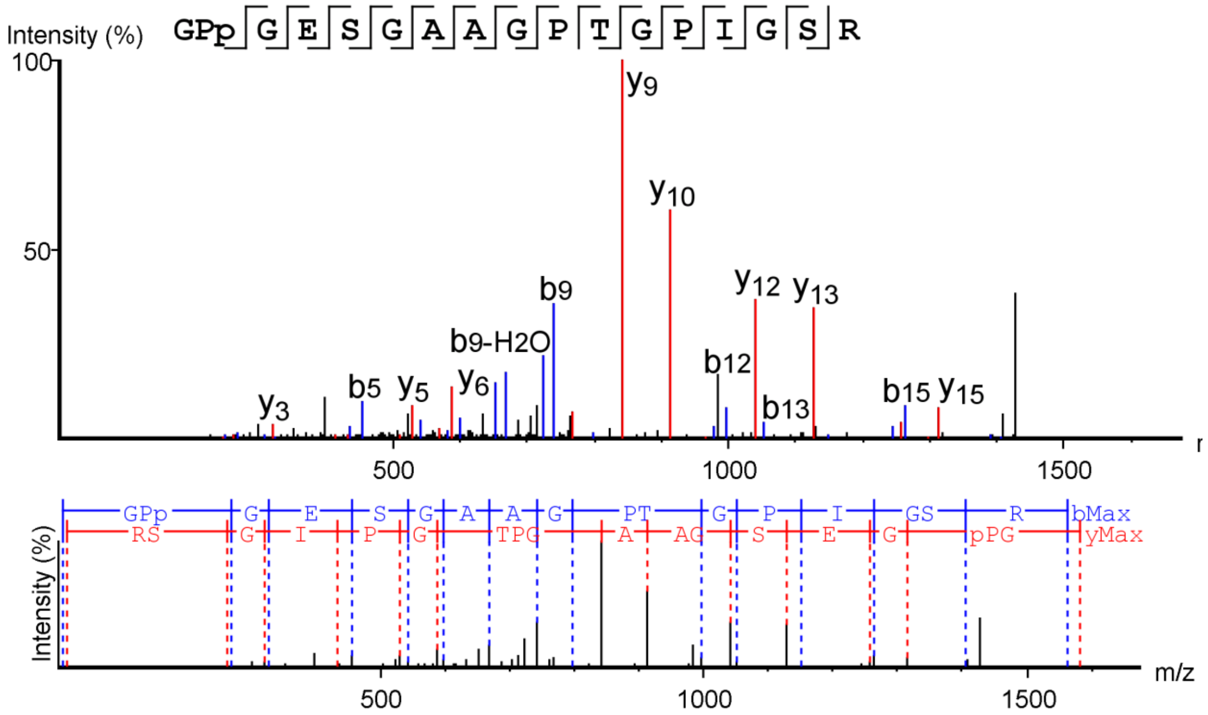
Intensity (%)
GPp G E S G A A G P T G P I G S R
100
50
y9
y10
y12 y13
b9
b9-H2O
b12
b5 y5 y6
y3
b13
b15 y15
500
1000
1500
GPp G E S G A A G PT G P I GS R bMax
RS G I P G TPG A AG S E G pPG yMax
Intensity (%)
m/z
500
1000
1500

54

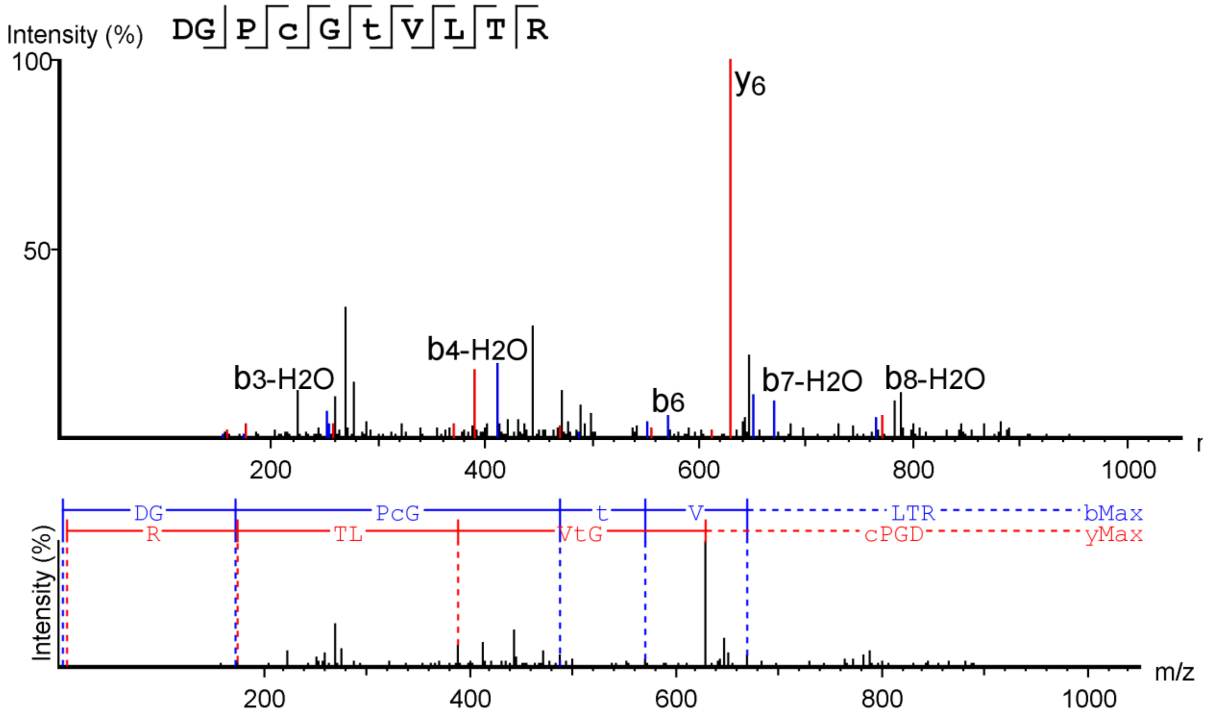
Intensity (%)
DG P c G t V L T R
100
50
y6
b3-H2O
b4-H2O
b6
b7-H2O
b8-H2O
200
400
600
800
1000
DG PcG t V LTR bMax
R TL VtG cPGD yMax
Intensity (%)
m/z
200
400
600
800
1000

55

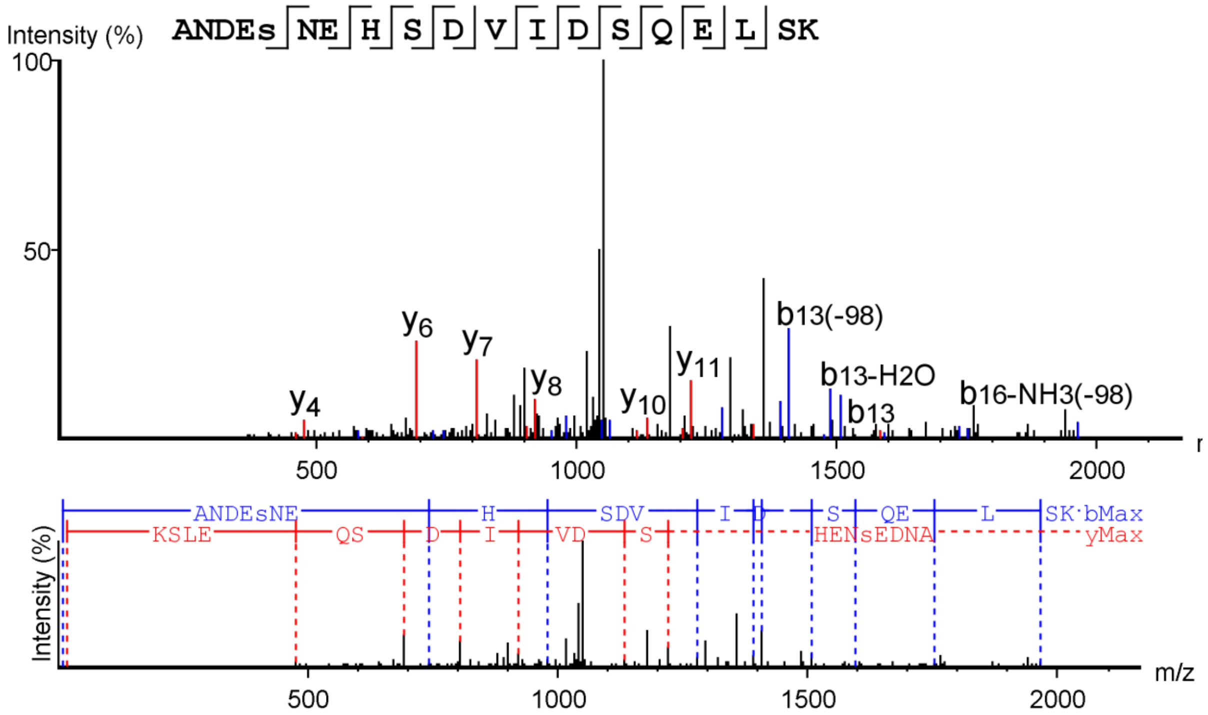
ANDEs NE H S D V I D S Q E L SK
Intensity (%)
100
50
y4
y6
y7
y8
y10
y11
b13(-98)
b13-H2O
b13
b16-NH3(-98)
500
1000
1500
2000
ANDEsNE
H
SDV
I
D
S
QE
L
SK
bMax
KSLE
QS
D
I
VD
S
HENsEDNA
yMax
Intensity (%)
m/z

56

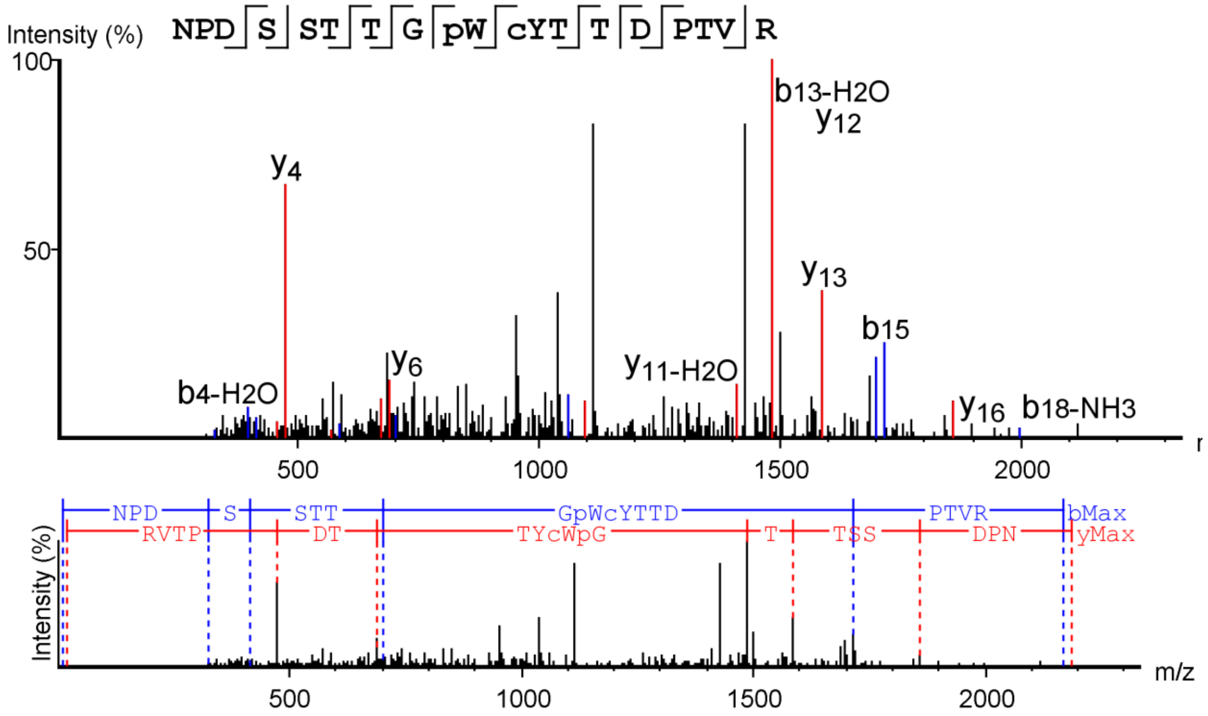
NPD S ST T G pW cYT T D PTV R
Intensity (%)
100
50
b4-H2O
y4
y6
y11-H2O
b13-H2O
y12
y13
b15
y16
b18-NH3
500
1000
1500
2000
NPD
S
STT
GpWcYTTD
PTVR
bMax
RVTP
DT
TYcWpG
T
TSS
DPN
yMax
Intensity (%)
m/z

57

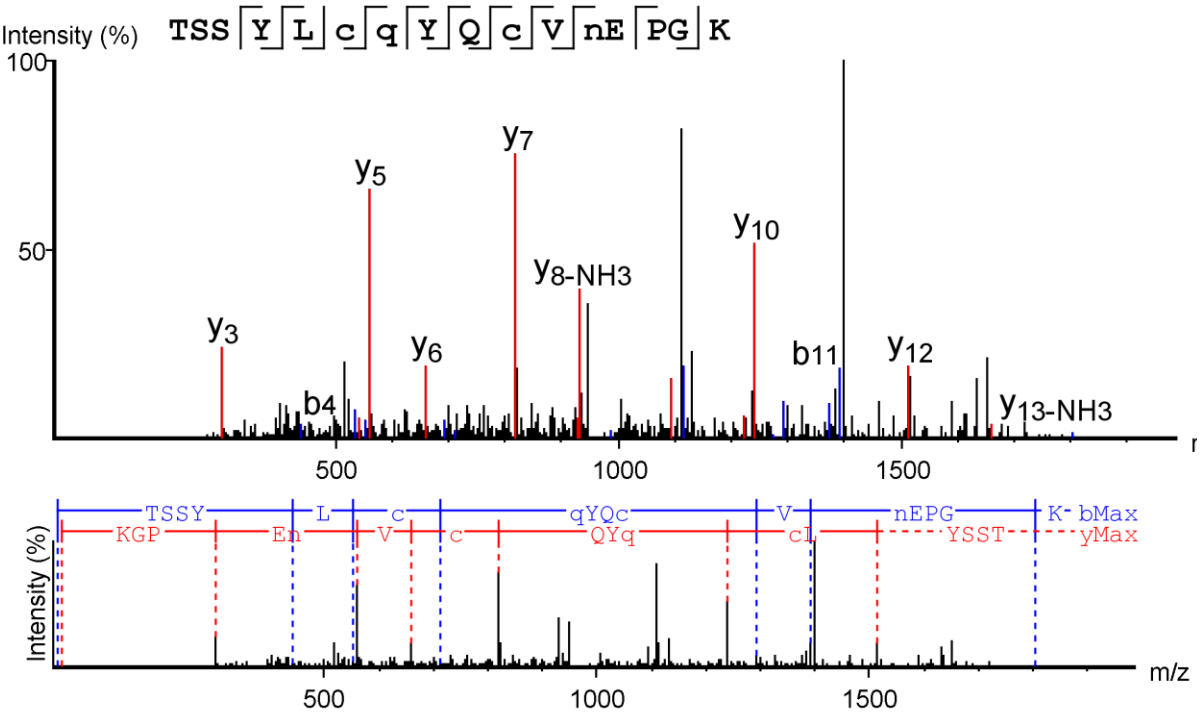
Intensity (%)
TSS Y L c q Y Q c V nE PG K
100
50
y3
b4
y5
y6
y7
y8-NH3
y10
b11
y12
y13-NH3
500
1000
1500
TSSY L c qYQc V nEPG K bMax
KGP En V c QYq cL YSST yMax
Intensity (%)
m/z

58

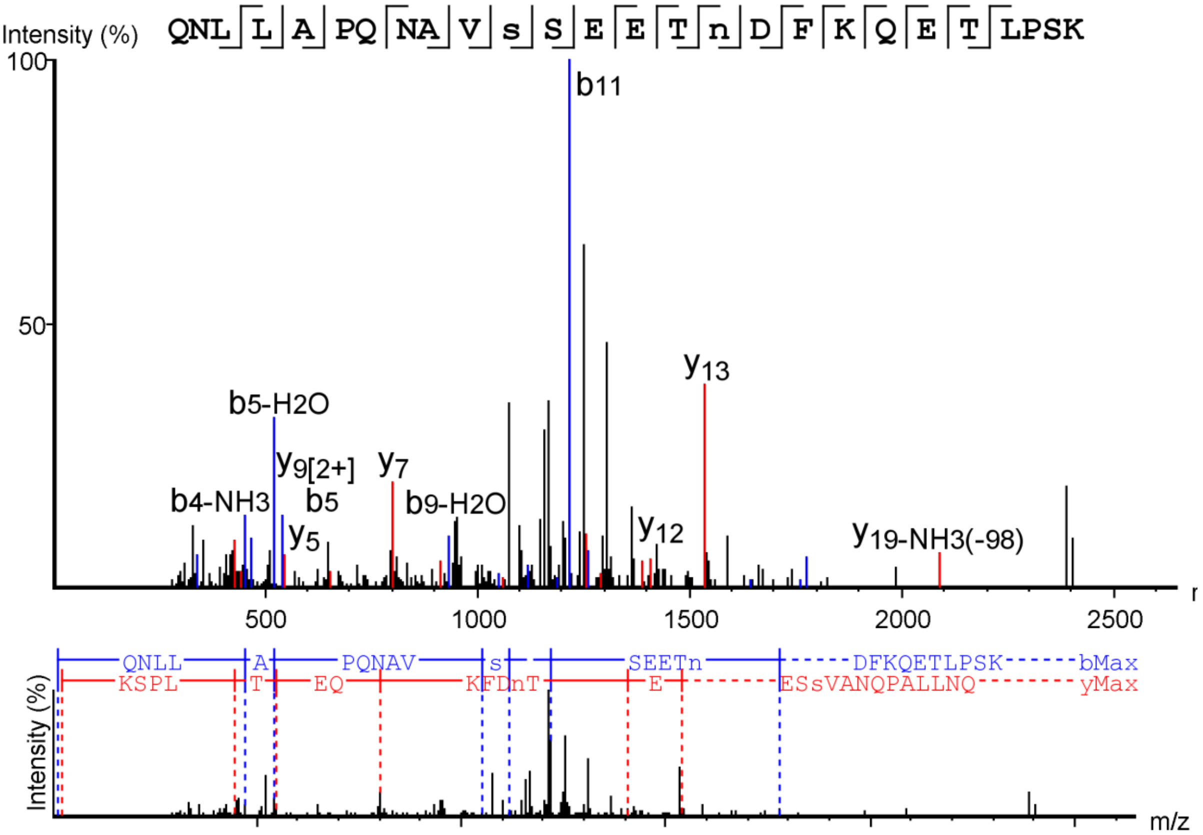
Intensity (%)
QNL L A PQ NA V s S E E T n D F K Q E T LPSK
100
50
b11
y13
b5-H2O
y9[2+]
y7
b4-NH3
b5
b9-H2O
y5
y12
y19-NH3(-98)
500
1000
1500
2000
2500
QNLL A PQNAV s SEETn DFKQETLPSK bMax
KSPL T EQ KFDnT E ESsVANQPALLNQ yMax
Intensity (%)
m/z

59

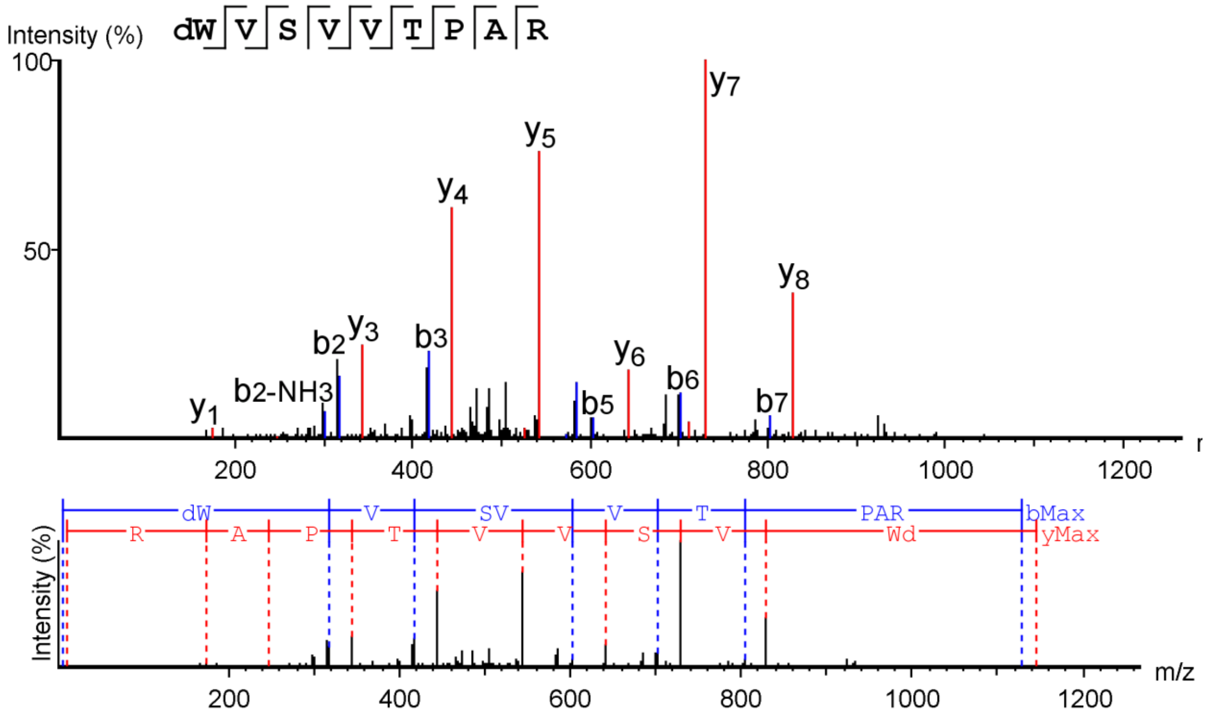
Intensity (%)
dW V S V V T P A R
y7
y5
y4
y8
b2 y3
b3
y6
b6
b2-NH3
b5
b7
y1
dW V SV V T PAR bMax
R A P T V V S V Wd yMax
Intensity (%)
m/z

60

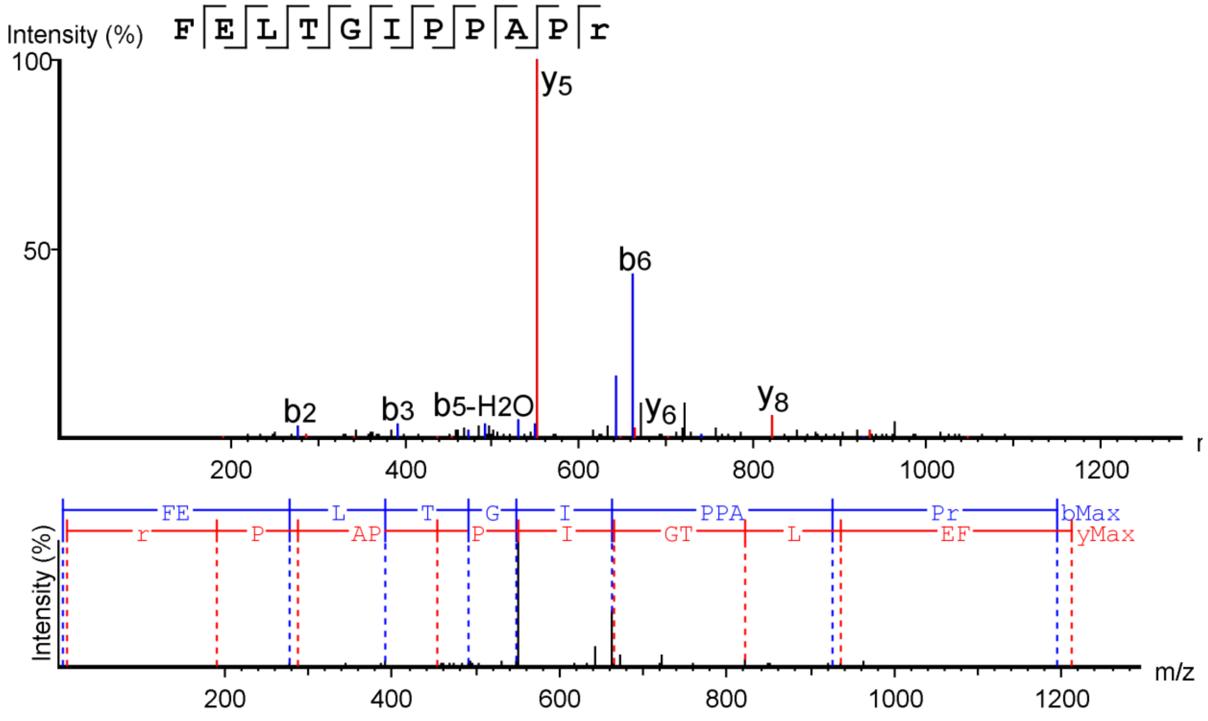
Intensity (%)
F E L T G I P P A P r
y5
b6
y8
b2
b3
b5-H2O
y6
FE L T G I PPA Pr bMax
r P AP P I GT L EF yMax
Intensity (%)
m/z

61

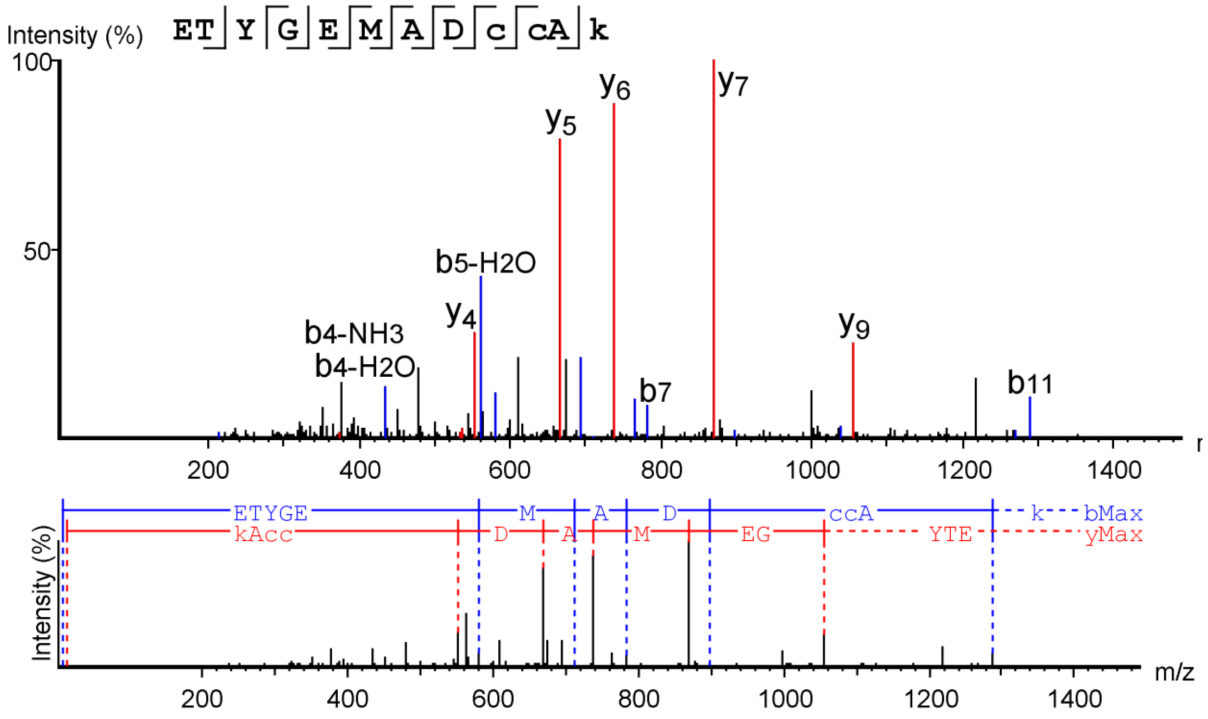
Intensity (%)
ETYGEMADccAk
100
50
y7
y6
y5
b5-H2O
y4
b4-NH3
b4-H2O
y9
b7
b11
200
400
600
800
1000
1200
1400
ETYGE
M
A
D
ccA
k
bMax
kAcc
D
A
M
EG
YTE
yMax
Intensity (%)
m/z

62

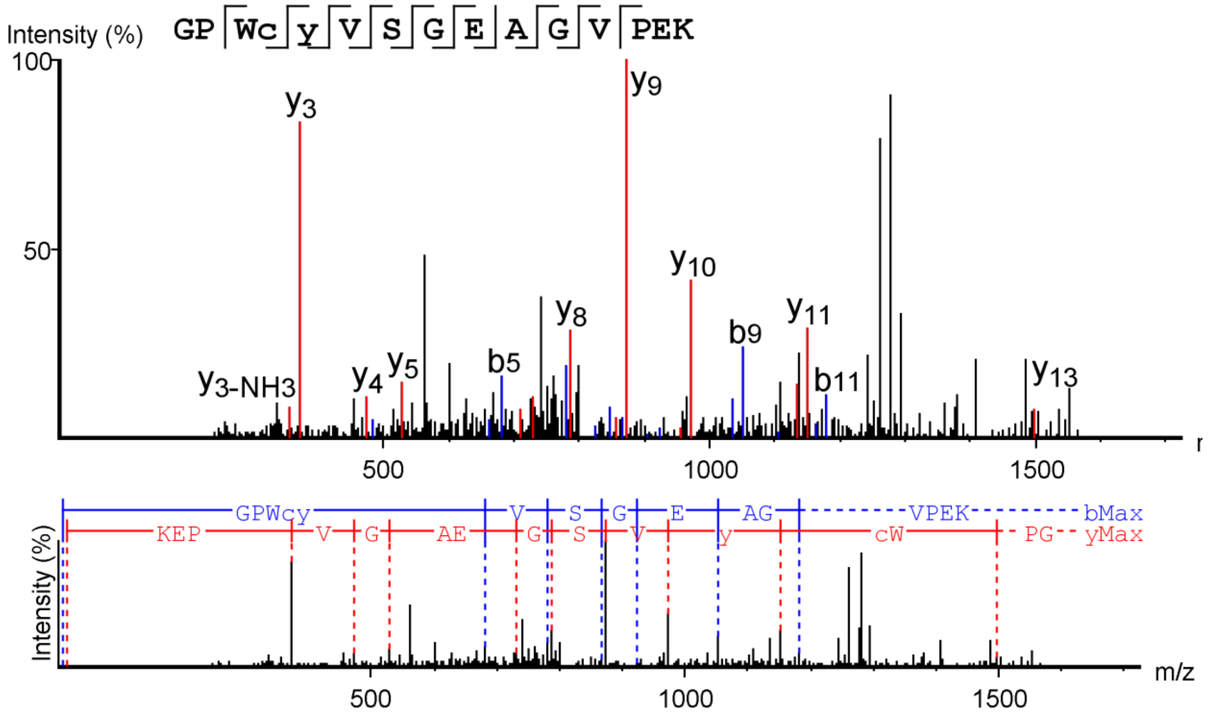
Intensity (%)
GPWcyVSGEAGVPEK
100
50
y3
y9
y10
y8
b9
y11
y3-NH3
y4
y5
b5
b11
y13
500
1000
1500
GPWcy
V
S
G
E
AG
VPEK
bMax
KEP
V
G
AE
G
S
V
y
cW
PG
yMax
Intensity (%)
m/z

63

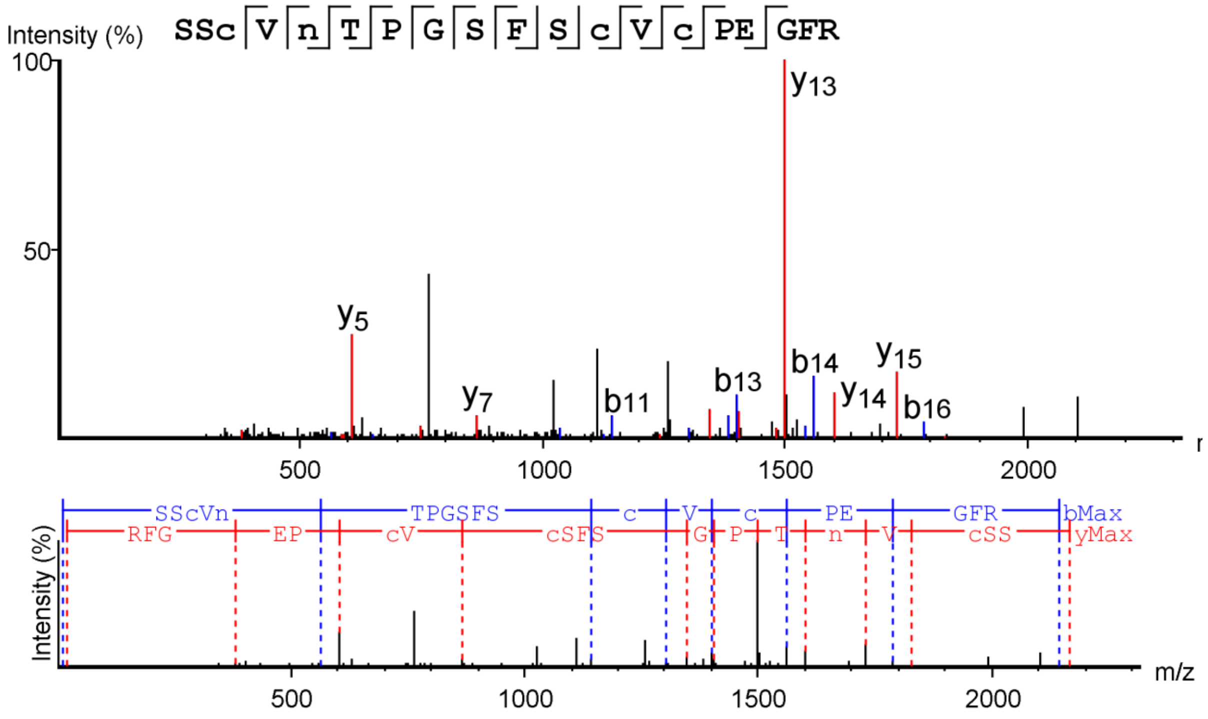

Intensity (%)
SSc V n T P G S F S c V c PE GFR
100
50
y13
y5
y7
b11
b13
b14
y14
y15
b16
500
1000
1500
2000
SScVn
TPGSFS
c
V
c
PE
GFR
bMax
RFG
EP
cV
cSFS
G
P
T
n
V
cSS
yMax
Intensity (%)
500
1000
1500
2000
m/z

64

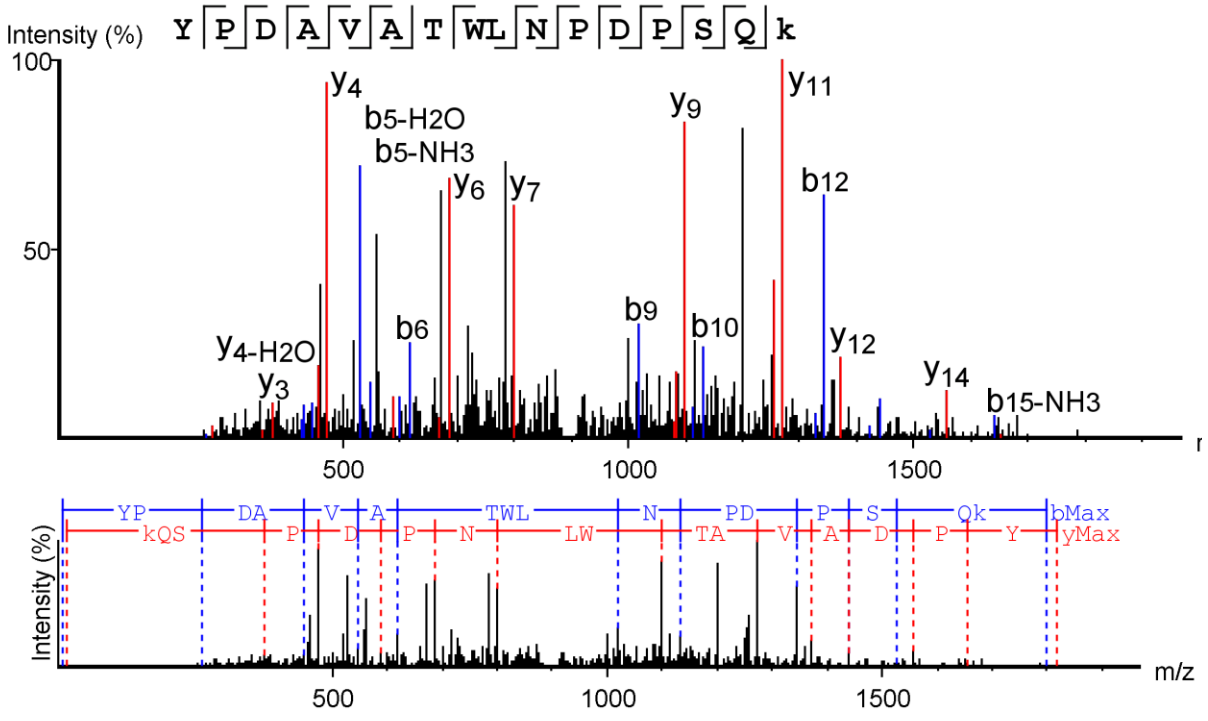

Intensity (%)
Y P D A V A T WL N P D P S Q k
100
50
y4
b5-H2O
b5-NH3
y6
y7
y9
y11
b12
y4-H2O
y3
b6
b9
b10
y12
y14
b15-NH3
500
1000
1500
YP
DA
V
A
TWL
N
PD
P
S
Qk
bMax
kQS
P
D
P
N
LW
TA
V
A
D
P
Y
yMax
Intensity (%)
500
1000
1500
m/z

65

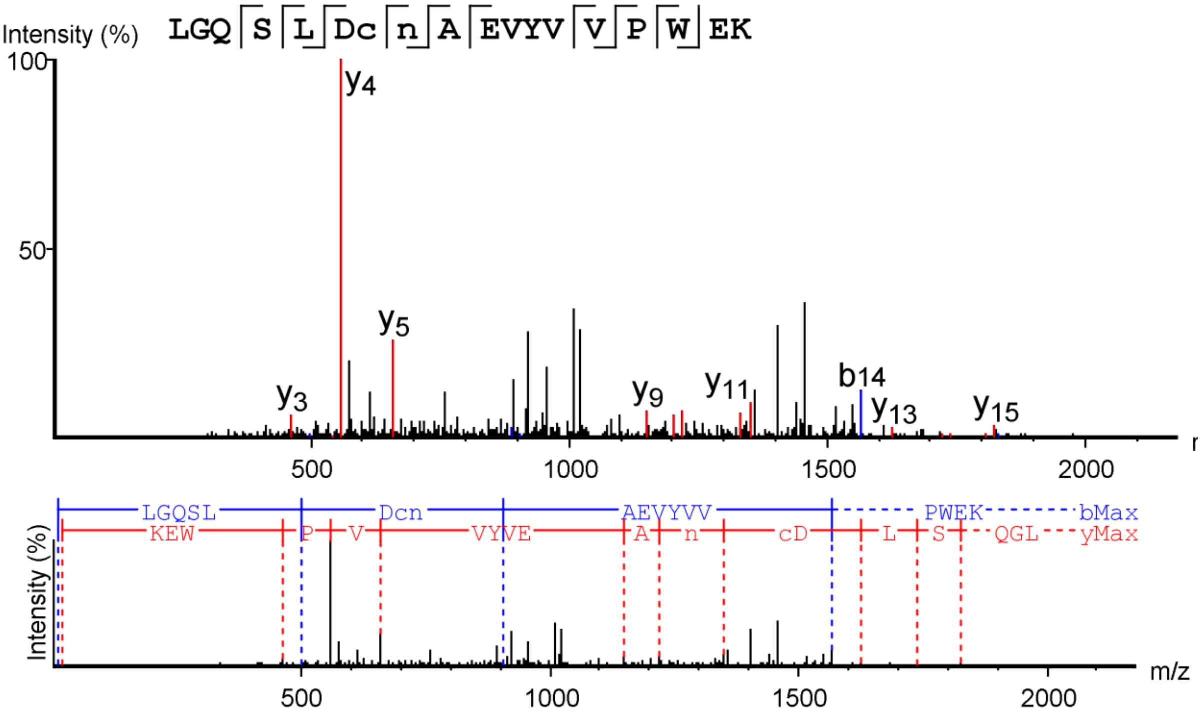
Intensity (%)
LGQ S L Dc n A EVYV V P W EK
100
50
y4
y5
y3
y9
y11
b14
y13
y15
500
1000
1500
2000
LGQSL
Dcn
AEVYVV
PWEK
bMax
KEW
P
V
VYVE
A
n
cD
L
S
QGL
yMax
Intensity (%)
m/z

66

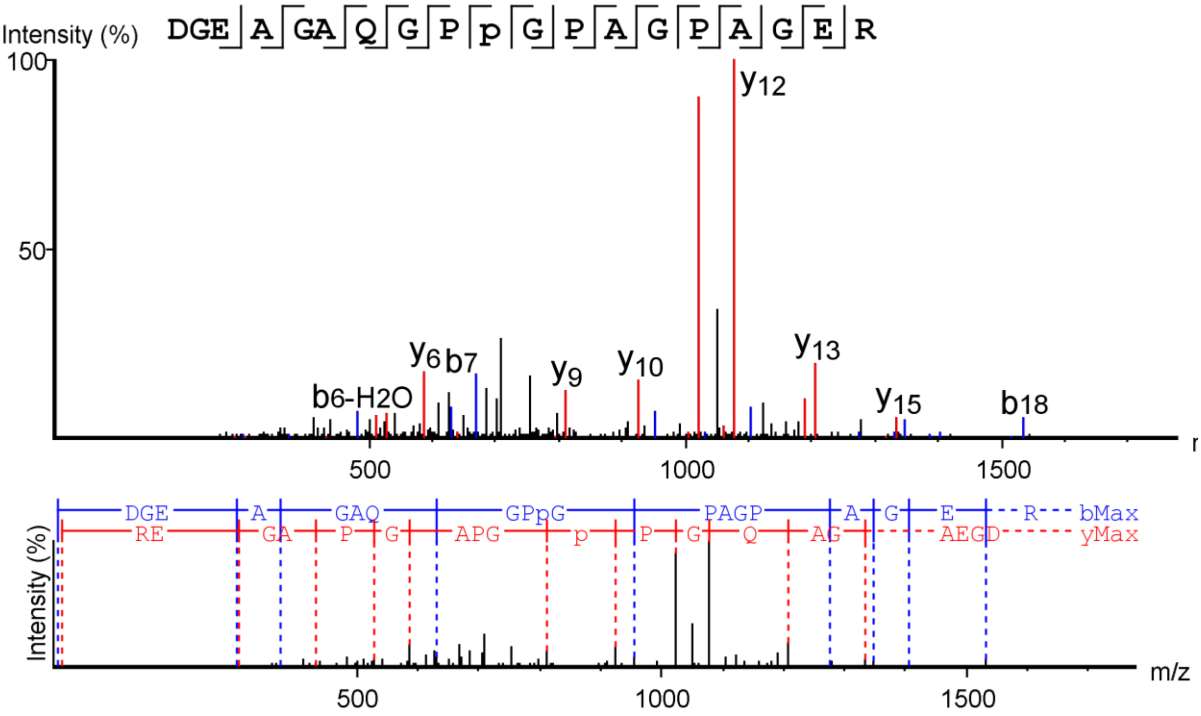
Intensity (%)
DGE A GA Q G P p G P A G P A G E R
100
50
y12
y13
y6
b7
b6-H2O
y9
y10
y15
b18
500
1000
1500
DGE
A
GAQ
GPpG
PAGP
A
G
E
R
bMax
RE
GA
P
G
APG
p
P
G
Q
AG
AEGD
yMax
Intensity (%)
m/z

67

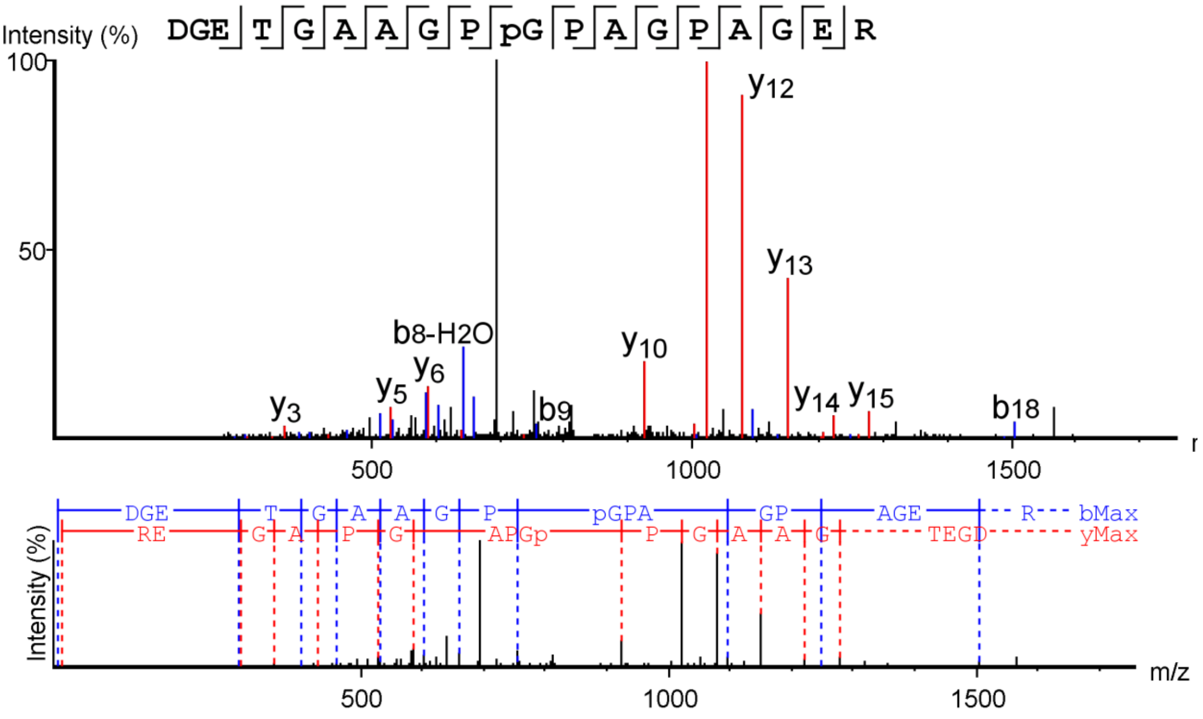
Intensity (%)
DGE T G A A G P pG P A G P A G E R
100
50
y12
y13
y10
b8-H2O
y6
y5
y3
b9
y14 y15
b18
500
1000
1500
DGE T G A A G P pGPA GP AGE R bMax
RE G A P G APGp P G A A G TEGD yMax
Intensity (%)
m/z
500
1000
1500

68

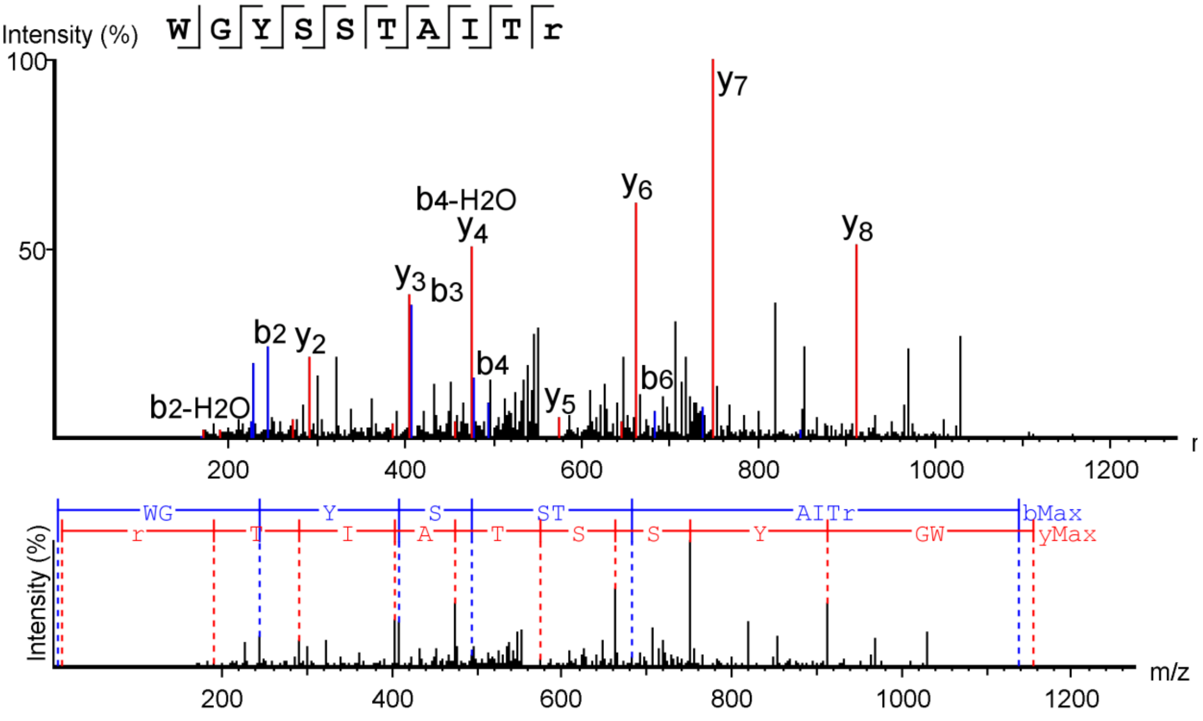
Intensity (%)
W G Y S S T A I T r
100
50
y7
y6
y8
b4-H2O
y4
y3 b3
b2 y2
b4
y5
b6
b2-H2O
200
400
600
800
1000
1200
WG Y S ST AITr bMax
r T I A T S S Y GW yMax
Intensity (%)
m/z
200
400
600
800
1000
1200

69

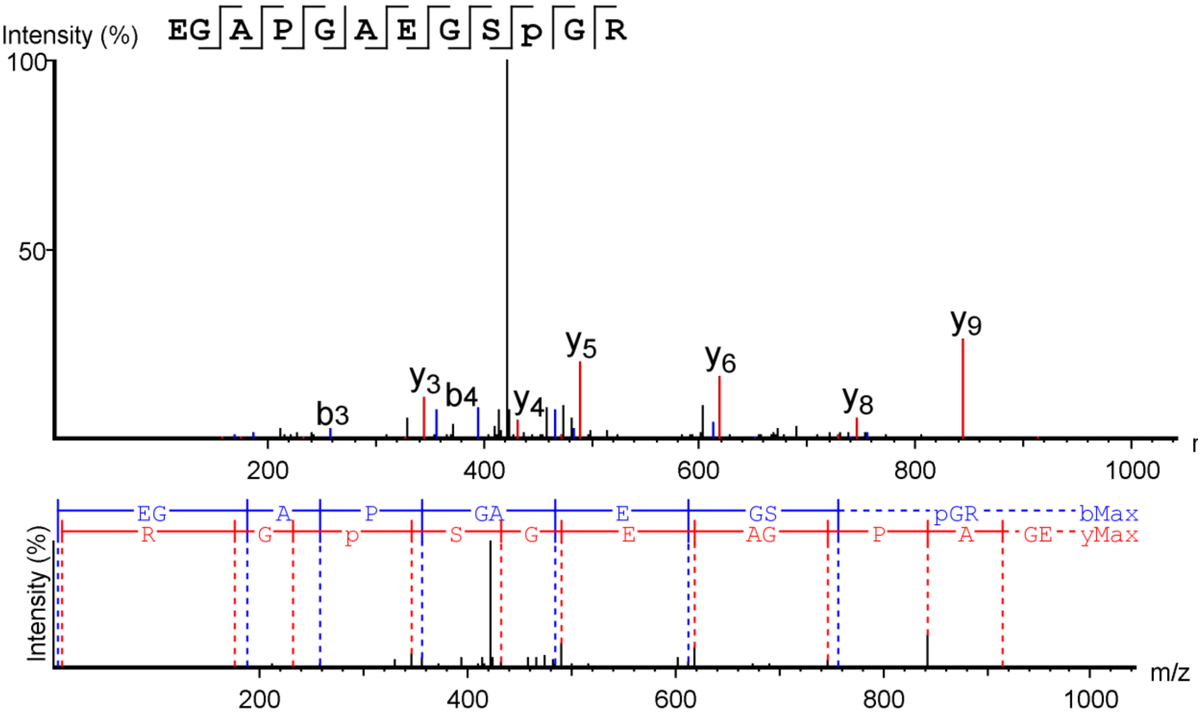
Intensity (%)
EG A P G A E G S p G R
100
50
b3
y3
b4
y4
y5
y6
y8
y9
200
400
600
800
1000
EG A P GA E GS pGR bMax
R G p S G E AG P A GE yMax
Intensity (%)
m/z

70

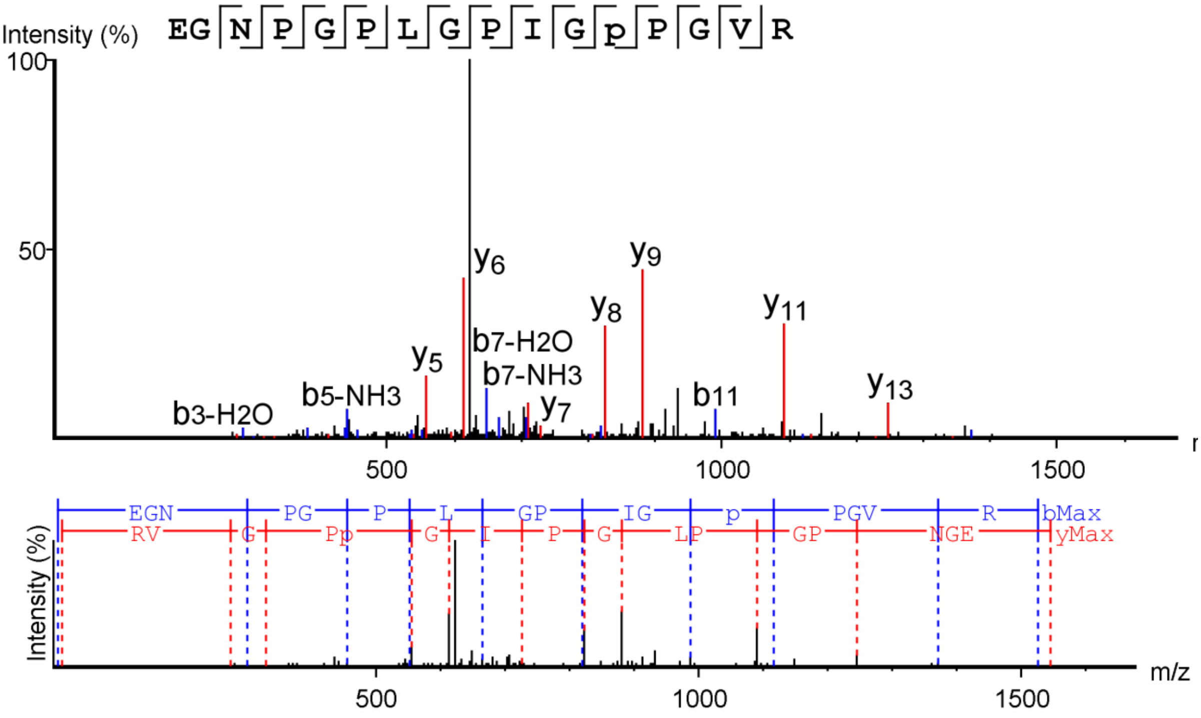
Intensity (%)
EG N P G P L G P I G p P G V R
100
50
b3-H2O
b5-NH3
y5
y6
b7-H2O
b7-NH3
y7
y8
y9
b11
y11
y13
500
1000
1500
EGN PG P L GP IG p PGV R bMax
RV G Pp G I P G LP GP NGE yMax
Intensity (%)
m/z

71

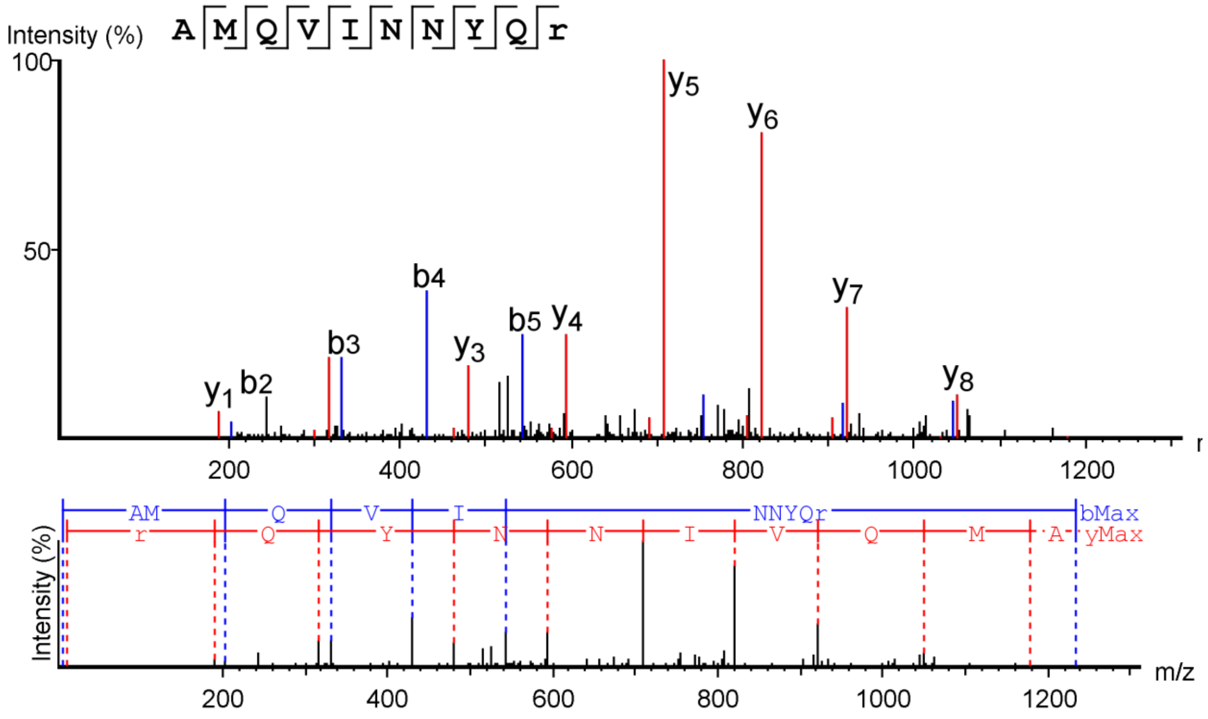

Intensity (%)
A M Q V I N N Y Q r
y5
y6
y7
y8
b4
b3
b5
y4
y3
y1
b2
AM Q V I NNYQr bMax
r Q Y N N I V Q M A yMax
Intensity (%)
m/z

72

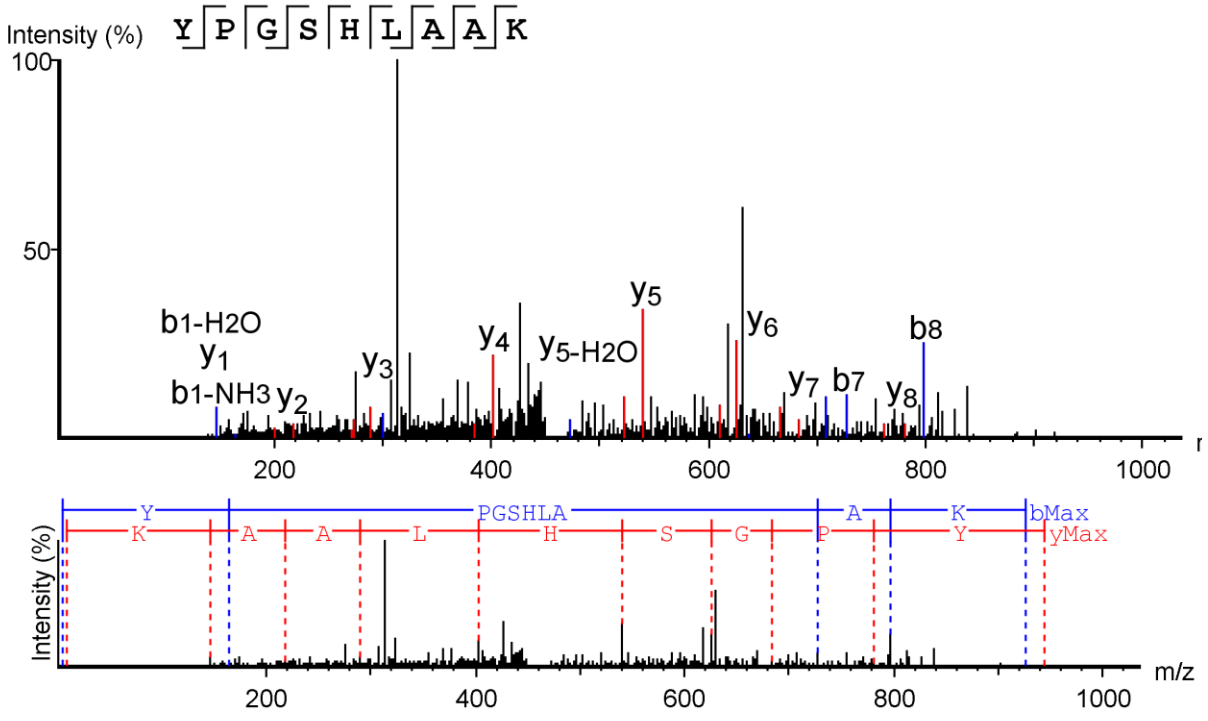

Intensity (%)
Y P G S H L A A K
y5
y6
b8
y4
y5-H2O
b1-H2O
y1
y3
b1-NH3
y2
y7
b7
y8
Y PGSHLA A K bMax
K A A L H S G P Y yMax
Intensity (%)
m/z

73

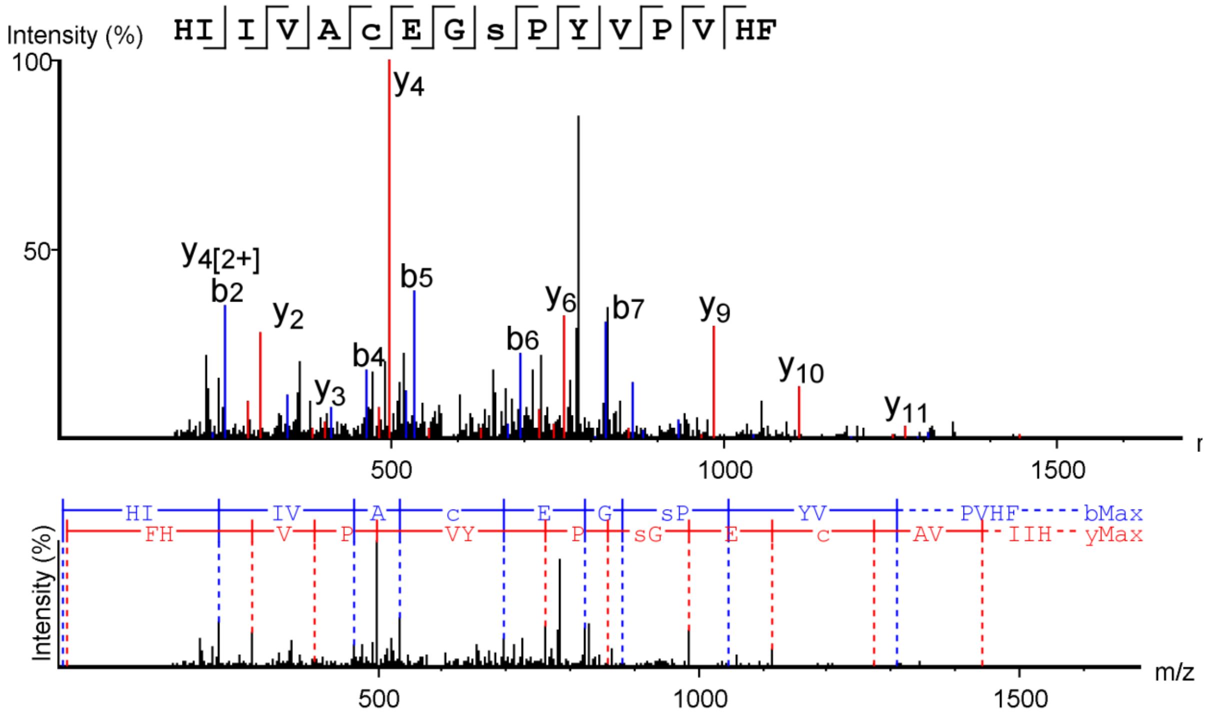
Intensity (%)
HI I V A c E G s P Y V P V HF
100
50
y4
y4[2+]
b2
y2
y3
b4
b5
b6
y6
b7
y9
y10
y11
500
1000
1500
HI IV A c E G sP YV PVHF bMax
FH V P VY P sG E c AV IIH yMax
Intensity (%)
m/z

74

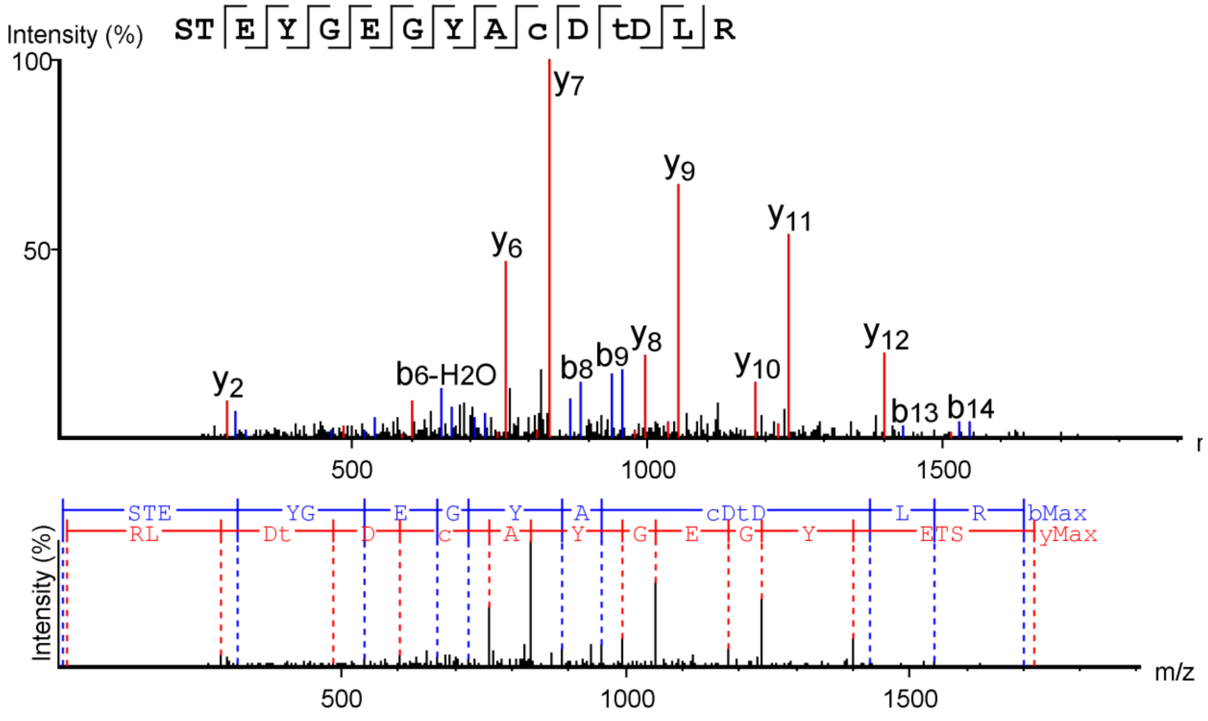
Intensity (%)
ST E Y G E G Y A c D tD L R
100
50
y7
y9
y6
y11
y12
y2
b6-H2O
b8
b9
y8
y10
b13
b14
500
1000
1500
STE YG E G Y A cDtD L R bMax
RL Dt D c A Y G E G Y ETS yMax
Intensity (%)
m/z

75

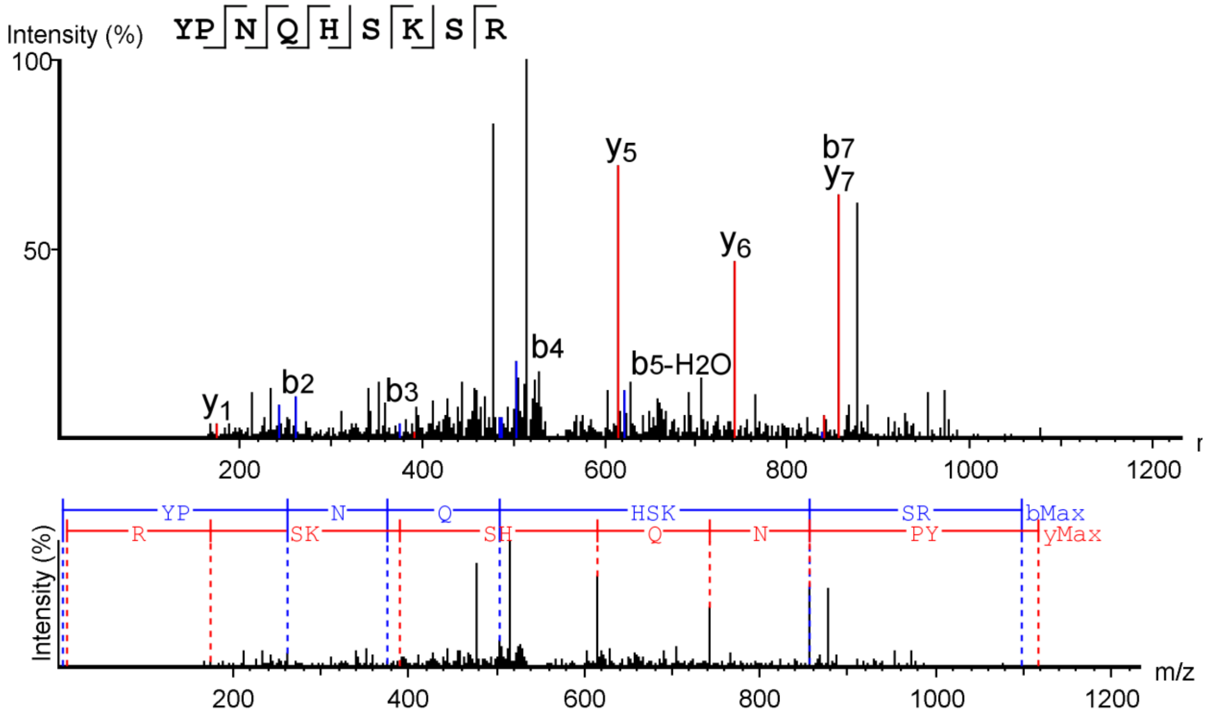

Intensity (%)
YP N Q H S K S R
100
50
y1
b2
b3
b4
y5
b5-H2O
y6
b7
y7
200
400
600
800
1000
1200
YP
N
Q
HSK
SR
bMax
R
SK
SH
Q
N
PY
yMax
Intensity (%)
m/z
